# Tunable bis(pyridinium amidate) ligands efficiently promote palladium-catalyzed ethylene polymerization

**DOI:** 10.1039/d5cy01102g

**Published:** 2025-10-30

**Authors:** Esaïe Reusser, Martin Albrecht

**Affiliations:** a Department of Chemistry, Biochemistry and Pharmaceutical Sciences, University of Bern Freiestrasse 3 3012 Bern Switzerland martin.albrecht@unibe.ch

## Abstract

A useful strategy for the co-polymerization of ethylene and functional olefins relies on palladium catalysts, as palladium typically shows in contrast to many other metals a high tolerance to a variety of functional groups. Here we have prepared a set of palladium complexes containing a *N*,*N*-bidentate coordinating bis(pyridinium amidate) (bisPYA) ligand. Ligand variation included either *para*- or an *ortho*-pyridinium amidate arrangement, with the pyridinium site either sterically flexible or locked through a dimethyl substitution *ortho* to the amidate. Activation of these complexes with NaBArF in the presence of ethylene indicated that sterically locked ligand structures promoted ethylene conversion and produced polymeric materials. In particular, complex 4d with an *ortho*-pyridinium amidate bisPYA ligand was active with a production of 10.8 kg polyethylene per mol palladium at room temperature and 1 bar ethylene. Synthesis of the complexes in the presence of K_2_CO_3_ or Ag_2_CO_3_ afforded adducts in which the K^+^ or Ag^+^ ion is bound by the two oxygens of the bisamidate core, thus leading to trimetallic Pd⋯K⋯Pd complexes. Such adduct formation indicates a dual role of NaBArF in halide abstraction and metal sequestration, thus rationalizing the need for 2.5 equivalent of NaBArF per palladium complex for effective polymerization.

## Abstract 2

Palladium complexes with symmetric *N*,*N*-bidentate bis(pyridinium amidate) ligands catalyze the olefin polymerization already at mild conditions, when the pyridinium site is *ortho*,*ortho*-dimethylated. The carbonyl groups engage in metal coordination.

## Graphical abstract

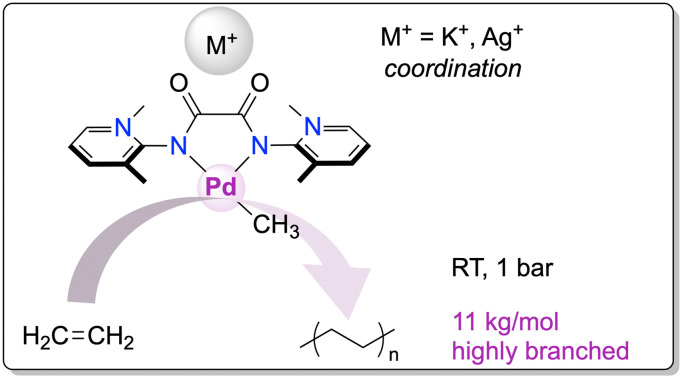

## Introduction

**Fig. 1:**
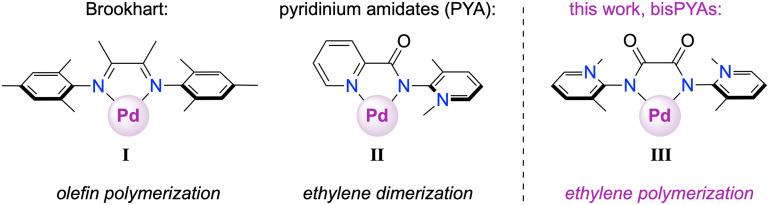
Structural comparison between the state-of-the art palladium olefin (co)polymerization precatalyst supported by α-diimine ligands (I), pyridyl-PYA ligands (II) inducing di- and oligomerization, and bisPYA ligands investigated in this work.

A comparison of classic palladium-based catalysts for olefin (co)polymerization, such as those reported by Brookhart,^1^ Drent^2^ and others^3,4^ has revealed that sterically adaptable and strongly electron-donating ligands such as I (Fig. 1) are beneficial and in fact essential for the production of polymeric materials.^5–11^ Building on these findings, pyridinium amidate (PYA) ligands that combine the structural versatility of these systems with strong donating properties have been proposed as an alternative to address specific challenges in Pd-catalyzed polymerization.^12^ The adaptable PYA scaffold is attractive for modulating both electronically and sterically the metal coordination sphere,^13^ making it a promising platform for facilitating the copolymerization of ethylene with polar monomers. However, attempts to use dissymmetric *N*,*N*′ ligands with a PYA N-donor and a chelating imine such as pyridine (II, Fig. 1), pyrazole, oxazole, or triazole displayed significant limitation and produced almost exclusively butenes *via* ethylene dimerization rather than polymerization.^14,15^ While this product selectivity was initially attributed to fast β-hydrogen elimination, recent mechanistic investigations indicated a step-growth mechanism rather than a chain-growth, implying re-coordination of butene as the critical step that controls further conversion of dimers to oligomers and polymers.^16,17^ These mechanistic insights also suggest a key role of the ancillary ligand and the counterion,^13^ and suggest optimization of the steric and electronic properties of the chelating N-ligand as a key methodology for catalyst improvement.

Here, we introduce a novel class of palladium complexes supported by strongly donating and sterically versatile bisPYA ligands III (Fig. 1). Deliberate substitution of the *ortho*-positions of the PYA scaffold increases steric shielding, which plays an important role in improving the reactivity and selectivity of the catalyst. These ligands are sterically similar to state-of-the art Brookhart's α-diimine ligands^18–23^ while having significantly altered electronic properties. Specifically, the introduction of two strongly electron-donating PYA groups within one ligand generates an electron-rich metal center, which is expected to be beneficial for higher polymerization productivity.^24^ Here, we demonstrate that appropriately tailored bisPYA ligands induce high catalytic activity and effectively promote polymerization even under very mild conditions.

## Results and discussion

### Synthesis of the complexes

**Scheme 1:**
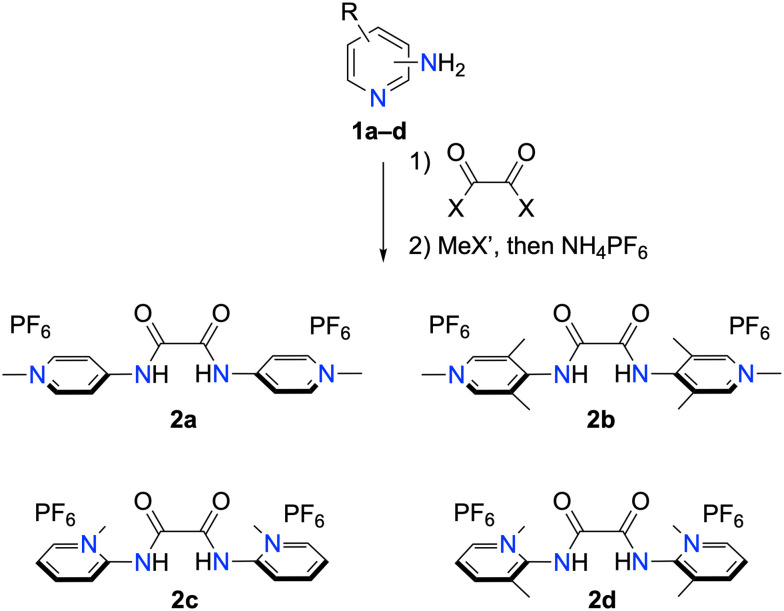
Two-step synthesis of bis-PYA ligands 2a–2d. Conditions: For 2a and 2c: 1) diethyl oxalate, neat, 2) MeOTf in CH_2_Cl_2_; for 2b: 1) oxalyl chloride, NEt_3_ in CH_2_Cl_2_, 2) MeI in CH_2_Cl_2_; for 2d 1) oxalyl chloride, NEt_3_ in CH_2_Cl_2_, 2) MeOTf in CH_2_Cl_2_.

The synthesis of the bisPYA ligands started from the appropriately substituted aminopyridine 1a–d (Scheme 1). Condensation with either diethyloxalate or oxalyl chloride yielded the corresponding bis(amides), and subsequent pyridine *N*-methylation followed by anion exchange with NH_4_PF_6_ afforded the bisPYA ligand precursors 2a–d as PF_6_ salts. Complete methylation was evidenced by a characteristic downfield shift of the pyridinium proton resonances. For example, the pyridinium H^6^ resonance shifted from *δ*_H_ = 8.32 in the bis(amide) to 8.64 in 2d.

**Fig. 2:**
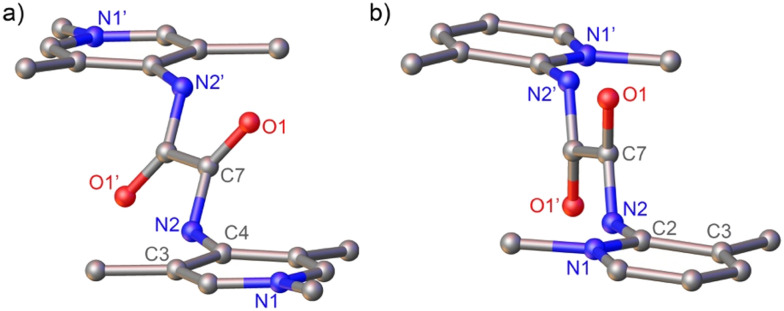
Crystallographically determined molecular structures of a) 2b and b) 2d (all hydrogen atoms and anions omitted for clarity). Selected metrics for 2b: N2–C4 1.414(3) Å; C7–N2–C4–C3 58.8(3)°; for 2d: N2–C2 1.408(2) Å, C7–N2–C2–N1 84.3(5)°.

Crystals suitable for X-ray diffraction analysis were grown for 2b and 2d by slow diffusion of Et_2_O into a MeCN solution of the bisPYA salts. These compounds were of interest as they feature identical steric environments around the amide site, yet differ in the position of the pyridine nitrogen. Analysis of the molecular structure of the *para*-PYA system 2b revealed an ∼60° torsion angle between the amide and the pyridinium heterocycle (Fig. 2). In contrast, the heterocycles in the *ortho*-PYA salt 2d are quasi orthogonal to the amide unit with an 84.3(5)° torsion angle. This trend is in line with the smaller torsion angles in the *para*-PYA salt 2a compared to the *ortho*-PYA analogue 2c (6.94° *vs.* 40.46°),^25^ though in this latter comparison, the different *ortho*-substitution pattern may play a significant role.^15,26^ In contrast, a comparison of the smaller torsion angle in 2b*vs.* large angles in 2d suggests that the *ortho vs. para*-PYA substitution influences the arrangement of the heterocycle with respect to the amide unit independent of steric components.

**Scheme 2:**
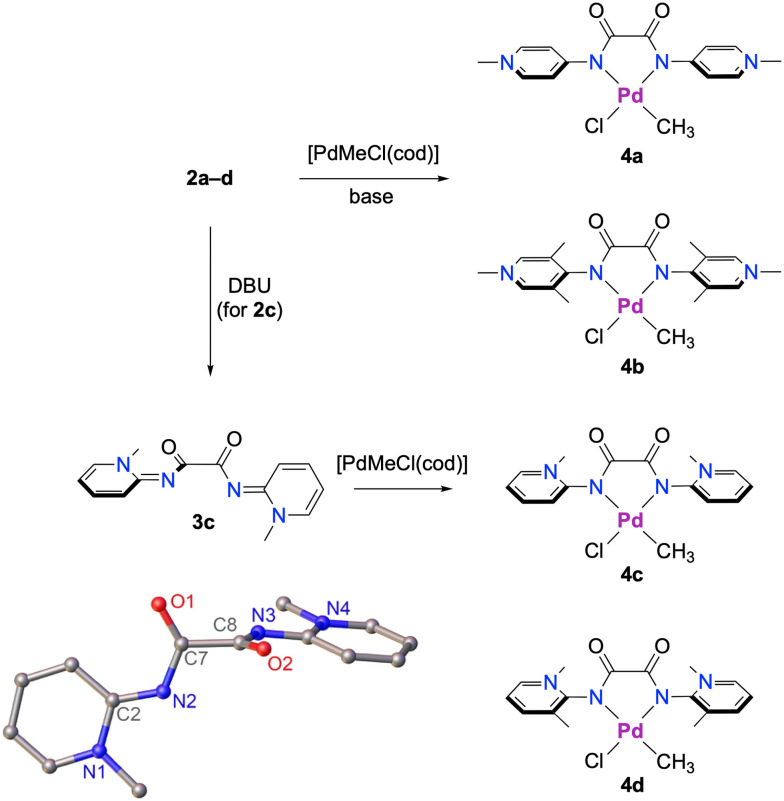
Synthesis of Pd complexes 4a–4d with PYA ligand sites represented in their zwitterionic (pyridinium amidate) resonance form, though there is also contribution from a neutral pyridylidene imine resonance form to be considered. Bottom left: crystallographically determined molecular structure of 3c (50% probability ellipsoids, hydrogen atoms omitted for clarity). Selected metrics for 3c: N2–C2 1.338(14) Å; C7–N2–C2–N1 4.01(9)°.

The bis(PYA) salt 2c readily formed the deprotonated free bis(PYA) ligand 3c in the presence of DBU. Double deprotonation and formation of 3c was evidenced by a substantial upfield shift of all the pyridinium proton signals (Δ*δ*_H_ up to 1.2 ppm). Similarly, the NCH_3_ resonance shifted from *δ* = 4.20 to 3.72 ppm. In contrast to 2c, the other bis(PYA) salts failed to give clean reactions. Attempts to deprotonate these salts with bases such as K_2_CO_3_, DBU, or LiHMDS all failed (Scheme 2). While characteristic resonances of the deprotonated bis(PYA) were observed *in situ*, *e.g.* treatment of 2d with 2.5 eq. DBU resulted in a diagnostic^27^ upfield shift of the NCH_3_ resonance from *δ*_H_ = 4.26 to 3.70, isolation of this species by sequential THF washing did not lead to any clean material.^28,29^ With a stronger base like LiHMDS, an unknown species was isolated, tentatively assigned to the Li adduct (Fig. S17 and S18).^30^

Single crystal structure determination of 3c revealed some degree of double-bond localization within the pyridine ring, *e.g. C*_pyr3_–*C*_pyr4_ decreased from 1.380(2) Å in 2c^25^ to 1.3653(19) Å in 3c, and the exocyclic *C*_PYA_–*N*_PYA_ bond shortened from 1.3924(19) Å in 2c to 1.338(14) Å. Moreover, the amide unit and the pyridinium heterocycle are essentially co-planar with a torsion angle *C*_CO_–*N*_PYA_–*C*_PYA_–*N*_pyr_ = 4.01(9)° *vs.* 40.46(15)° in the salt precursor. However, the two amide units lost co-planarity and are essentially orthogonal in 3c with *N*_PYA_–*C*_CO_–*C*_CO_′–*N*_PYA_′ = 89.40(11)° (*cf.* 0.0(3)° in 2c). This arrangement also led to a close 2.23(1) Å contact between H3 of the pyridinium ring and the adjacent carbonyl oxygen.

Direct palladation of the bis(PYA) salts 2a–d without isolation of the deprotonated ligand was accomplished with [PdMeCl(cod)] in the presence of 1,8-bis(dimethylamino)naphthalene (proton sponge) as a base and afforded complexes 4a–d in 70–89% yield. Alternatively, neutral bis(PYA) 3c was metalated with [PdMeCl(cod)] in the absence of a base. All four complexes were isolated as air-stable yellow solids. Complex formation was indicated by the high-field singlet of the Pd–CH_3_ resonance in the −0.39 to −0.64 range. These frequencies suggest considerable shielding of the methyl group in complexes 4b–d when compared with related olefin polymerization precatalysts supported by α-diimine (*δ*_H_ = +0.51) or C,N-bidentate pyridyl-NHC ligands (*δ*_H_ = +0.01).^17,31–34^ Only catalyst precursors with anionic C,O-bidentate phenolate-NHC ligands feature a higher field Pd–CH_3_ signal (*δ*_H_ = −0.81),^35^ pointing to a strongly electron-donating bonding mode of the bisPYA ligand (Table S1) and thus a large contribution of zwitterionic resonance structures in 4b–d. Notably, the Pd–CH_3_ resonance of complex 4a appears at a lower field (*δ*_H_ = +0.13). The bis(PYA) ligand in this complex lacks *ortho*-substituents with respect to the amide group, which facilitates a coplanar arrangement of the amide and the pyridinium heterocycle and thus a quinoidal character of the ligand with neutral π-acidic rather than anionic π-basic N-donor sites.^36^

Characterization of complexes 4b and 4d by ^1^H NMR spectroscopy revealed a mixture of two species at RT in DMSO, attributed to a mixture of the parent (methyl)(chloride) complex and its solvento analogue [PdMe(DMSO)(bisPYA)]Cl in a 1 : 2 and 4 : 1 ratio, respectively. In support of this assignment, addition of NBu_4_Cl increased the ratio of the chlorido complex 4b, while addition of AgPF_6_ afforded the solvento complex, together with some complex degradation products (Fig. S30 and S36). The higher solvento ratio with 4b compared to 4d suggests a higher *trans*-influence and stronger *σ* donor properties of the *para*-PYA in 4b than the *ortho*-PYA for quasi-isosteric ligands. This conclusion is also supported by the slightly higher field Pd–CH_3_ resonance in 4b compared to 4d (*δ*_H_ = −0.64 *vs.* –0.58).^37–39^

Complexes 4a–d display only limited stability in dry DMSO-*d*_6_ and new and sharper resonances appeared over time, presumably due to the hygroscopic nature of DMSO. Indeed, dissolving complex 4a in D_2_O resulted in the immediate appearance of a new species, together with a resonance at 0.2 ppm that is diagnostic for methane formation.^40^ After 90 min, no residual complex 4a was detectable anymore, and only the new species was present (Fig. S25). Macroscopically, formation of a palladium mirror was observed, and pressure build-up was noted when opening the NMR tube. These results indicate protonolysis of the Pd–CH_3_ unit,^41–43^ which is known to occur even with weak acids,^44^ and subsequent ligand rearrangement to form [Pd(bisPYA)_2_]Cl_2_.^17^ The homoleptic complex [Pd(bisPYA)_2_]Cl_2_ was identified by the symmetrization of the ligand resonances in the NMR spectra, and by HRMS (*m*/*z* = 681.0948, calcd for [M–Cl]^+^ 681.0957, Fig. S25). Similar reactivity was noted for complexes 4c and 4d, though protonolysis was much slower with these complexes and only 5% degradation was observed after 8 h.^45^

### Reactivity towards ethylene

In order to probe ethylene conversion, complexes 4a–d were activated *in situ* with NaBArF, an established additive for enhancing catalytic activity in olefin oligomerization.^17,46–50^ The *in situ* activation furthermore avoids the introduction of an ancillary ligand such as MeCN that may interfere with olefin bonding and conversion.^16,51^ To assess the potential activity of the palladium complexes, a solution of bisPYA complexes 4a–d and 1.1 eq. NaBArF in CD_2_Cl_2_ was saturated with ethylene (1 bar, *ca.* 1 M), and olefin conversion was monitored by ^1^H NMR spectroscopy (Table 1, Fig. S53–S59). Under these conditions, complex 4a produced predominantly butenes in the first 45 min, and this composition did not change upon extending the reaction time to 16 h (Table 1, entry 1, Fig. S58). This low activity towards olefin oligomerization may, in part, be attributed to the low solubility of 4a. In an attempt to improve the solubility, complex 4a′ with *N*-Bu rather than *N*-Me substituents was prepared (Fig. S26). Despite the longer aliphatic substituents, the solubility of 4a′ was not significantly improved and catalytic activity was identical to that of 4a (entry 2). When the *para*-PYA was sterically rigidified by two methyl substituents as in complex 4b, the activity increased and oligomers were formed upon extended periods of time, indicated by the 15 : 1 alkyl/vinyl proton ratio after 16 h (entry 3, Fig. S53–S55).

**Table 1:** Reactivity of bisPYA complexes 4a–d with ethylene^a^

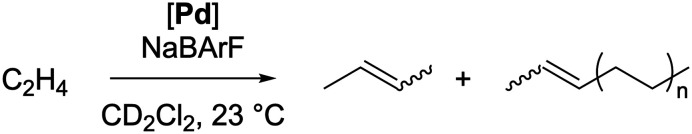									
Entry	[Pd]	NaBArF	Conversion^b^		Major product	Alkyl/vinyl ratio^c^		l/b ratio^c^	
			45 min	16 h		45 min	16 h	45 min	16 h
1	4a	1.1 eq.	>99%	>99%	Butenes	4/1	4/1	—	—
2	4a′	1.1 eq.	>99%	>99%	Butenes	4/1	4/1	—	—
3	4b	1.1 eq.	>99%	>99%	Oligomers	6/1	15/1	3/1	5/2
4	4c	1.1 eq.	85%	93%	Butenes	3/1	5/1	—	—
5	4d	1.1 eq.	>99%	>99%	Oligomers	14/1	31/1	20/1	4/3
6	4a	2.5 eq.	>99%	>99%	Butenes	4/1	6/1	20/1	7/1
7	4b	2.5 eq.	>99%	>99%	Polymer	17/1	150/1	99/1	1/4
8	4c	2.5 eq.	>99%	>99%	Oligomers	9/1	27/1	10/1	1/2
9	4d	2.5 eq.	>99%	>99%	Polymer	340/1	270/1^d^	5/1	<1/10

a Reaction conditions: [Pd] (10 μmol), NaBArF (11 or 25 μmol), durene (50 μmol, internal standard) in dry CD_2_Cl_2_ (0.5 mL) saturated with ethylene (∼1 M).

b Conversion monitored by ^1^H NMR spectroscopy after 45 min and 16 h under constant spinning at 23 °C, incomplete ethylene conversion was determined by ^1^H NMR integration of the butene and propylene resonances against the residual ethylene signal and is approximate only.

c Alkyl/vinyl and linear/branched (l/b) ratios determined by ^1^H NMR spectroscopy of the crude reaction mixture (Fig. S53–S55).

d Decreasing alkyl/vinyl ratio attributed to the precipitation of polymeric products.

A similar correlation between catalyst structure and activity was observed for the *ortho*-PYA complexes. While the un-substituted *ortho*-PYA complex 4c was only poorly active and did not reach full ethylene conversion, yielding predominantly butenes (entry 4), the methylated version 4d promoted the formation of oligomers already within the first 45 min, and these chains further grew upon extending the reaction time to reach a 31 : 1 alkyl/vinyl ratio after 16 h (entry 5). Also, the branching degree of the oligomers increased considerably from 20 : 1 to 20 : 15 during this time, indicating efficient olefin insertion and β-H elimination processes. GC-MS analysis revealed the presence of fragments as large as C_24_H_48_ (Fig. S64–S76)_._

Higher activity was achieved when the quantity of NaBArF was increased to 2.5 eq. While complex 4a gave only modestly longer oligomers, with butenes remaining the major products (entry 6), 4b resulted in the formation of oligomers already after 45 min (17 : 1 alkyl/vinyl ratio), and gave polymeric material after 16 h, indicated by a high 190 : 1 alkyl/vinyl ratio (entry 6). Again, branching increased drastically during the process. While initially the formed oligomers were essentially linear with a 99 : 1 linear/branched ratio, this ratio switched to 1 : 4 after 16 h (entry 7). The effect is even more pronounced with the *ortho*-PYA systems 4c and 4d. Complex 4c gave oligomeric products (entry 8), whereas the rigid *ortho*-dimethyl PYA yielded polymeric material already after 45 min with a 340 : 1 alkyl/vinyl ratio (entry 9; Fig. S60 and S61). The preference of the catalyst to initially form linear products is shown also for 4d with a 5 : 1 linear/branched ratio, though after 16 h, no linear fragments were observed anymore, suggesting a very high branching degree as required for low-density PE. The high branching is attributed to a very effective chain walking and β-hydrogen elimination processes imparted by the sterically rigid and shielding bisPYA ligand.

Obviously, the mild conditions (1 bar ethylene, room temperature, closed system) used for assessing these preliminary activities introduce a limited availability of ethylene, which also caps the polymer length and the catalyst productivity. Therefore, experiments were carried out by providing a continuous supply of ethylene (1 bar) into the reaction mixture. Under these conditions, only complexes 4b and 4d yielded polymeric products. The productivity of 4b was rather poor and reached 1.4 g PE per g Pd, presumably due to fast catalyst decomposition as indicated by the detection of some signals in the NMR spectrum that suggest a dissociated bis(PYA) ligand. In contrast, complex 4d produced a waxy polymer that was partially soluble in CD_2_Cl_2_. NMR analysis revealed a high branching degree of 111 methyl groups per 1000 methylene units^11^ with a wide variety of branching structures and topologies (Fig. S62 and S63).^52–54^ Under these ambient conditions, 4d reached a productivity of 101.5 g PE per g Pd or 10.8 kg PE per mol Pd. These values are at the lower end when comparing with the Pd(acac)_2_/BF_3_·OEt_2_ system,^55*a*^ or phosphine-sulfonate systems with ferrocene or naphthalene bridges, even though the latter were used at 5–10 bar ethylene pressure.^55*b,c*^ The productivity is also far from the 4000 kg PE per mol Pd productivity of state-of-the-art Brookhart-type palladium catalysts, though those conversions were obtained under harsher conditions (10 bar ethylene, 60 °C)^22,56^ compared to the 1 bar/25 °C conditions applied here for 4d. Therefore, the productivity and selectivity of complex 4d represents a promising improvement towards efficient polymerization catalysts based on PYA ligands, warranting further tests under (co-) polymerization conditions closer to those of industrial settings in terms of temperature, pressure, and possibly co-monomer addition.

### Metal sequestration by the bisPYA ligand

The excess of NaBArF required to induce high catalytic activity suggests interactions with the Pd complex beyond simple halide abstraction and formation of the putative active species [Pd(Me)(ethylene)(bisPYA)]^+^. Based on scattered observations of alkali metal bonding to PYA units, we hypothesized a direct interaction between the Na^+^ cation with the bis(amide) backbone of the bis(PYA) ligand of the complex as established for other 1,2-dicarbonyl ligands.^57,58^ Such a bonding might lower the efficacy of NaBArF in halide abstraction with detrimental consequences for catalytic activity.

**Scheme 3:**
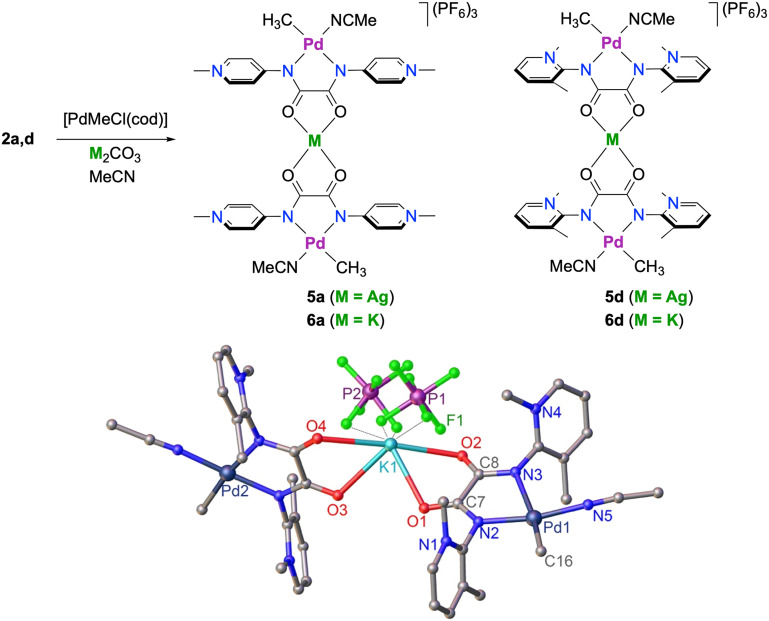
Synthesis of silver-bridged cationic complexes 5a and 5d and potassium-bridged complexes 6a and 6d, and a crystallographically determined molecular structure of 6d (hydrogen atoms and non-coordinating anions omitted for clarity). Selected metrics for 6d: Pd1–C16 2.060(14) Å, Pd1–N2 1.978(5) Å, Pd1–N3 2.055(4) Å, Pd1–N5 2.006(10) Å, C7–O1 1.238(7) Å; average K–O 2.74 Å, average K–F contact 2.97 Å, C7–N2–C2–N1 72.8(7)°; Pd1–N2–C2–N1 82.6(6)°; N2–Pd1–N3 82.79(17)°.

To verify this hypothesis, reactions of complexes 4a–d with different sodium salts (NaBArF, Na_2_CO_3_) were carried out, though no defined products were detectable. As an alternative route, palladation of ligand precursors 2a or 2d in the presence of Na_2_CO_3_ led to incomplete reactions and the formation of mixtures. However, complexation of these ligands with [PdMeCl(cod)] in the presence of Ag_2_CO_3_ resulted in the formation of spectroscopically pure trimetallic complexes 5a and 5d, respectively, which were isolated as air-stable dark yellow solids (Scheme 3). The ^1^H NMR spectra were distinct from those of 4a and 4d, most evidently by the downfield shift of the Pd–CH_3_ resonance, *e.g. δ*_H_ = +0.29 for 5a*vs.* +0.13 for 4a (both DMSO-*d*_6_ solutions). Addition of AgPF_6_ to a MeCN solution of 4d gave a precipitate, which has ^1^H NMR data identical to those of 5d, thus providing an alternative synthetic method. Moreover, similar adduct formation was observed when K_2_CO_3_ was used instead of Ag_2_CO_3_, yielding the corresponding trimetallic Pd⋯K⋯Pd complexes 6a and 6d as air-stable yellow solids.

Adduct formation was confirmed by single-crystal X-ray diffraction of complex 6d. The molecular structure shows a potassium ion connecting two Pd complex units *via* dicarbonyl chelation. The potassium coordination sphere is completed by interaction with fluorine atoms from two distinct PF_6_^−^ anions. These interactions suggest cation stabilization by the bis(PYA) ligand backbone similar to *O*- or *N*-polydentate systems such as crown ethers and cryptands.^59,60^ The average K–O distance in 6d is 2.74 Å, in line with the 2.72 Å reported as the mean value for such interactions.^57,58,61^ The K–F contact distances range from 2.815(16) to 3.05(2) Å, at the longer edge of the typically observed 2.84 Å length.^62–66^

The Pd centers adopt the typical square planar geometry, and the PYA rings are nearly orthogonal to the coordination plane (Pd–*N*_PYA_–*C*_PYA_–*N*_pyr_ = 82.6(6)°), indicative of strongly zwitterionic PYA character. Structural comparisons with the precursor salt 2d reveal only minimal variation in the exocyclic *N*_PYA_–*C*_PYA_ bond lengths (1.416(8) Å in 6d*vs.* 1.408(2) Å in 2d), reinforcing the strongly donating nature of the ligand framework. Notably, the unit cell contains both *syn*- and *anti*-isomers as proposed for complex 4d.^37^

Elemental analysis of bulk 6d suggests a slightly higher sequestration of K^+^ and returned a 1 : 1 ratio of KPF_6_ per Pd(bisPYA) fragment. Similarly, quantitative ^1^H/^19^F NMR integration using 1,2,4,5-tetrafluorobenzene as an internal standard was commensurate with 2 PF_6_^−^ anions per bisPYA ligand (Fig. S1 and S2).^29,67–69^ Although attempts to grow single-crystals for the other adducts were unsuccessful, elemental analysis and quantitative ^19^F NMR spectroscopy provided good support for similar cation bonding in 5a, 5d (1.5 PF_6_^−^ per ligand) and 6a (1.2–2 PF_6_^−^ per ligand; Fig. S1 and S2).

Detailed characterization of these adducts in solution was hampered by the broadness of the ^1^H NMR signals. For 5a, two sets of signals were identified in CD_3_CN solution. Increasing the temperature to 40 °C resulted in sharpening of the signals into well-defined doublets, suggesting dynamic behavior (Fig. S40). DOSY NMR analysis revealed that the two sets of signals belong to species with similar diffusion coefficients, pointing to stereoisomers such as *syn*- and *anti*-rotamers rather than silver association/dissociation equilibria (Fig. S41). The molecular weight extracted from the measured diffusion coefficient (930 g mol^−1^; *D* = 9.3 × 10^−6^ cm^2^ s^−1^) is consistent with the 973 g mol^−1^ calculated for the Ag-containing trication of 5a.^70–72^ Similar solution behavior was noted for adducts 5d, 6a, and 6d. Moreover, mass spectrometry revealed fragments corresponding to the Pd complex unit plus an Ag^+^ and K^+^ cation, respectively (Fig. S42, S45, S49 and S52).

Evaluation of the adducts 5 and 6 in ethylene conversion is convoluted by the presence of the MeCN in the palladium coordination sphere. This ligand has been shown to hinder butene coordination and the formation of polymeric products.^16,51^ Indeed, only butene formation was observed upon exposure of the adducts to ethylene (Table 2, entries 1–4). While complexes 5d and 6d with sterically shielding PYA sites lead to considerable ethylene conversion, the *para*-PYA analogues 5a and 6a were less active and residual ethylene was detected after 1 h. Addition of 1.1 eq. NaBArF to adduct 5d induced some oligomerization, while 2.5 eq. NaBArF as an additive gave longer oligomers as deduced from the high 90 : 1 ratio of alkyl to vinyl protons in the NMR spectrum (entries 5 and 6). Similar effects were observed with the potassium adduct (entries 7 and 8), suggesting that either the BArF anion itself, or the cation exchange from Ag^+^ or K^+^ to Na^+^ plays a significant role in the butene conversion activity of the palladium complexes.

**Table 2:** Reactivity of alkali metal bisPYA Pd adducts 5–6 with ethylene^a^

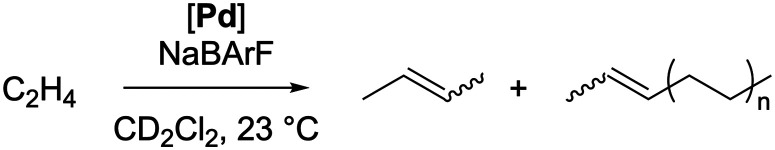									
Entry	[Pd]	NaBArF	Conversion^b^		Major product	Alkyl/vinyl ratio^c^		l/b ratio^c^	
			45 min	16 h		45 min	16 h	45 min	16 h
1	5a	—	84%	>99%	Butenes	2/1	5/1	—	—
2	5d	—	>99%	>99%	Butenes	5/1	7/1	—	8/1
3	6a	—	27%	27%	Butenes	1/5	1/5	—	—
4	6d	—	60%	60%	Butenes	1.5/1	1.5/1	—	—
5	5d	1.1 eq.	>99%	>99%	Oligomers	6/1	13/1	30/1	5/1
6	5d	2.5 eq.	>99%	>99%	Oligomers	23/1	90/1	7/1	5/4
7	6d	1.1 eq.	>99%	>99%	Oligomers	11/1	16/1	20/1	4/1
8	6d	2.5 eq.	>99%	>99%	Oligomers	53/1	50/1^d^	5/1	2/1

a Reaction conditions: [Pd] (10 μmol), NaBArF (0, 11 or 25 μmol), durene (50 μmol, internal standard) in dry CD_2_Cl_2_ (0.5 mL) saturated with ethylene (∼1 M).

b Conversion monitored by ^1^H NMR spectroscopy after 45 min and 16 h under constant spinning at 23 °C, incomplete ethylene conversion was determined by ^1^H NMR integration of the butene and propylene resonances against the residual ethylene signal and is approximate only.

c Ratios determined by ^1^H NMR spectroscopy of the crude reaction mixture (Fig. S53–S55).

d Decreasing alkyl/vinyl ratio attributed to the precipitation of polymeric products.

## Conclusions

This work demonstrates for the first time the efficiency of pyridinium amidate (PYA) ligands in promoting palladium-catalyzed ethylene polymerization, thus expanding the family of *N*,*N*-bidentate ligands for this type of reaction beyond α,α′-diimine and bian-type ligands. Related PYA ligand systems have succeeded in converting ethylene, though they afforded either selectively butenes from dimerization, or low-molecular weight oligomerization products, but never showed activity in polymerization. A critical parameter for entailing polymerization is an orthogonal arrangement of the pyridinium heterocycles with respect to the bis-amidate core of the bisPYA ligands, which was imposed by the introduction of *ortho*-dimethyl substitution of the pyridinium ring. Moreover, electronic tailoring of the ligand by using 2-aminopyridine (*ortho*-PYA) scaffolds and *in situ* catalyst activation with BArF to suppress solvent coordination are key factors for ensuing polymer growth. The sensitivity to solvent coordination underlines the step-growth mechanism of these systems. Under mild conditions (room temperature, 1 bar ethylene), a remarkable productivity was noted with about 10 kg PE per mol palladium, which bodes well for further polymerization tests under industrially relevant conditions (higher temperature and ethylene pressure) as well as for co-polymerization, *e.g.* with polar co-monomers. Time-dependent analysis of the products indicates the initial formation of linear polymers, yet a high degree of branching after prolonged reaction time, indicative of efficient chain walking and β-hydride elimination processes, an attractive property for the production of low-density polyethylene (LDPE). Mechanistic work furthermore provides support for a dual role of NaBArF as (i) an additive for halide abstraction and (ii) as a templating agent *via* coordination to the 1,2-dicarbonyl core of the bis(PYA) ligand. Such metal sequestration may offer opportunities for tailoring the ligand donor properties through remote coordination, an aspect that is currently under further investigation in our laboratories.

## Conflicts of interest

The authors declare no competing financial interest.

## Supplementary Material

CY-015-D5CY01102G-s001

CY-015-D5CY01102G-s002

## Data Availability

All data pertaining to this manuscript are available as electronic supporting information (SI): synthetic procedures, full characterization of the ligand precursors, Pd complexes, NMR analysis and GC-MS characterization of the catalytic mixtures, crystallographic data and catalytic experiments (pdf). Supplementary information is available. See DOI: https://doi.org/10.1039/d5cy01102g. CCDC 2440549–2440552 contain the supplementary crystallographic data for this paper.^73*a*–*d*^
