# Supplementary material for: Tunable bis(pyridinium amidate) ligands efficiently promote palladium-catalyzed ethylene polymerization
Source: Catal Sci Technol. 2025 Oct 30;15(24):7464–72. doi: 10.1039/d5cy01102g (PMC12614071; doi:10.1039/d5cy01102g)
Supplement: CY-015-D5CY01102G-s001 [file CY-015-D5CY01102G-s001.pdf]

Supplementary Information (SI) for Catalysis Science & Technology.
This journal is © The Royal Society of Chemistry 2025

Electronic Supporting Information

belonging to

Tunable bis(pyridinium amidate) ligands efficiently promote palladium-catalyzed ethylene polymerization

Esaïe Reusser[a] and Martin Albrecht*[a]

[a] Department of Chemistry, Biochemistry and Pharmaceutical Sciences, University of Bern, Freiestrasse 3, 3012 Bern, Switzerland

Email: martin.albrecht@unibe.ch

Table of contents

S.1. SYNTHETIC PROCEDURES **3**

S.1.1 General information 3

S.1.2. Synthesis of ligands 5

S.1.2.1 Synthesis of ligand 2a’ 5

S.1.2.2 Synthesis of ligand 2b 6

S.1.2.3 Synthesis of ligand 3c 11

S.1.2.4 Reaction of 2c with LiHMDS 15

S.1.2.5 Synthesis of ligand 2d 18

S.1.3. Synthesis of neutral complexes 21

S.1.3.1 Synthesis of complex 4a 21

S.1.3.2 Synthesis of complex 4a’ 23

S.1.3.3 Synthesis of complex 4b 24

S.1.3.4 Synthesis of complex 4c 26

S.1.3.5 Synthesis of complex 4d 28

S.1.4. Synthesis of cationic complexes 33

S.1.4.1 Synthesis of complex 5a 33

S.1.4.3 Synthesis of complex 5d 36

S.1.4.2 Synthesis of complex 6a 38

S.1.4.4 Synthesis of complex 6d 42

S.2. REACTIVITY WITH OLEFINS **44**

S.3. CHARACTERIZATION OF THE PRODUCTS **45**

S.3. CRYSTAL STRUCTURE DETERMINATIONS **59**

S.4. REFERENCES **63**

S.1. Synthetic procedures

S.1.1 General information

Bis(pyridin-4-yl)oxalamide **1a** and bis(pyridinium)oxalamide **2a** were prepared according to previously reported procedures.[S1] MeCN, CH_2Cl_2 and tetrahydrofuran (THF) were dried by passage through a solvent purification column. CD_2Cl_2 was dried over 4 Å molecular sieves before catalytic runs. DMSO-d_6 was dried by 4 consecutive one-week treatments with molecular sieves and kept in an argon-filled glovebox. All other reagents and solvents were commercially available and used as received. Ethylene was purchased from Sigma-Aldrich in 1 L CANGas pressurized bottle with a purity of 99.95%. Unless otherwise stated all the reactions were run using standard Schlenk techniques under an atmosphere of dry nitrogen and under exclusion of air.

Unless otherwise specified, NMR spectra were recorded at 25 °C on Bruker spectrometers operating at 300 or 400 MHz (^{1}H NMR), 75 or 100 MHz (^{13}C{^{1}H} NMR), and 282 MHz (^{19}F{^{1}H}NMR) respectively. Chemical shifts (δ) are expressed in ppm downfield from $SiMe_4$ using the residual protonated solvent as an internal standard and coupling constants in Hz. Assignments are based on homo- and heteronuclear shift correlation spectroscopy. Quantitative ^{19}F NMR analysis was performed by adding a drop of 1,2,4,5-tetrafluorobenzene to a pure solution of the analyte. Subsequent comparison of the standard to analyte ^{1}H and ^{19}F ratio indicated the number of fluorinated anions per ligand unit. The method was tested against control samples with known number of fluorine nuclei and gave results within classical NMR integration errors. 16 scans and a relaxation delay of 6 s were used to ensure a reliable signal integration.[S2–S4] A representative example is given in Fig. S1 and S2.

High resolution mass spectrometry was carried out with a Thermo Scientific LTQ Orbitrap XL (ESI-TOF) or with a Thermo Scientific Q Exactive GC coupled to a Trace 1310 Gas Chromatograph when electron impact ionization (EI-MS) was required to obtain satisfactory data. All the MS data were collected by the mass spectrometry group in the DCBP. Elemental analyses were performed on a Thermo Scientific Flash 2000 CHNS-O elemental analyzer by the microanalytic laboratory at the DCBP.

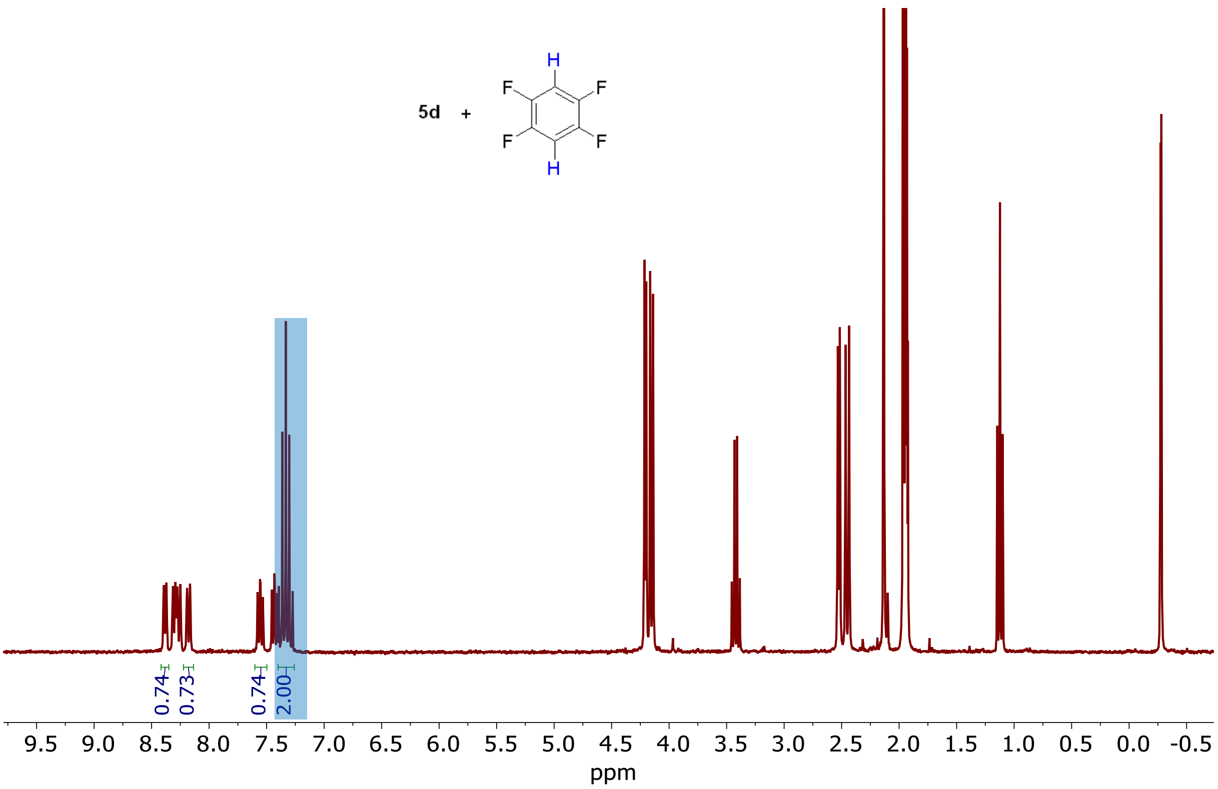

Figure S1: Representative ^{1}H NMR spectrum (CD_3CN, 298 K, 300 MHz) of **5d** after addition of the fluorinated standard 1,2,4,5-fluorobenzene. The resonance associated to the standard is highlighted in blue. In this example there is 0.74 unit of ligand (or Pd complex) per unit of standard.

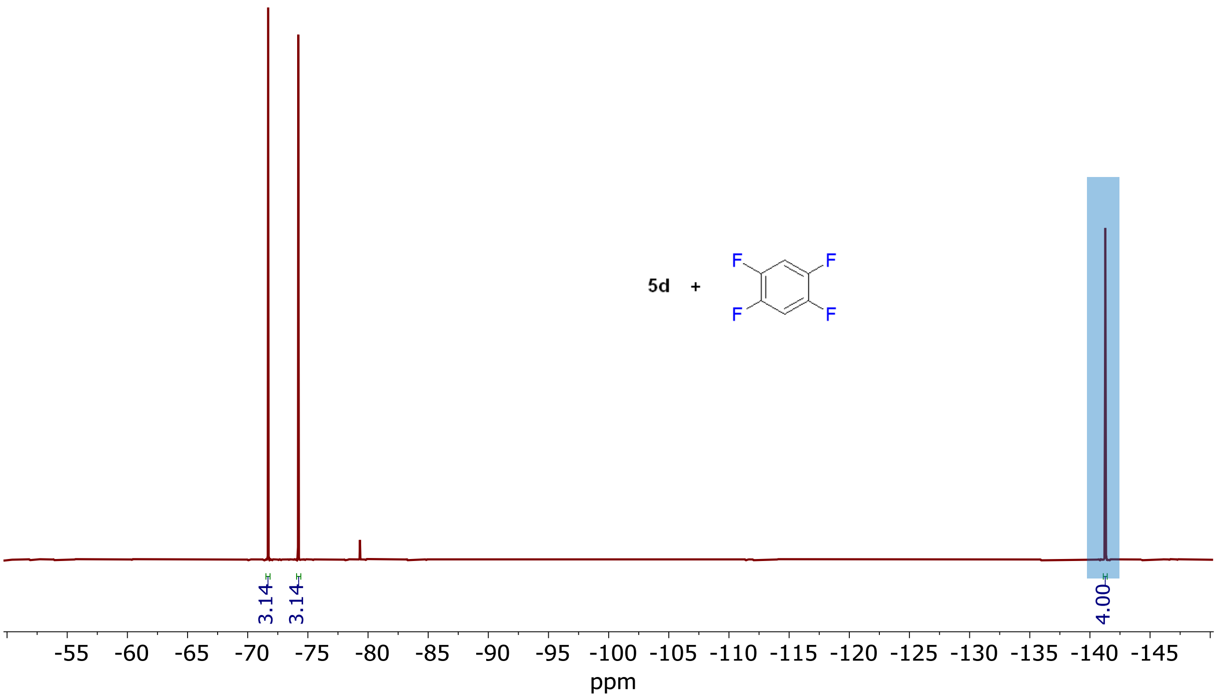

Figure S2: Representative ^{19}F NMR spectrum (CD_3CN, 298 K, 282 MHz) of **5d** after addition of the fluorinated standard 1,2,4,5-fluorobenzene (same sample as used for Fig. S1). The resonance associated to the standard is highlighted in blue. There is (3.14 + 3.14) / 6 = 1.05 unit of PF_6 per unit of standard. The calculated PF_6 to ligand ratio is hence 1.05 / 0.74 = 1.42

S.1.2. Synthesis of ligands

S.1.2.1 Synthesis of ligand 2a'

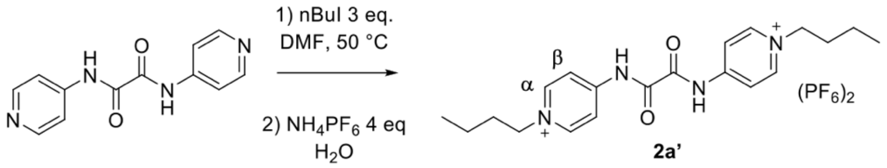

Di(pyridin-4-yl)oxalamide (0.450 g, 1.86 mmol) was suspended in dry DMF (20 mL). An excess of *n*BuI (700 μL, 5.57 mmol) was added and the reaction was stirred at 50 °C for 18 h. After cooling to room temperature, all volatiles were removed, and the resulting solid was dissolved in the minimum amount of H_2O. NH_4PF_6 (1.21 g, 7.44 mmol) was dissolved in the minimum amount of H_2O and both solutions were combined and stirred for 5 min. The resulting solid was collected through filtration, washed with H_2O, Et_2O, and pentane, and dried to yield **2a'** as a pale brown solid (900 mg, 79%). ^{1}H NMR (400 MHz, DMSO-d_6, 298 K): δ = 12.24 (s, 2H, N*H*), 8.93 (d, $^3J_{HH}$ = 7.2 Hz, 4H, C*H*α PYA), 8.42 (d, $^3J_{HH}$ = 7.2 Hz, 4H, C*H*β PYA), 4.48 (t, $^3J_{HH}$ = 7.3 Hz, 4H, N–C*H*$_2$–CH$_2$–CH$_2$–CH$_3$), 1.87 (p, $^3J_{HH}$ = 7.3 Hz, 4H, N–CH$_2$–C*H*$_2$–CH$_2$–CH$_3$), 1.30 (h, $^3J_{HH}$ = 7.3 Hz, 4H, N–CH$_2$–CH$_2$–C*H*$_2$–CH$_3$), 0.92 (t, $^3J_{HH}$ = 7.3 Hz, 4H, N–CH$_2$–CH$_2$–CH$_2$–C*H*$_3$). ^{13}C{^{1}H} NMR (101 MHz, DMSO-d_6, 298 K): δ = 158.97 (*C*O), 150.99 (*C*pyr), 145.46 (*C*Hα PYA), 116.44, (*C*Hβ PYA), 59.32 (N–*C*H$_2$–CH$_2$–CH$_2$–CH$_3$) 32.26 (N–CH$_2$–*C*H$_2$–CH$_2$–CH$_3$), 18.70 (s, N–CH$_2$–CH$_2$–*C*H$_2$–CH$_3$), 13.29 (s, N–CH$_2$–CH$_2$–CH$_2$–*C*H$_3$). ^{19}F{^{1}H} NMR (376 MHz, DMSO-d_6, 298 K): -70.16 (d, $^1J_{FP}$ = 711.4 Hz). ^{31}P{^{1}H} NMR (162 MHz, DMSO-d_6, 298 K): -144.20 (septet, $^1J_{PF}$ = 711.4 Hz). HR-MS (m/z): calculated for $[M–PF_6]^+$ = 501.1849, found 501.1841.

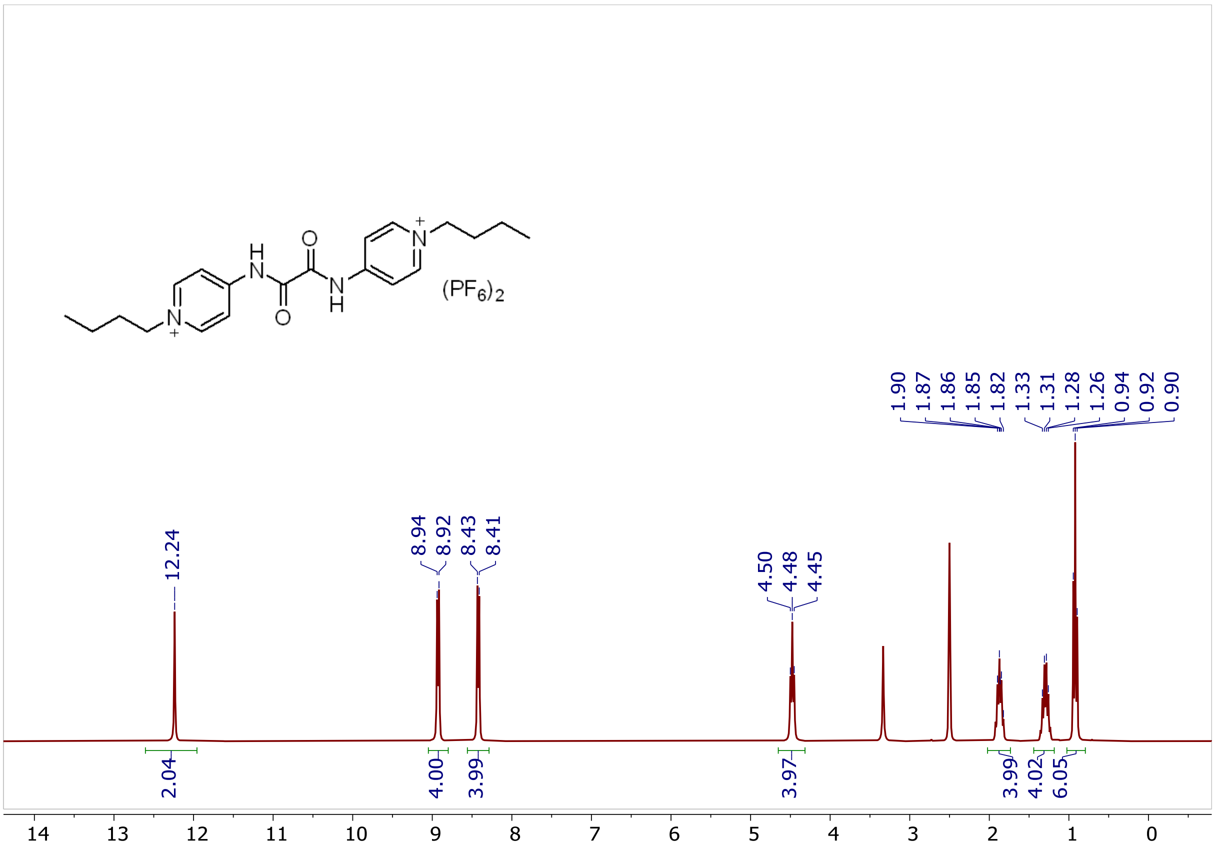

Figure S3: ^{1}H NMR spectrum (DMSO-d_6, 298 K, 300 MHz) of **2a'**

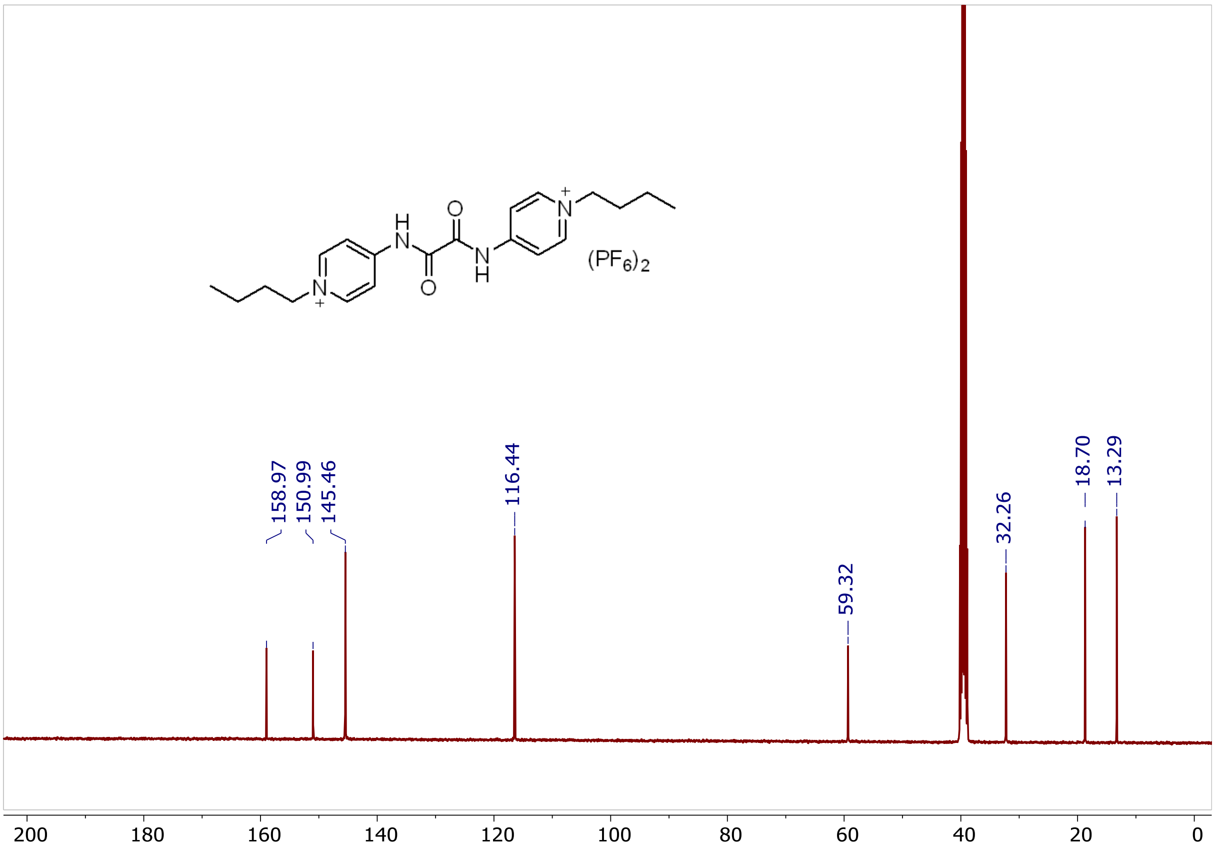

Figure S4: $^{13}C\{^{1}H\}$ NMR spectrum (DMSO-d_6, 298 K, 101 MHz) of **2a'**

S.1.2.2 Synthesis of ligand 2b

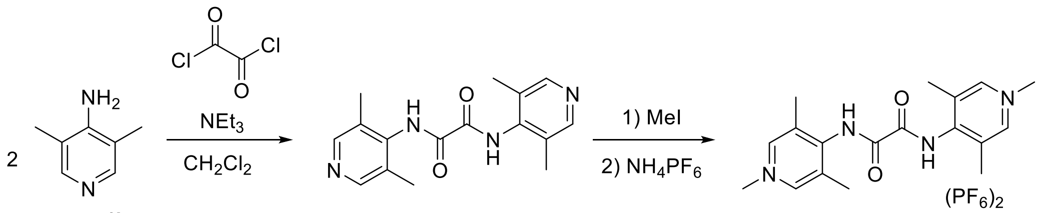

To a solution of 3,5-dimethylpyridin-4-amine **1b** (400 mg, 3.28 mmol) and NEt_3 (470 µL, 3.40 mmol) in CH_2Cl_2 (15 mL) under N_2 at 0 °C was added oxalyl chloride (4.92 mmol, 420 µL) dropwise. The resulting solution was let to stir for 16 h and allowed to slowly warm up to 25 °C. The resulting suspension was filtered, washed with more CH_2Cl_2 (2x 10mL) and purified by gradient column chromatography on alumina gel (0 to 20% MeOH in CH_2Cl_2; 1st frac; rf 0.8 in 30% MeOH/ CH_2Cl_2) to afford the bispyridyl oxalamide as a white solid (134 mg, 28%). ^{1}H NMR (300 MHz, DMSO-d_6, 298K) δ = 10.70 (s, 2H, N*H*) 8.34 (s, 4H, C*H*), 2.19 (s, 12H, CC*H*$_3$) ppm. $^{13}C\{^{1}H\}$ NMR (75 MHz, DMSO-d_6, 298K) δ = 158.05 (*C*O), 148.95 (*C*H), 141.82 (C_{pyr}), 130.00 (*C*CH$_3$), 14.77 (C*C*H$_3$) ppm. HR-ESI-MS (m/z): calculated for $C_{16}H_{19}N_4O_2$ $[M+H]^+$ = 299.1508; found: 299.1506.

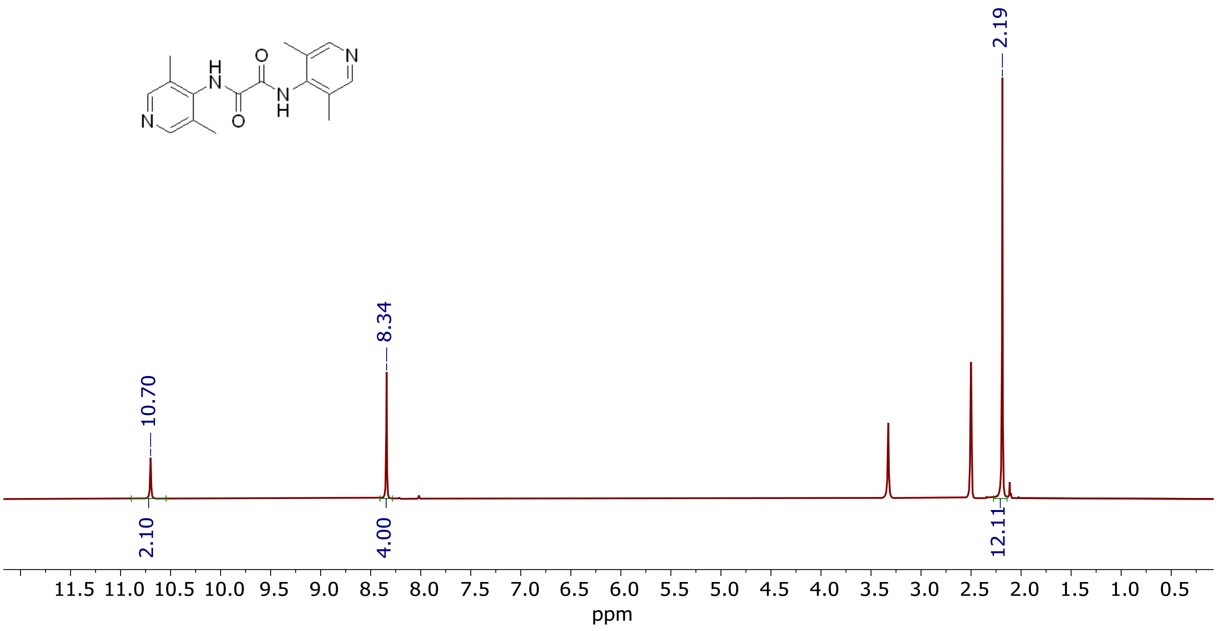

Figure S5: $^{13}C\{^{1}H\}$ NMR spectrum (DMSO-d_6, 298 K, 75 MHz) of bis(3,5-dimethyl-pyridin-4-yl)oxalamide.

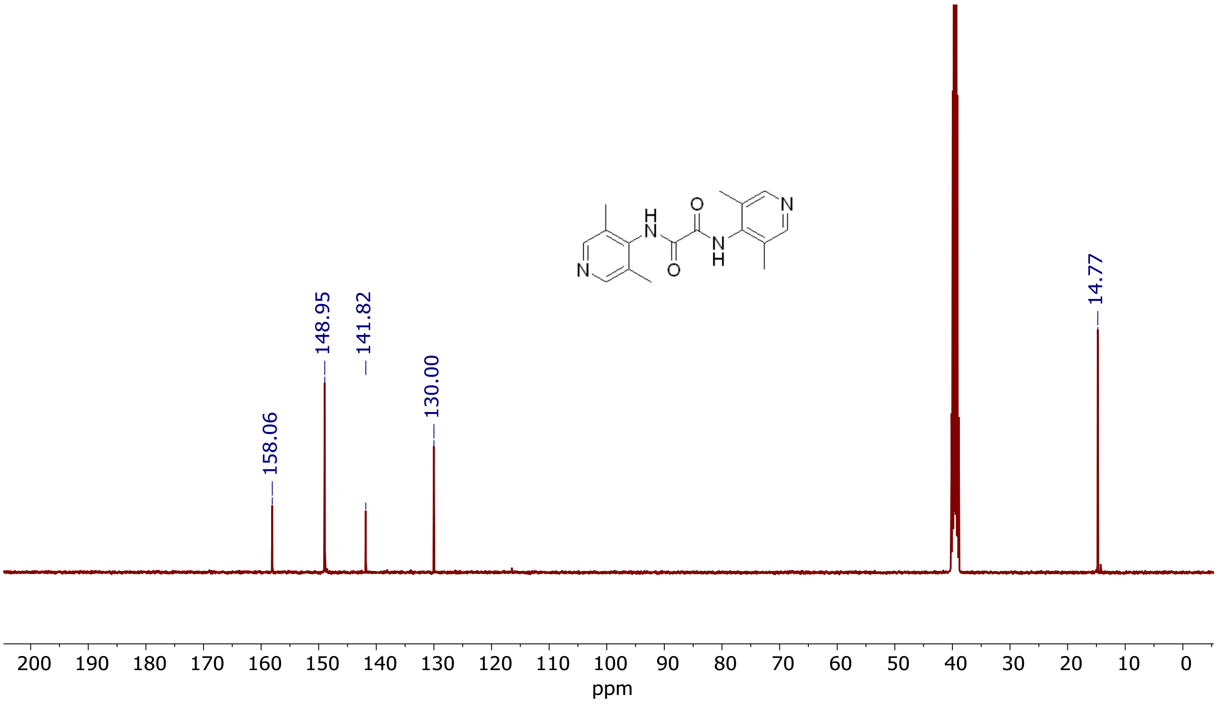

Figure S6: $^{13}C\{^{1}H\}$ NMR spectrum (DMSO-d_6, 298 K, 75 MHz) of bis(3,5-dimethyl-pyridin-4-yl)oxalamide.

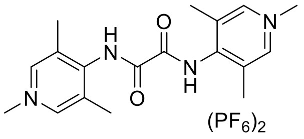

2b

The oxalamide (100 mg, 335 µmol) was suspended in CH_2Cl_2 (5 mL) under N_2, then methyl iodide (85 µL, 1.35 mmol) was added and the resulting suspension was stirred for 16 h at 65 °C. The reaction mixture was then filtered, the cake was washed with more CH_2Cl_2 (2 x 20 mL) and dried under vacuo to yield a white solid. The iodide salt was dissolved into as little water as possible, and the resulting clear solution was merged with a previously prepared solution of NH_4PF_6 (220 mg, 1.35 mmol) in as little water as possible. The formed thick slurry was stirred for 20 min, filtered, washed with cold water and dried under vacuo to yield **2b** as a pale brown solid (147 mg, 71%). Crystals suitable for XRD were grown by slow diffusion of Et_2O in a MeCN **2b** solution. ^1H NMR (300 MHz, CD_3CN, 298K) δ = 9.65 (s, 2H, N*H*) 8.45 (s, 4H, C*H*), 4.22 (s, 6H, NC*H*3), 2.42 (s, 12H, CC*H*3) ppm. $^{13}C\{^1H\}$ NMR (75 MHz, CD_3CN, 298K) δ = 157.61 (*C*O), 149.76 (*C*pyr), 145.26 (*C*H), 136.33 (*C*CH3), 48.56 (N*C*H3) 16.33 (C*C*H3) ppm. $^{19}F\{^1H\}$ NMR (282 MHz, CD_3CN, 298 K): -72.86 (d, $^1J_{FP}$ = 706.6 Hz). HR-ESI-MS (m/z): calculated for $C_{18}H_{14}F_6N_4O_2P$ $[M-PF_6]^+$ = 473.1536; found: 473.1526.

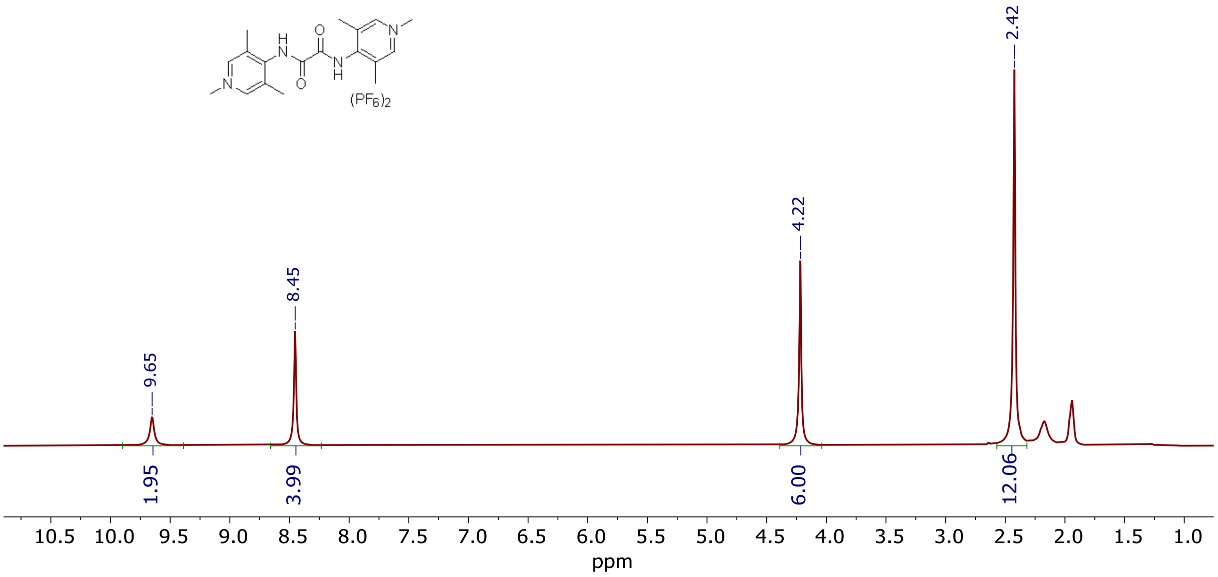

Figure S7: ^1H NMR spectrum (CD_3CN, 298 K, 300 MHz) of **2b**

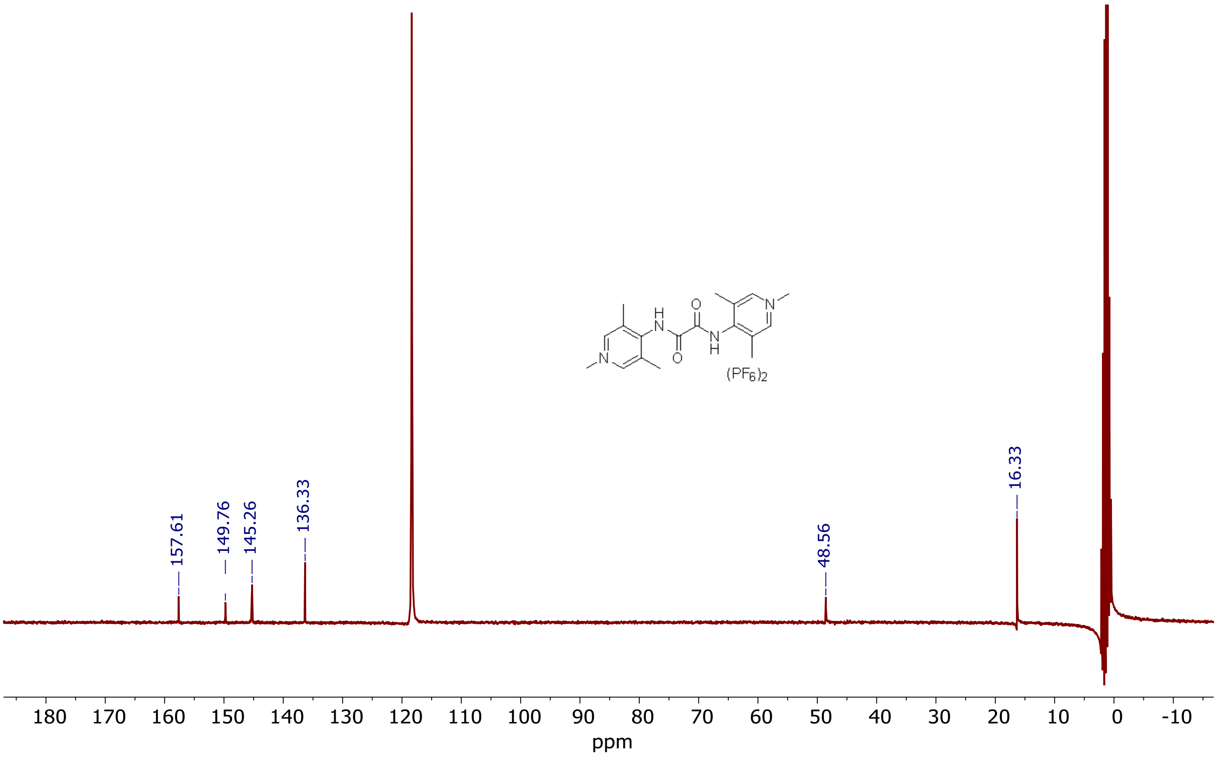

Figure S8: $^{13}C\{^{1}H\}$ NMR spectrum (CD_3CN, 298 K, 75 MHz) of **2b**

Alternatively, compound **2b** was prepared via the protonated pyridinium salt (see Scheme below). This method has the benefit of not requiring a chromatographic purification.

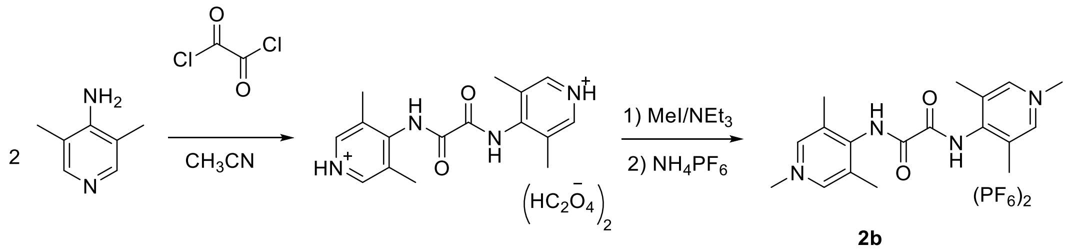

To a solution of 3,5-dimethylpyridin-4-amine (400 mg, 3.28 mmol) in CH_3CN (15 mL) in a vial under N_2 equipped with a pressure outlet connected to a saturated NaOH solution was carefully added oxalyl chloride (13.1 mmol, 1.12 mL) dropwise. After the end of the addition the reaction was let to run for 1 h under a positive nitrogen flow. The outlet was then removed, and the reaction was further stirred at 23 °C for 16 h. The resulting yellow suspension was filtered, washed with Et_2O (2 x 10mL) and dried for 16 h at 90 °C to yield the bis(pyridinium) salt as a pale yellow solid (490 mg, 63%). ^{1}H NMR (300 MHz, DMSO-d_6, 298K) δ = 15.39 (br s, 4H, acidic protons), 11.15 (s, 2H, N*H*), 8.79 (s, 4H, C*H*), 2.33 (s, 12H, CC*H*$_3$) ppm. $^{13}C\{^{1}H\}$ NMR (75 MHz, DMSO-d_6, 298K) δ = 160.82 (*C*O ligand or anion), 156.64 (*C*O ligand or anion), 150.54 (C_{pyr}), 140.15 (*C*H), 134.56 (*C*CH$_3$), 15.33 (C*C*H$_3$) ppm. HR-ESI-MS (m/z): calculated for $C_{18}H_{21}N_4O_6$ $[M-(HC_2O_4)^-]^+$ = 389.1456; found: 389.1451.

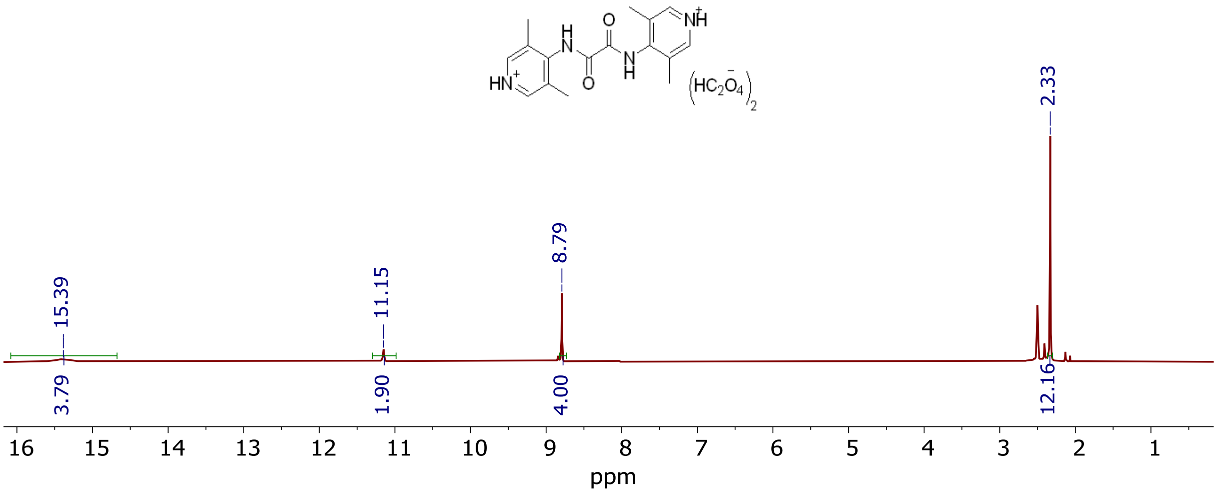

Figure S9: ^{1}H NMR spectrum (DMSO-d_6, 298 K, 300 MHz) of bis(3,5-dimethyl-pyridinium-4-yl)oxalamide.

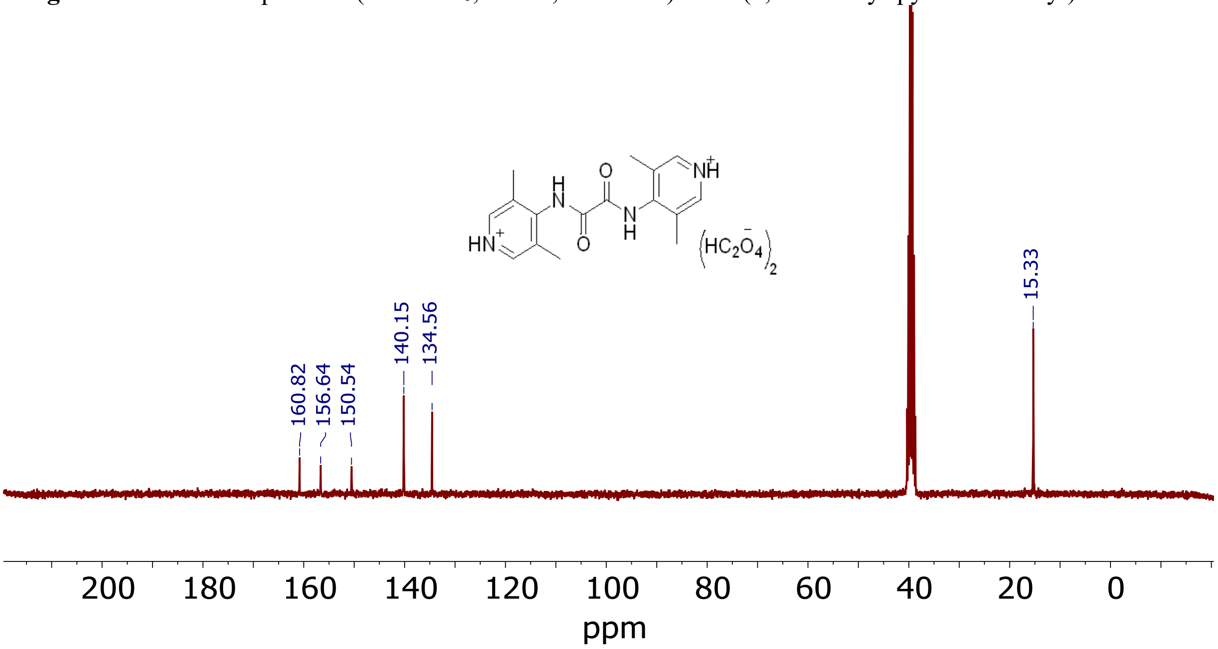

Figure S10: $^{13}C\{^1H\}$ NMR spectrum (DMSO-d_6, 298 K, 75 MHz) of bis(3,5-dimethyl-pyridinium-4-yl) oxalamide.

The bis(pyridinium)oxalamide (100 mg, 209 µmol) was suspended in CH_2Cl_2 (6 mL) under N_2, then methyl iodide (100 µL, 1.60 mmol) was added and the resulting suspension was stirred for 16 h at 65 °C. The reaction mixture was then filtered, the cake was washed with more CH_2Cl_2 (2 x 20 mL) and dried under vacuo to yield a brown solid. The iodide salt was dissolved into as little water as possible, and the resulting clear solution was merged with a previously prepared solution of NH_4PF_6 (200 mg, 1.25 mmol) in as little water as possible. The formed thick slurry was stirred for 20 min, filtered, washed with cold water (3 mL) and Et_2O (2 x 5 mL) and dried for 16 h at 90 °C to yield **2b** as a pale brown solid (25 mg, 20%). Analytical data matched the product obtained via the first synthetic route.

S.1.2.3 Synthesis of the free bis(PYA) 3c

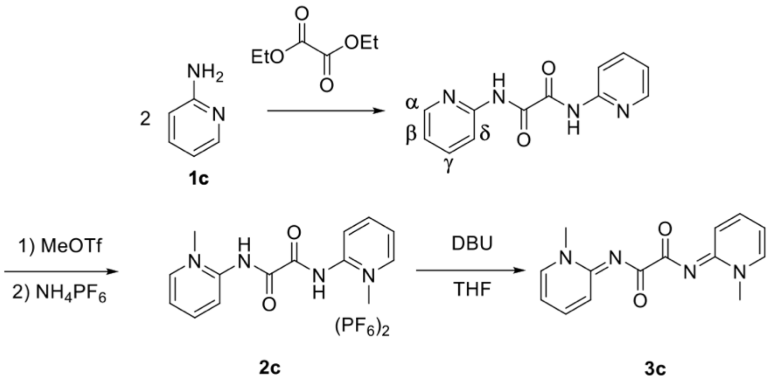

According to a literature procedure,[S5] 2-aminopyridine (18.8 g, 200 mmol) and diethyl oxalate (13.6 mL, 100 mmol) were stirred at 150 °C for 1 h. After cooling to room temperature, the solid was filtered, washed three times with acetone (3x 20mL) and dried to afford compound the bis(pyridyl)oxalamide as a white solid (15.6 g, 65%). ^{1}H NMR (300 MHz, DMSO-d_6, 298 K): δ = 10.48 (s, 2H, N*H*), 8.43 (d, $^3J_{HH}$ = 7.1 Hz, 2H, δC*H*), 8.09 (d, $^3J_{HH}$ = 8.2 Hz, 2H, αC*H*), 7.92 (td, $^3J_{HH}$ = 7.1, $^4J_{HH}$ = 0.7 Hz, 2H, βC*H*), 7.25 (td, $^3J_{HH}$ = 8.2 Hz, $^4J_{HH}$= 0.7 Hz, 2H, γC*H*) ppm. ^{13}C{^{1}H} NMR (100 MHz, DMSO-d_6, 298 K): δ = 159.0 (*C*O), 150.6 (C_{pyr}), 149.0 (δ*C*H), 139.1 (β*C*H), 121.4 (γ*C*H), 114.8 (α*C*H) ppm. MS (m/z): calcd. for $[M+H]^+$ = 243.09; found: 243.09. HR-MS (m/z): calculated for $[M+H]^+$ = 243.0875; found: 243.0877. Elemental analysis calcd. for $C_{12}H_{10}N_4O_2$: C 59.50, H 4.16, N 23.10; found: C 59.18, H 4.19, N 22.76.

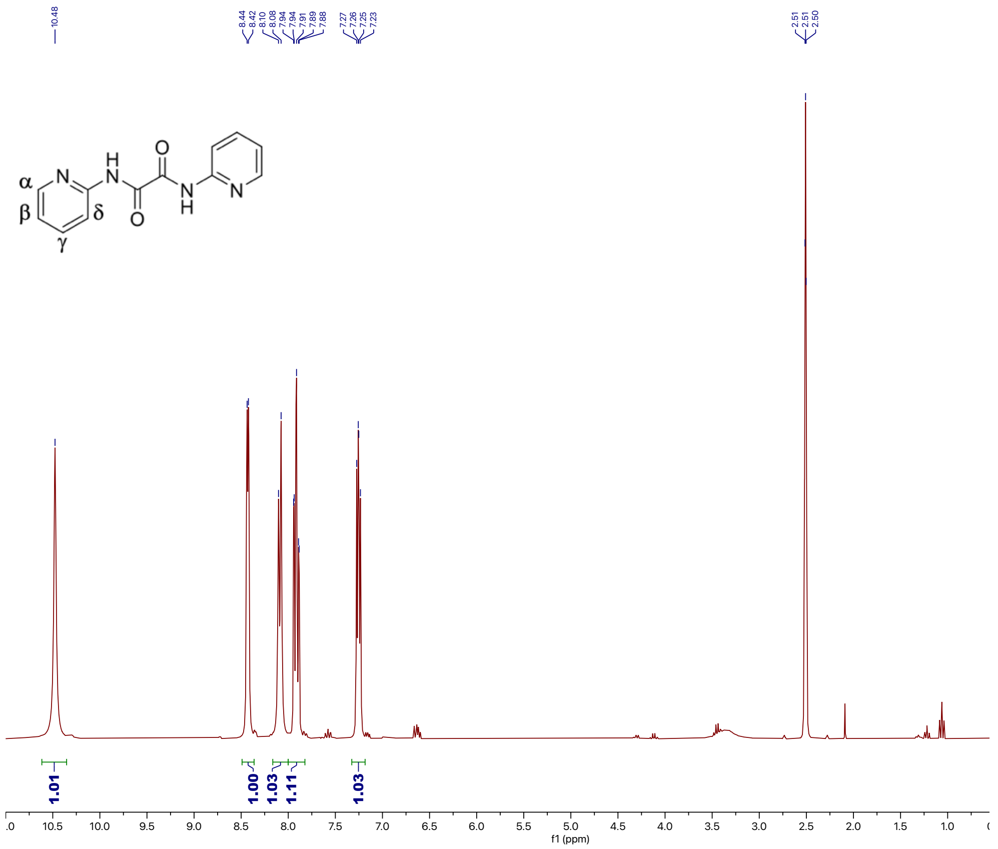

Figure S11: ^{1}H NMR spectrum (DMSO-d_6, 298 K, 300 MHz) of the bis(pyridin-2-yl)oxalamide.

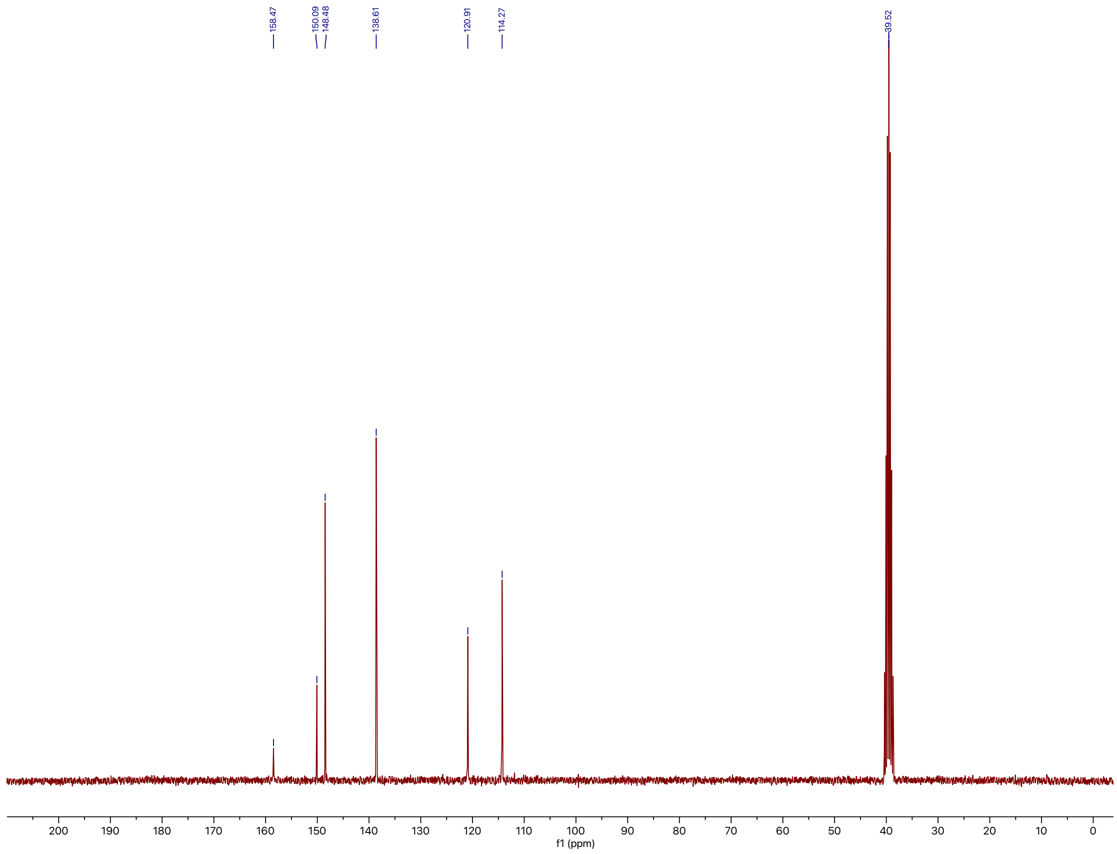

Figure S12: $^{13}C\{^1H\}$ NMR spectrum (DMSO-d_6, 298 K, 300 MHz) of the bis(pyridine-2-yl)oxalamide.

According to a literature procedure,[S5] bis(pyridin-2-yl)oxalylamide (2.0 g, 8.3 mmol) and MeOTf (2.1 mL, 18 mmol) were dissolved in CH_2Cl_2 (80 mL) and stirred for 18 h at room temperature. The formed white solid was filtered, washed with Et_2O and dried. It was subsequently dissolved in as little water as possible and mixed with a separately prepared solution NH_4PF_6 (6.7 g, 41 mmol) dissolved in as little water as possible. After joining the solutions, the reaction was stirred at room temperature for 15 min and the formed white solid was filtered, washed with H_2O and dried to afford salt **2c** as a white solid (3.5 g, 78%). ^1H NMR (300 MHz, DMSO-d_6, 298 K): δ = 9.75 (br s, 2H, N*H*), 8.93 (d, $^3J_{HH}$ = 6.2 Hz, 2H, αC*H*), 8.57 (t, $^3J_{HH}$ = 8.1 Hz, 2H, γCH_{pyr}), 8.26 (d, $^3J_{HH}$ = 8.1 Hz, 2H, δCH_{pyr}), 7.86 (t, $^3J_{HH}$ = 6.2 Hz, 2H, βCH_{pyr}), 4.28 (s, 6H, CH_3) ppm. $^{13}C\{^1H\}$ NMR (75 MHz, DMSO-d_6, 298 K): δ = 159.24 (*C*O), 148.10 (C_{pyr}), 146.15 (γ*C*H), 145.72 (α*C*H), 123.59 (δ*C*H), 123.11 (β*C*H), 44.08 (CH_3) ppm. $^{19}F\{^1H\}$ NMR (282 MHz, DMSO-d_6, 298 K): -70.18 (d, $^1J_{FP}$ = 711.4 Hz). $^{31}P\{^1H\}$ NMR (121 MHz, DMSO-d_6, 298 K): -144.35 (septet, $^1J_{PF}$ = 711.4 Hz). HR-MS: m/z calcd. for $C_{14}H_{16}F_6N_4O_2P$ $[M-PF_6]^+$ = 417.0904; found: 417.0910. Elemental analysis calcd. for $C_{14}H_{16}F_{12}N_4O_2P_2$: C 29.91, H 2.87, N 9.97; found: C 30.12; H 2.83; N 9.65.

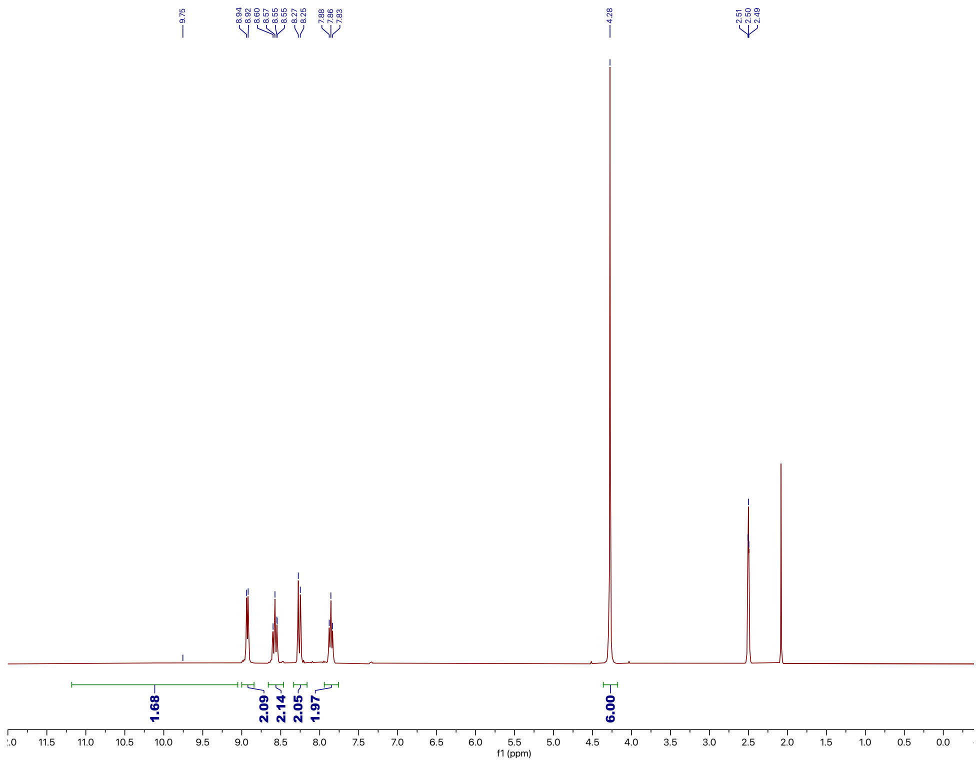

Figure S13: ^{1}H NMR spectrum (DMSO-*d6*, 298 K, 300 MHz) of **2c**

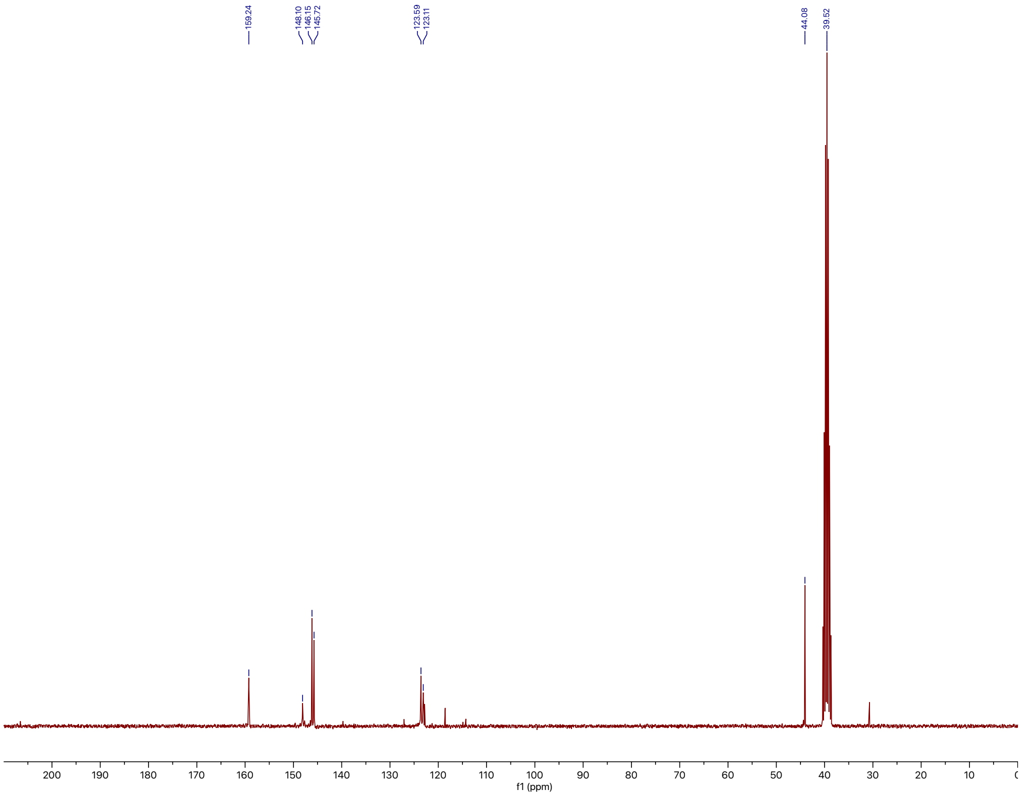

Figure S14: ^{13}C{^{1}H} NMR spectrum (DMSO-*d6*, 298 K, 300 MHz) of **2c**

2c (1 g, 1.78mmol) was suspended under N_2 in dry MeCN (30 ml) then DBU (1.06 mL, 7.11 mmol) was added, and the resulting solution was stirred for 1 h at room temperature. The solvent was then evaporated, and the residue was triturated and filtered in THF (10 mL) to yield **3c** as a pure white crystalline solid (340 mg, 70% yield). Crystals suitable for XRD were grown by slow diffusion of THF in a saturated MeCN solution of **3c**. ^{1}H NMR (300 MHz, CD_3CN, 298K) δ = 8.13 (dd, J_{HH} = 9.0, 1.4 Hz, 2H, δC*H*), 7.77 (dd, J_{HH} = 6.8, 1.8 Hz, 2H, αC*H*), 7.62 (ddd, 2H, J_{HH} = 9.0, 6.8, 1.8 Hz, γC*H*), 6.62 (td, 2H, J_{HH} = 6.8, 1.4 Hz, βC*H*), 3.72 (s, 6H, NC*H*3). ^{13}C{^{1}H} NMR (101 MHz, CD_3CN , 298K) δ = 175.46, (2C, *C*O), 160.44, (2C, C_{PYA}), 141.43 (2C, α*C*H), 140.85 (2C, γ*C*H), 121.02 (2C, δ*C*H), 112.21 (2C, β*C*H), 41.88 (2C, N*C*H3) ppm. HR-ESI-MS (m/z): calculated for $C_{14}H_{15}N_4O_2$ $[M+H]^+$ = 271.1195; found: 271.1191. Elemental analysis calculated for $C_{14}H_{14}N_4O_2$ (%): C 62.21; H 5.22; N 20.73, found: C 61.90; H 5.14; N 20.54.

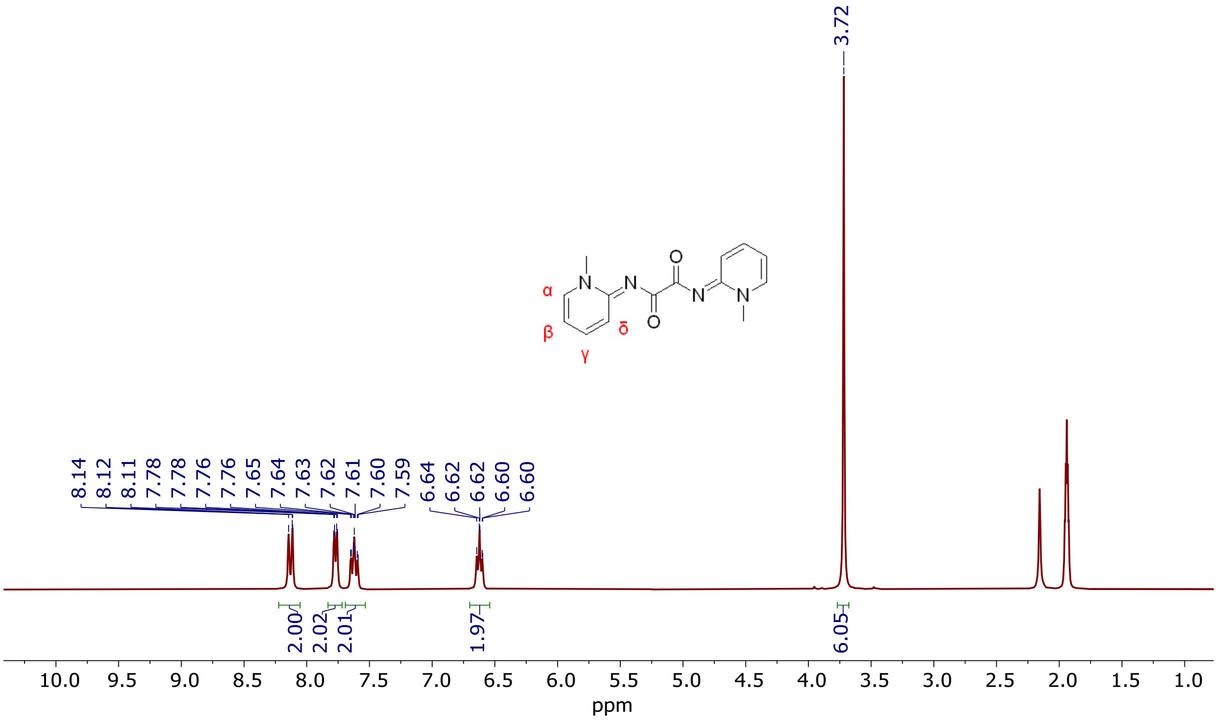

Figure S15: ^{1}H NMR spectrum (CD_3CN, 298 K, 300 MHz) of **3c**

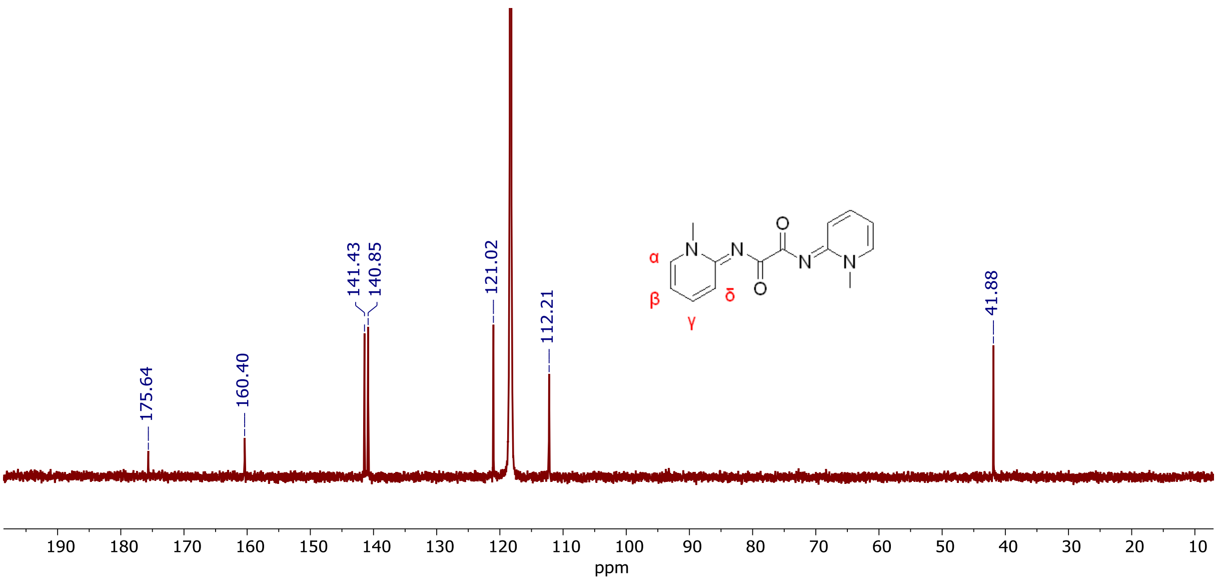

Figure S16: $^{13}C\{^{1}H\}$ NMR spectrum (CD_3CN, 298 K, 101 MHz) of **3c**

S.1.2.4 Reaction of 2c with LiHMDS

2c (275 mg, 0.489 mmol) was suspended under N_2 in dry MeCN (4 mL) then LiHMDS 1M in THF (1.22 mL, 1.22 mmol) was added and the resulting solution was stirred for 1 h at room temperature. The volatiles were evaporated, and the residue was triturated and filtered in THF (2 x 10 mL). The THF-insoluble residue was extracted with MeCN (2 x 10 mL). The combined MeCN fractions were concentrated to dryness to yield a dark yellow solid (115 mg, 54%). Quantitative $^{19}F\{^{1}H\}$ NMR (282 MHz, CD_3CN, 298 K): using 1,2,4,5-tetrafluorobenzene as fluorinated standard revealed a 0.89/1 ratio of PF_6 *vs* ligand unit by comparing ^{1}H NMR and ^{19}F NMR integrations (see Fig S1, S2). ^{1}H NMR (300 MHz, CD_3CN, 298K) δ = 8.01 – 7.93 (m, 4H, ArC*H*), 7.83 (t, J_{HH} = 8.1 Hz, 2H, ArC*H*), 6.92 (t, J_{HH} = 6.8 Hz, 2H, ArC*H*), 3.85 (s, 6H, NCH_3). HR-ESI-MS (m/z): calculated for $C_{14}H_{15}N_4O_2$ $[M+Li]^+$ = 277.1277; found: 277.1269. Elemental analysis calculated for $C_{14}H_{14}N_4O_2$ (%): C 38.20; H 3.66; N 12.73, found: C 38.81; H 3.38; N 13.04.

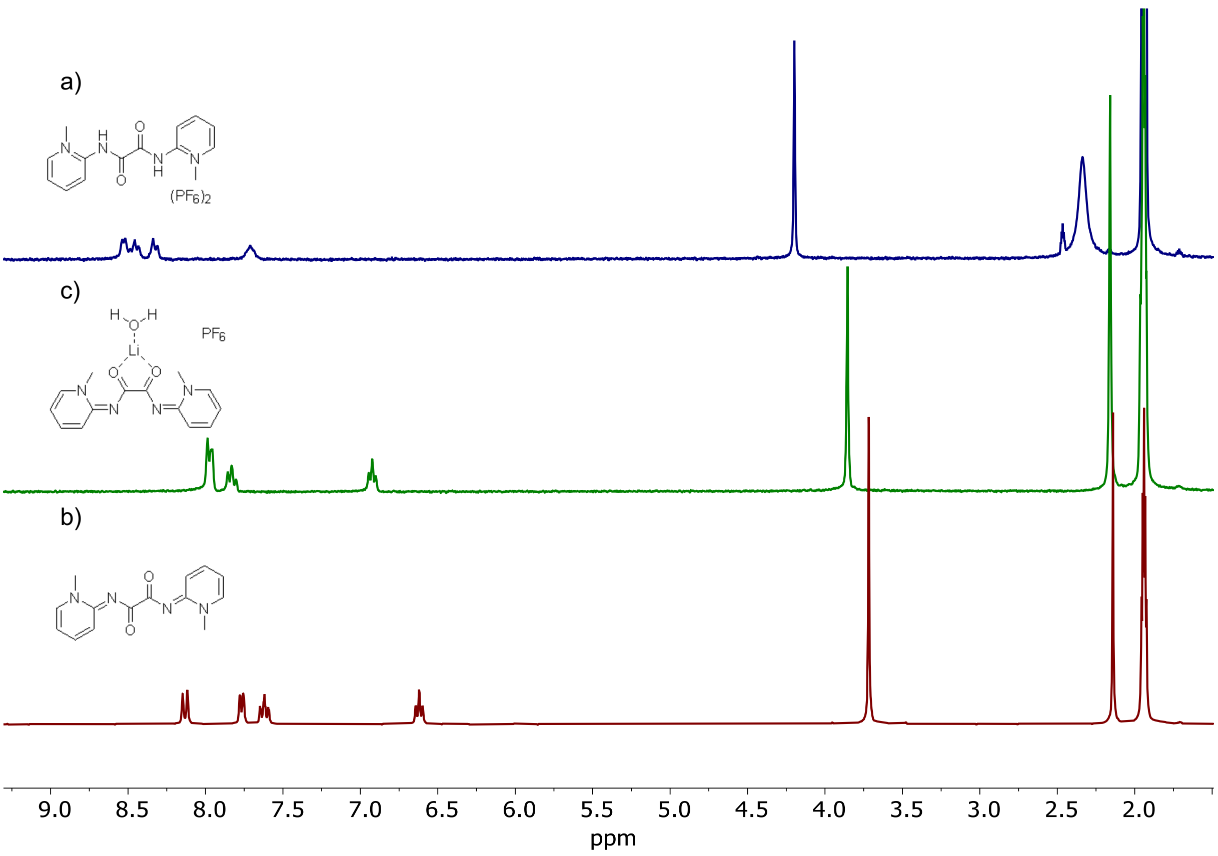

Figure S17: ^{1}H NMR spectrum (CD_3CN, 298 K, 300 MHz) of a) **2c**; b) proposed $LiPF_6$ adduct; c) **3c**.

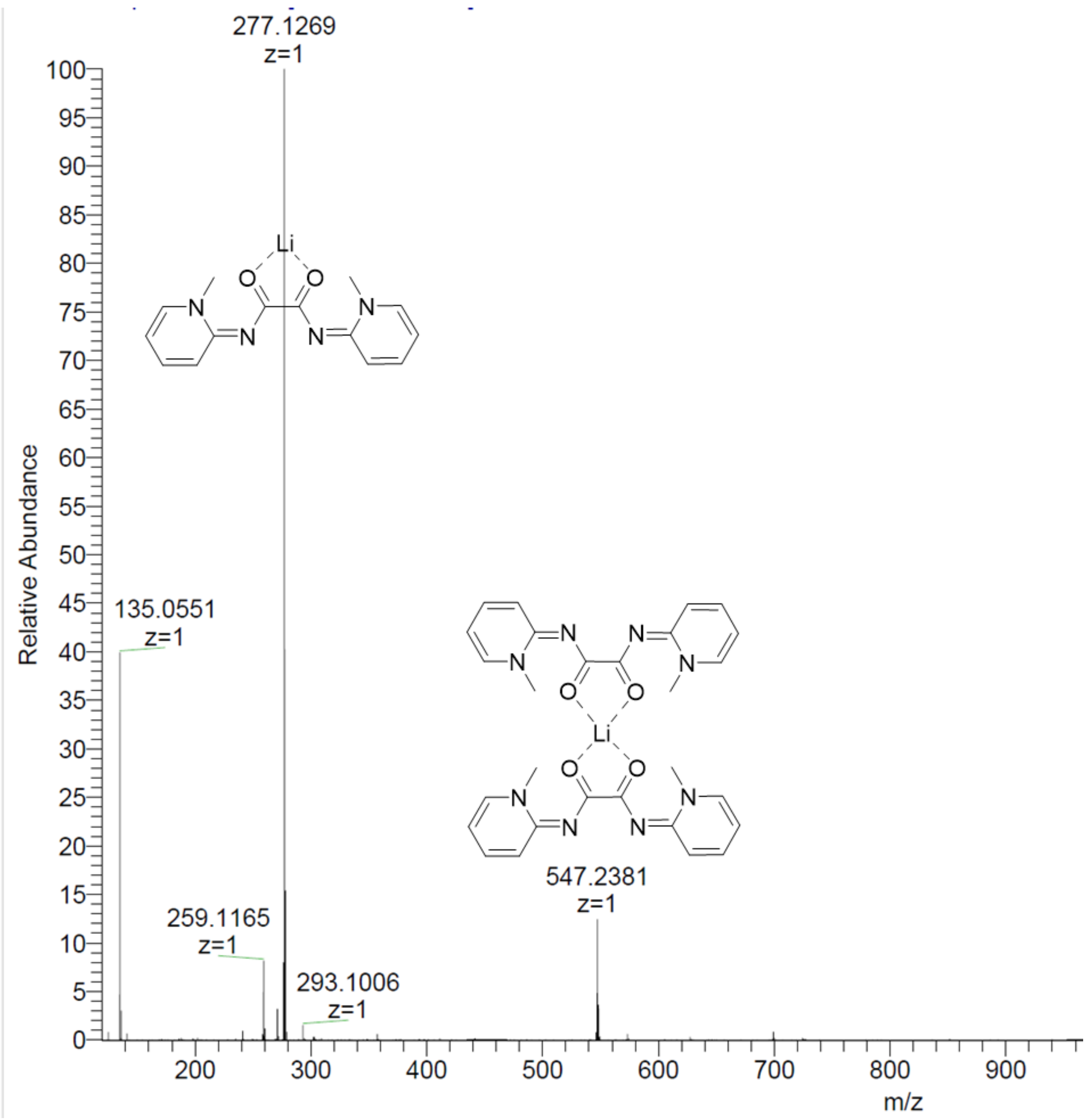

Figure S18: ESI-MS trace of the proposed $LiPF_6$ adduct of **3c** (positive ionization).

S.1.2.5 Synthesis of ligand 2d

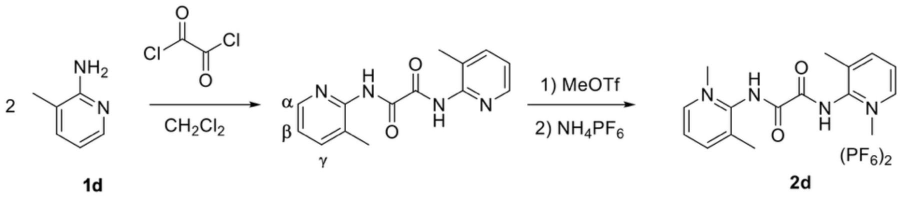

Oxalyl chloride (11.66 mmol, 1.0 mL) was dissolved in dry CH_2Cl_2 (20 mL) under N_2 in a flask equipped with a venting cannula connected to a saturated KOH aqueous solution. To this first solution cooled down to 0 °C, was carefully added dropwise a mixture of 2-amino-3-methylpyridine (26.8 mmol, 2.71 mL) and triethylamine (58.3 mmol, 8.12 mL) under N_2 in dry CH_2Cl_2 (25 mL). The resulting suspension was stirred for 16 h at 23°C then the reaction mixture was filtered, washed with CH_2Cl_2 and dried under high vacuum to afford the bis(pyridine)oxalylamide as a white solid (2.81 g, 80%). ^{1}H NMR (300 MHz, DMSO-d_6, 298K) δ = 10.76 (s, 2H, N*H*) 8.32 (d, $^3J_{HH}$ = 4.8 Hz, 2H, αC*H*), 7.75 (d, $^3J_{HH}$ = 7.5 Hz, 2H, γC*H*), 7.29 (dd, 2H, J_{HH} = 7.5, 4.8Hz, βC*H*), 2.24 (s, 6H, CC*H*$_3$). ^{13}C{^{1}H} NMR (75 MHz, DMSO-d_6, 298K) δ = 159.03 (*C*O), 148.88 (*C*$_{pyr}$), 146.17 (α*C*H), 139.44 (γ*C*H), 129.39 (*C*CH$_3$), 122.70 (β*C*H), 17.23 (C*C*H$_3$) ppm. HR-ESI-MS (m/z): calculated for $C_{14}H_{15}N_4O_2$ $[M+H]^+$ = 271.1195; found: 271.1179.

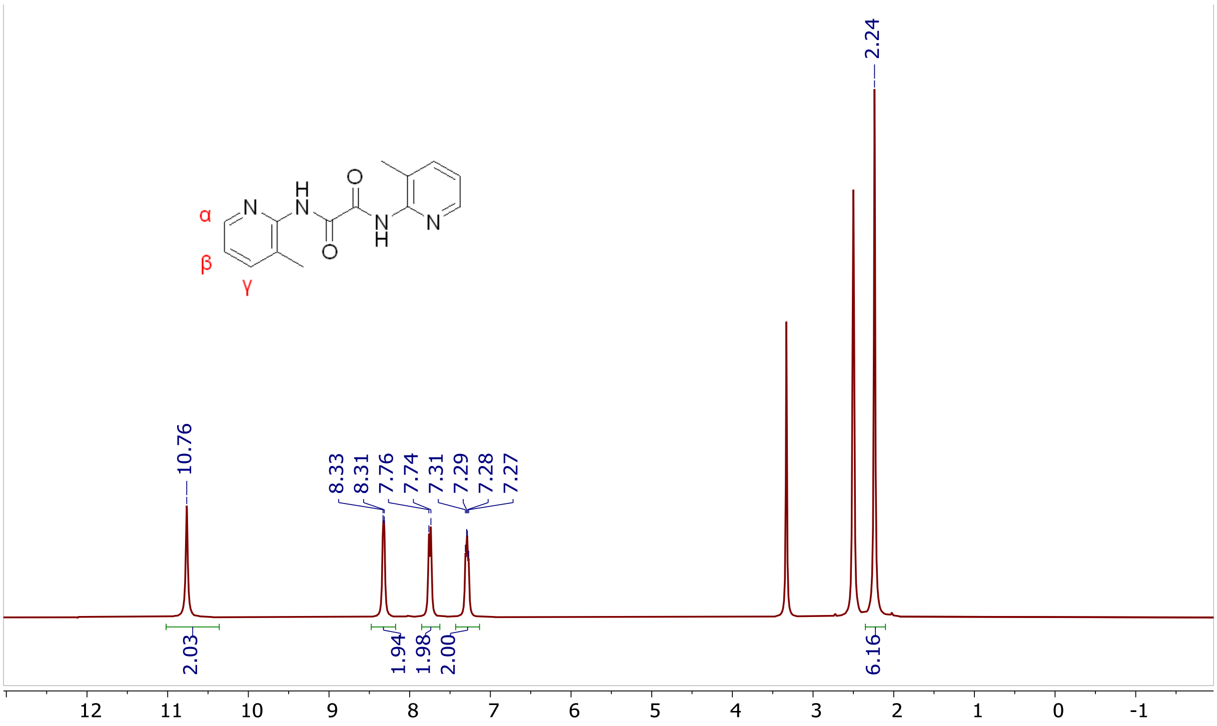

Figure S19: ^{1}H NMR spectrum (DMSO-d_6, 298 K, 300 MHz) of bis(3-methylpyridin-2-yl)oxalylamide.

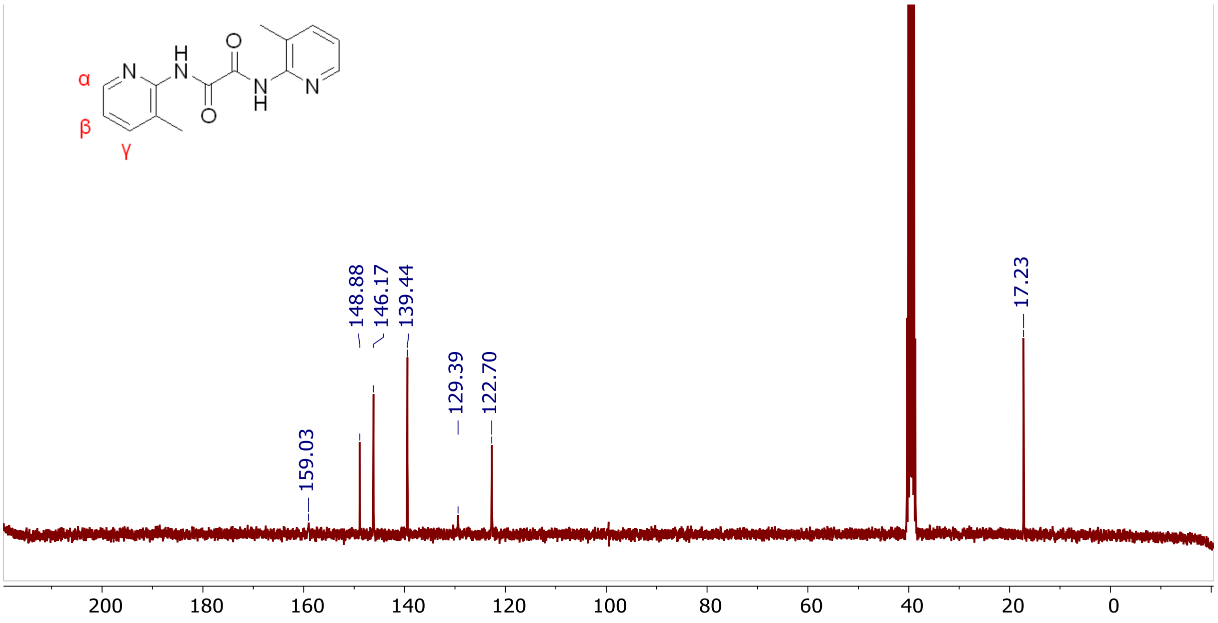

Figure S20: $^{13}C\{^{1}H\}$ NMR spectrum (DMSO-d_6, 298 K, 75 MHz) of of bis(3-methylpyridin-2-yl)oxalylamide.

Bis(3-methylpyridin-2-yl)oxalylamide (790 mg, 2.92 mmol) was suspended in CH_2Cl_2 (25 mL) under N_2, then methyl triflate (1.00 mL, 9.0 mmol) was added and the resulting suspension was stirred for 16 h at 23 °C. The reaction mixture was filtered, the cake was washed with CH_2Cl_2 and dried under vacuo to yield a white solid. The triflate salt was dissolved into as little water as possible and the resulting clear solution was merged with a previously prepared solution of NH_4PF_6 (2.4 g, 14.6 mmol) in as little water as possible. The formed thick slurry was stirred for 20 min, filtered, washed with cold water and dried under vacuo to yield **2d** as a white solid (1.48 g, 86%). Crystals suitable for XRD were grown by slow diffusion of Et_2O in a MeCN **2d** solution. ^{1}H NMR (400 MHz, CD_3CN, 298K) δ = 10.04 (br s, 2H, N*H*) 8.64 (dd, J_{HH} = 6.2, 1.6 Hz, 2H, αC*H*), 8.50 (dd, J_{HH} = 8.1, 1.6 Hz, 2H, γC*H*), 7.94 (dd, $^{3}J_{HH}$ = 8.1, 6.1 Hz, 2H, βC*H*), 4.26 (s, 6H, NCH_3). 2.51 (s, 6H, CCH_3) ppm. $^{13}C\{^{1}H\}$ NMR (101 MHz, CD_3CN, 298K) δ = 157.92 (*C*O), 150.27 (γ*C*H), 146.31 (α*C*H), 144.56 (C_{pyr}), 139.35 (*C*CH_3), 127.58 (β*C*H), 46.67 (N*C*H_3) 18.19 (C*C*H_3) ppm. $^{19}F\{^{1}H\}$ NMR (376 MHz, CD_3CN, 298 K): -72.90 (d, $^{1}J_{FP}$ = 706.7 Hz). $^{31}P\{^{1}H\}$ NMR (162 MHz, DMSO-d_6, 298 K): -144.62 (septet, $^{1}J_{PF}$ = 706.5 Hz). HR-ESI-MS (m/z): calculated for $C_{16}H_{19}N_4O_2$ $[M\text{-}2PF_6\text{-}H]^+$ = 299.1503; found: 299.1501.

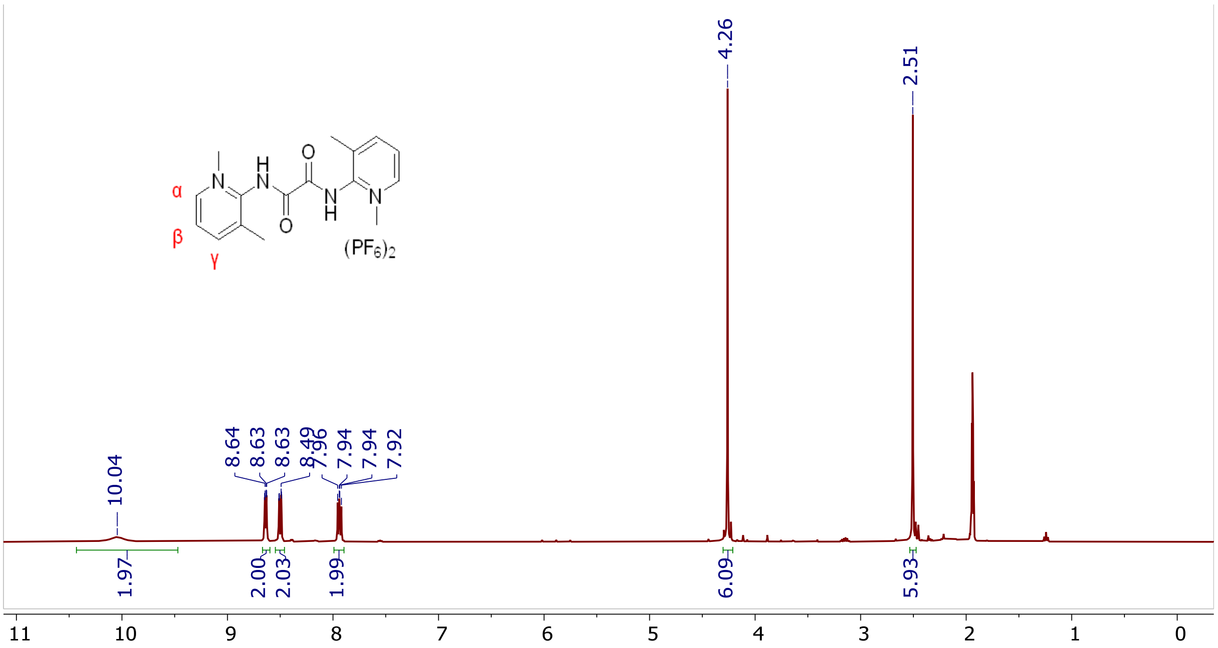

Figure S21: ^{1}H NMR spectrum (CD_3CN, 298 K, 400 MHz) of **2d**

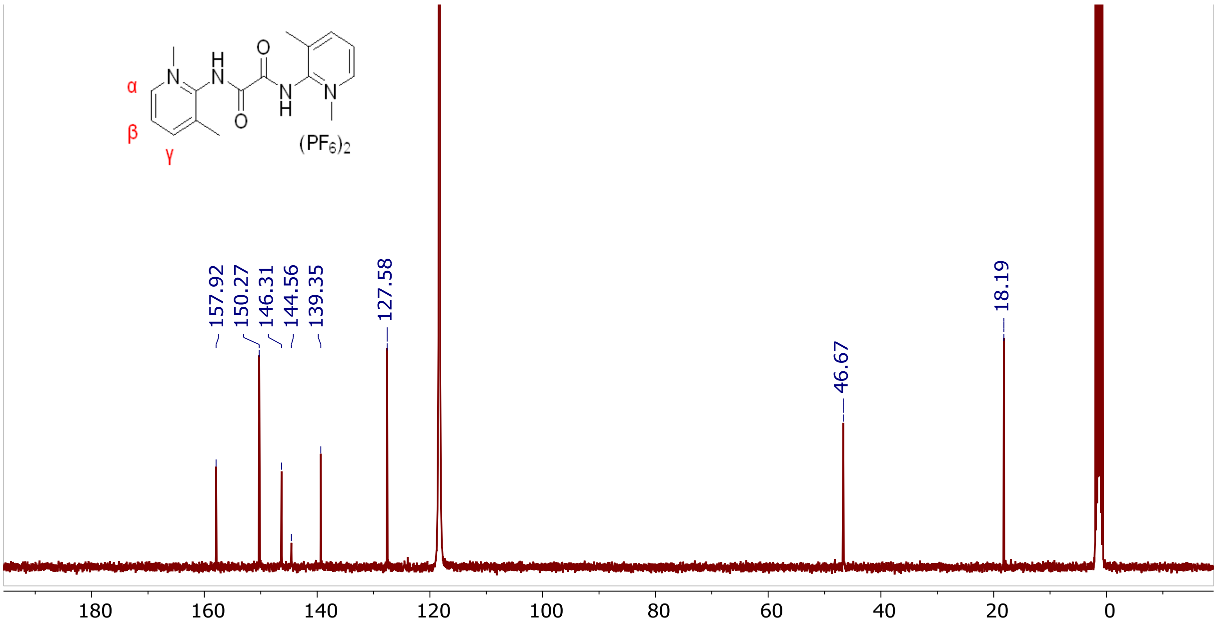

Figure S22: $^{13}C\{^{1}H\}$ NMR spectrum (CD_3CN, 298 K, 101 MHz) of **2d**

S.1.3. Synthesis of neutral complexes 4a–4d

S.1.3.1 Synthesis of complex 4a

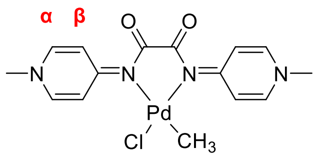

4a

2a (148 mg, 263 μmol), proton sponge (124 mg, 578 μmol) and [PdMeCl(cod)] (77 mg, 290 μmol) were dissolved in dry MeCN (6 mL) under N_2, and the resulting solution was stirred at 23 °C for 2 h. The reaction mixture was filtered, and the cake was washed with dry acetone (2 x 15 mL). The obtained solid was then dried under vacuum to yield **4a** as a yellow solid (90 mg, 80%). Note for the NMR characterization: in DMSO, the complex decays to other species over time, resulting in lower integration the Pd-CH_3 signal and the appearance of sharper signals in the aromatic region. In D_2O only one set of signals is observed excluding the presence of an impurity in the solid material (Figure S25). The rapid reaction of **4a** with the solvent prevented the possibility of characterizing it through ^{13}C NMR spectroscopy in D_2O. ^1H NMR (400 MHz, DMSO-*d*$_6$, 298K) δ = 8.32 (br s, 4H, α-C*H*), 7.99 (br s, 4H, β-C*H*), 4.00 (s, 6H, NCH_3), 0.13 (s, 3H, Pd-CH_3) ppm. $^{13}C\{^1H\}$ NMR (101 MHz, DMSO-*d*$_6$, 298K) δ = 168.14 (*C*O), 161.74 (*C*$_{PYA}$), 142.89 (α-*C*H), 121.51 (β-*C*H), 45.06 N*C*H$_3$), -5.38 (Pd-*C*H$_3$). HR-ESI-MS (m/z): calculated for $C_{17}H_{20}N_5O_2Pd$ [M-Cl]$^+$ = 432.0652; found: 432.0641. Elemental analysis calculated for $C_{15}H_{17}ClN_4O_2Pd$ (%): C 42.17; H 4.01; N 13.12, found: C 42.60; H 4.13; N 13.70. The slightly higher N and C content suggests the presence of about 5% of homoleptic **[Pd(3a)$_2$][Cl]$_2$** arising from the synthesis, that was also detected by ESI-MS. The use of an excess of [PdMeCl(cod)] did not suppress this impurity.

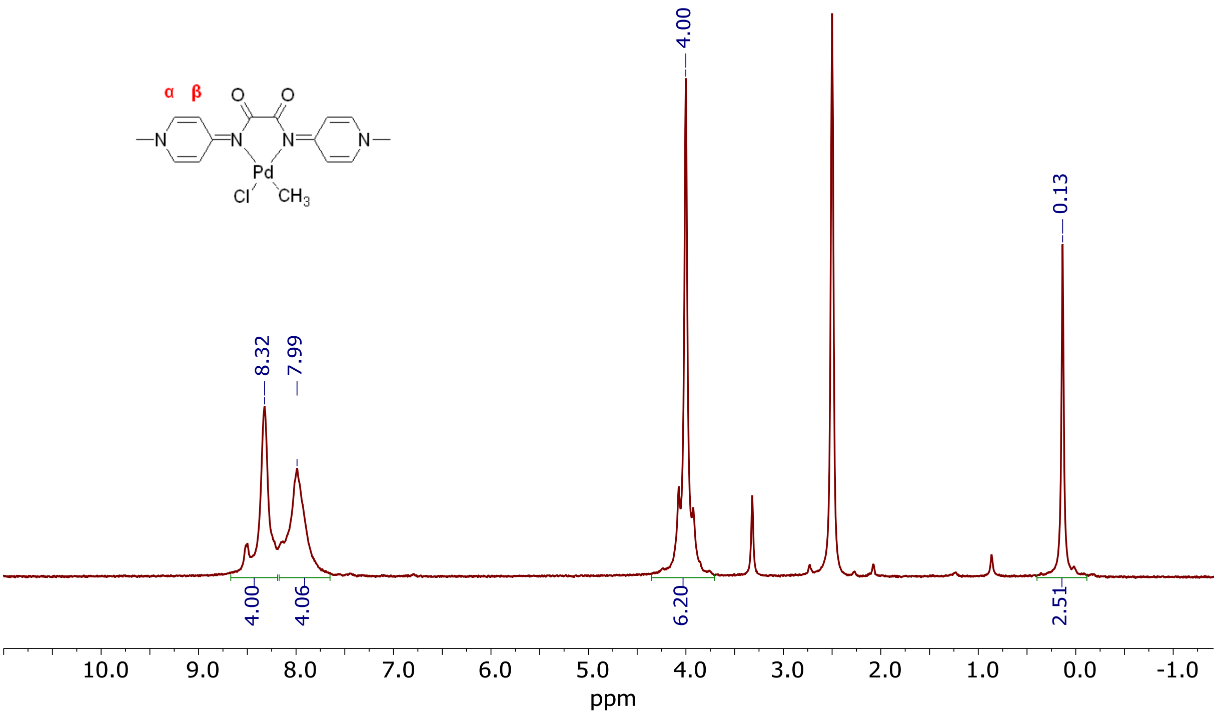

Figure S23: ^1H NMR spectrum (DMSO-*d*$_6$, 298 K, 400 MHz) of **4a**

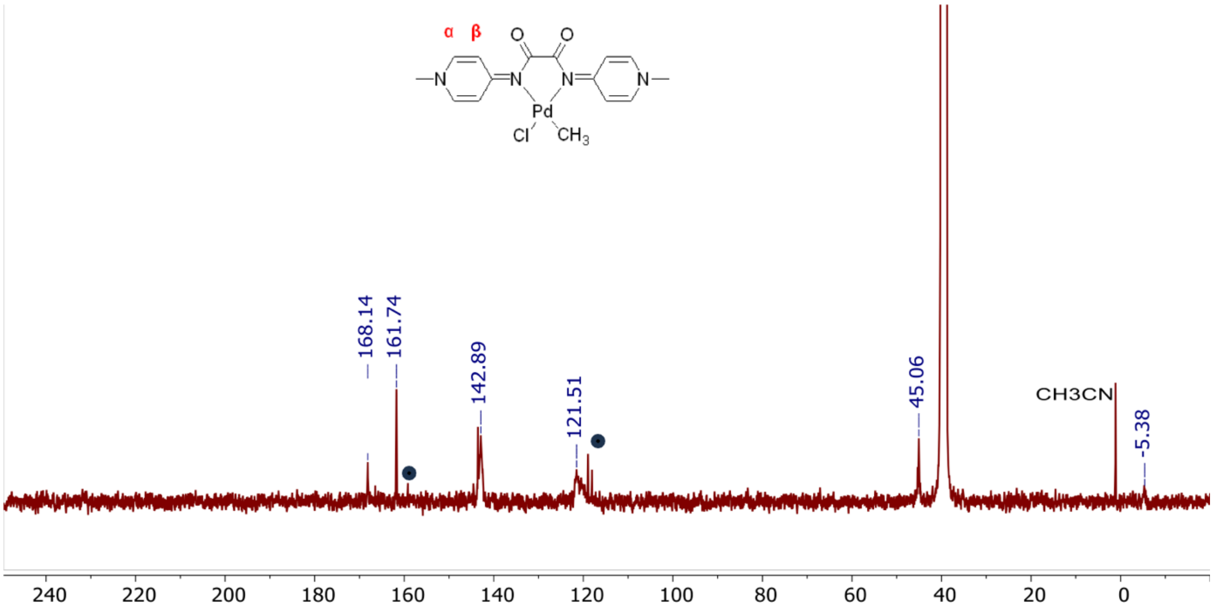

Figure S24: $^{13}C\{^1H\}$ NMR spectrum (DMSO-d_6, 298 K, 75 MHz) of **4a**. Sharp signals attributed to decomposition species [Pd(**3a**)$_2$][Cl]$_2$ are marked with black spheres.

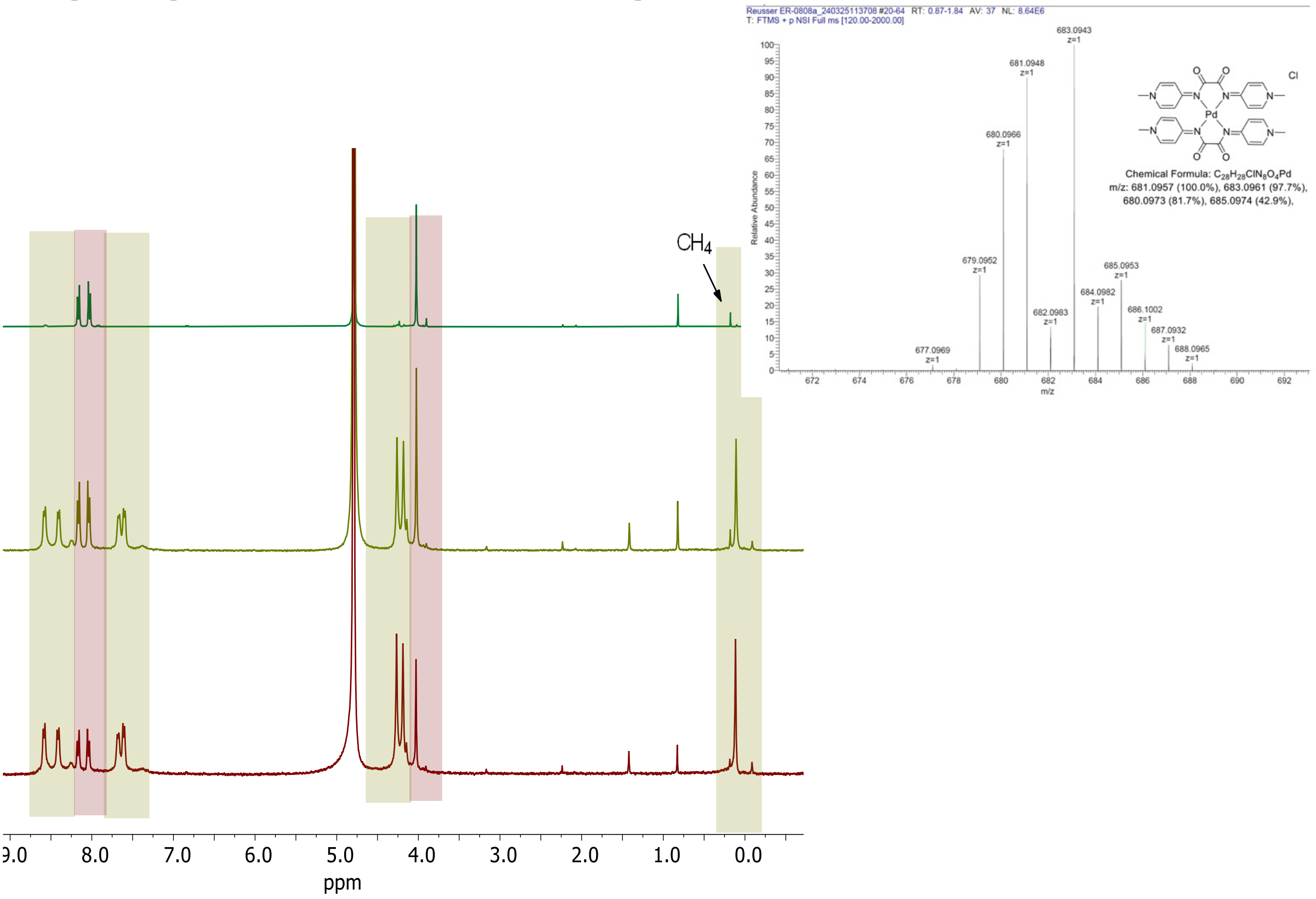

Figure S25: ^1H NMR spectrum (D_2O, 298 K, 300 MHz) of **4a**. Green background marks the signals attributed to **4a**, red background the growing signals attributed to decayed species [Pd(**3a**)$_2$]$^{2+}$. Methane was detected by NMR and upon opening the NMR, pressure was detected likely originating from methane accumulation. ESI-MS analysis of the decayed product indicates the formation of the homoleptic complex [Pd(**3a**)$_2$]Cl_2, found: 681.0948 *m/z*, calculated for $C_{28}H_{28}ClN_8O_4Pd$ [M–Cl]$^+$ = 681.0957.

S.1.3.2 Synthesis of complex 4a'

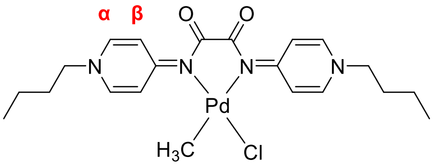

4a'

2a' (113 mg, 175 µmol), proton sponge (83.0 mg, 385 µmol) and [PdMeCl(cod)] (49 mg, 185 µmol) were dissolved in dry MeCN (5 mL) under N_2, and the resulting solution was stirred at 23°C for 2h. The solvent was evaporated, and the residue was suspended in acetone, filtered and washed with the same solvent. The residue was then dried under vacuum to yield **4a'** as a yellow solid (80 mg, 89%). Note for the NMR characterization: in DMSO, the complex gradually decays to another species over time, resulting in a lower integration of the Pd-CH_3 signal and the appearance of sharper signals in the aromatic region. ^{1}H NMR (300 MHz, DMSO-d_6, 298K) δ = 8.42 (br s, 4H, α-C*H*), 8.01 (br s, 4H, β-C*H*), 4.25 (br t, $^3J_{H-H}$ = 7.3 Hz, 4H, NCH_2), 1.79 (pent, $^3J_{H-H}$ = 7.3 Hz, 4H, NCH$_2$CH_2), 1.25 (sext, $^3J_{H-H}$ = 7.3 Hz, 4H, CH_2CH$_3$), 0.90 (t, *J* = 7.3 Hz, 6H, CH$_2$CH_3), 0.15 (s, 3H, Pd-CH_3) ppm. ^{13}C{^{1}H} NMR (101 MHz, DMSO-d_6, 298K) δ = 168.18 (*C*O), 161.88 (C_{PYA}), 141.99 (α-*C*H), 121.32 (β-*C*H), 57.54 N*C*H$_2$), 32.32 (NCH$_2$*C*H$_2$), 18.76 (*C*H$_2$CH$_3$), 13.32 (CH$_2$*C*H$_3$), -5.15 (Pd-*C*H$_3$). HR-ESI-MS (m/z): calculated for $C_{21}H_{29}N_4O_2Pd$ [M-Cl]$^+$ = 473.1325; found: 475.1311. Elemental analysis calculated for $C_{21}H_{29}ClN_4O_2Pd$ (%): C 49.33; H 5.72; N 10.96, found: C 49.25; H 5.72; N 10.96.

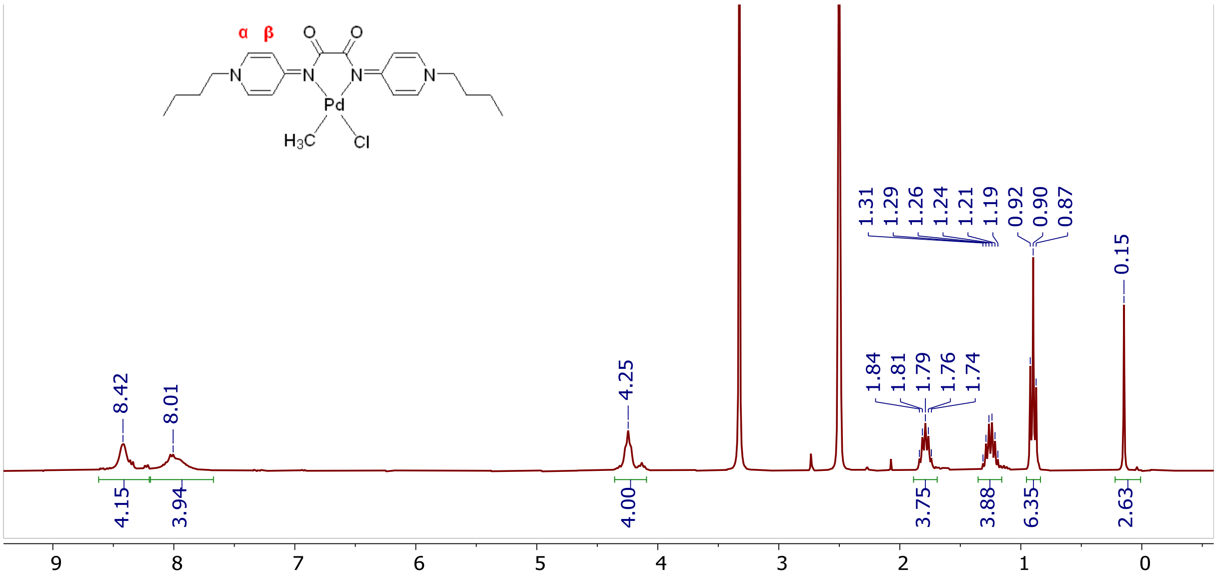

Figure S26: ^{1}H NMR spectrum (DMSO-d_6, 298 K, 300 MHz) of **4a'**

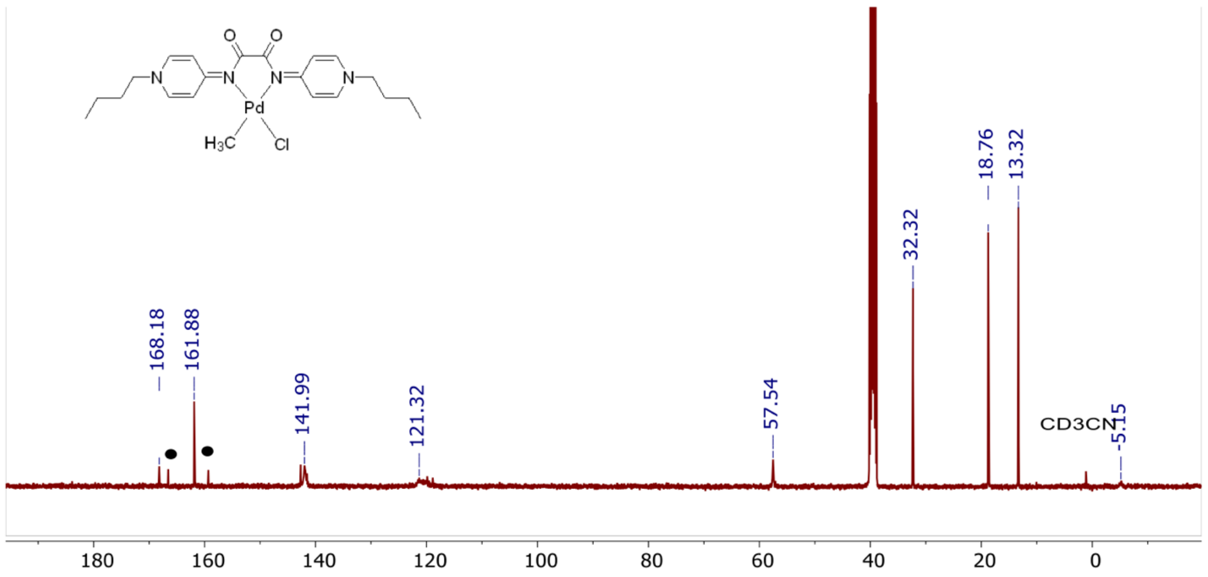

Figure S27: $^{13}C\{^1H\}$ NMR spectrum (DMSO-d_6, 298 K, 101 MHz) of **4a'.** Signals attributed to species resulting from reaction with water **[Pd(3a')$_2$][Cl]$_2$** are marked with black spheres.

S.1.3.3 Synthesis of complex 4b

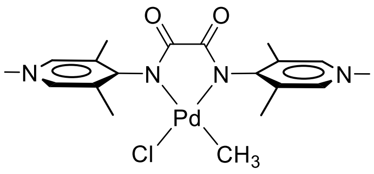

4b

2b (36 mg, 58 μmol), proton sponge (27 mg, 128 μmol) and [PdMeCl(cod)] (16 mg, 61 μmol) were suspended under N_2 in dry MeCN (2 mL) and the resulting mixture was stirred for 2 h at 23 °C. The solvent was then evaporated, and the residue was suspended in acetone (10 mL), filtered, washed with copious amounts of acetone, and dried under vacuum to yield **4b** as a dark yellow solid (24 mg, 86 % yield). NMR analysis revealed a 2:1 ratio of DMSO solvent to chlorido complex **4b** in DMSO. Addition of an excess of NBu_4Cl increased the chloride complex ratio, while addition of $AgPF_6$ totally suppressed the neutral complex signals (Figure S30). Signals attributed to **4b**: ^1H NMR (300 MHz, DMSO-d_6, 298K), δ = 8.56 (s, 2H, C*H*), 8.38 (s, 2H, C*H*), 4.14 (s, 3H, NCH_3), 4.09 (s, 3H, NCH_3), 2.32 (s, 12H, CCH_3), -0.64 (s, 3H, PdCH_3) ppm. $^{13}C\{^1H\}$ NMR (101 MHz, DMSO-d_6, 298K) δ = 166.56 (*C*PYA), 165.15 (*C*PYA), 163.65 (*C*O), 160.96 (*C*O), 142.00 (*C*H), 140.63 (*C*H), 133.59 (*C*CH_3), 132.60 (*C*CH_3), 46.07 (NCH_3), 45.47 (NCH_3), 15.73 (CCH_3), 14.97 (CCH_3), -7.99 (Pd-CH_3) ppm. Signals attributed to the DMSO solvento complex: ^1H NMR (300 MHz, DMSO-d_6, 298K), δ = 8.69 (s, 2H, C*H*), 8.50 (s, 2H, C*H*), 4.19 (s, 3H, NCH_3), 4.14 (s, 3H, NCH_3), 2.26 (s, 12H, CCH_3), -0.47 (s, 3H, PdCH_3) ppm. $^{13}C\{^1H\}$ NMR (101 MHz, DMSO-d_6, 298K) δ = 167.45 (*C*PYA), 164.25 (*C*PYA), 163.53 (*C*O), 161.18 (*C*O), 142.73 (*C*H), 141.50 (*C*H), 134.28 (*C*CH_3), 133.94 (*C*CH_3), 46.49 (NCH_3), 46.03 (NCH_3), 15.26 (CCH_3), 15.11 (CCH_3), 0.30 (Pd-CH_3) ppm. HR-ESI-MS (m/z): calculated for $C_{18}H_{21}N_4O_2Pd$ [M-Cl-CH_4]$^+$ = 431.0699; found: 431.0702.

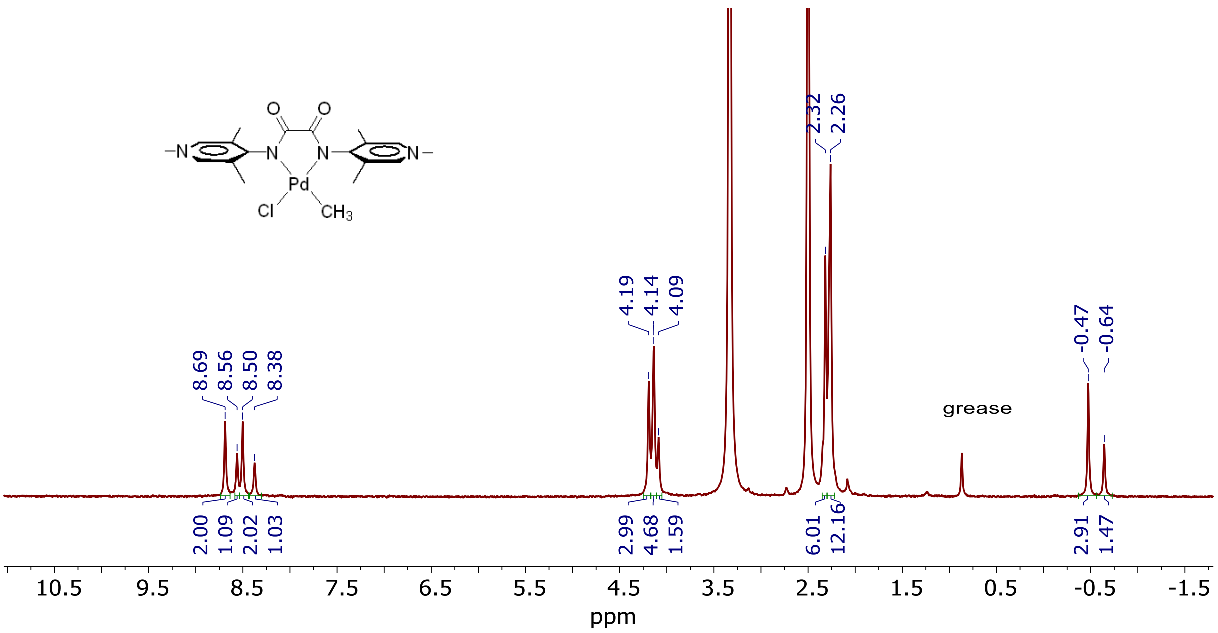

Figure S28: ^{1}H NMR spectrum (DMSO-*d*$_6$, 298 K, 300 MHz) of **4b**

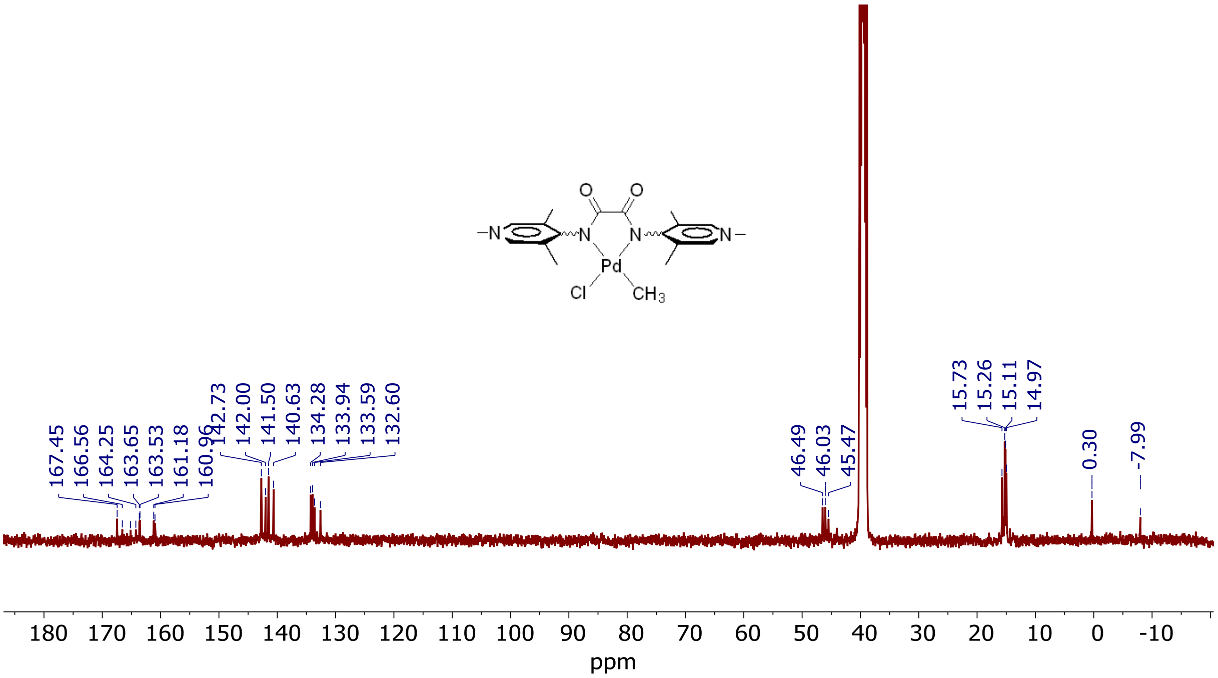

Figure S29: ^{13}C{^{1}H} NMR spectrum (DMSO-*d*$_6$, 298 K, 101 MHz) of **4b**

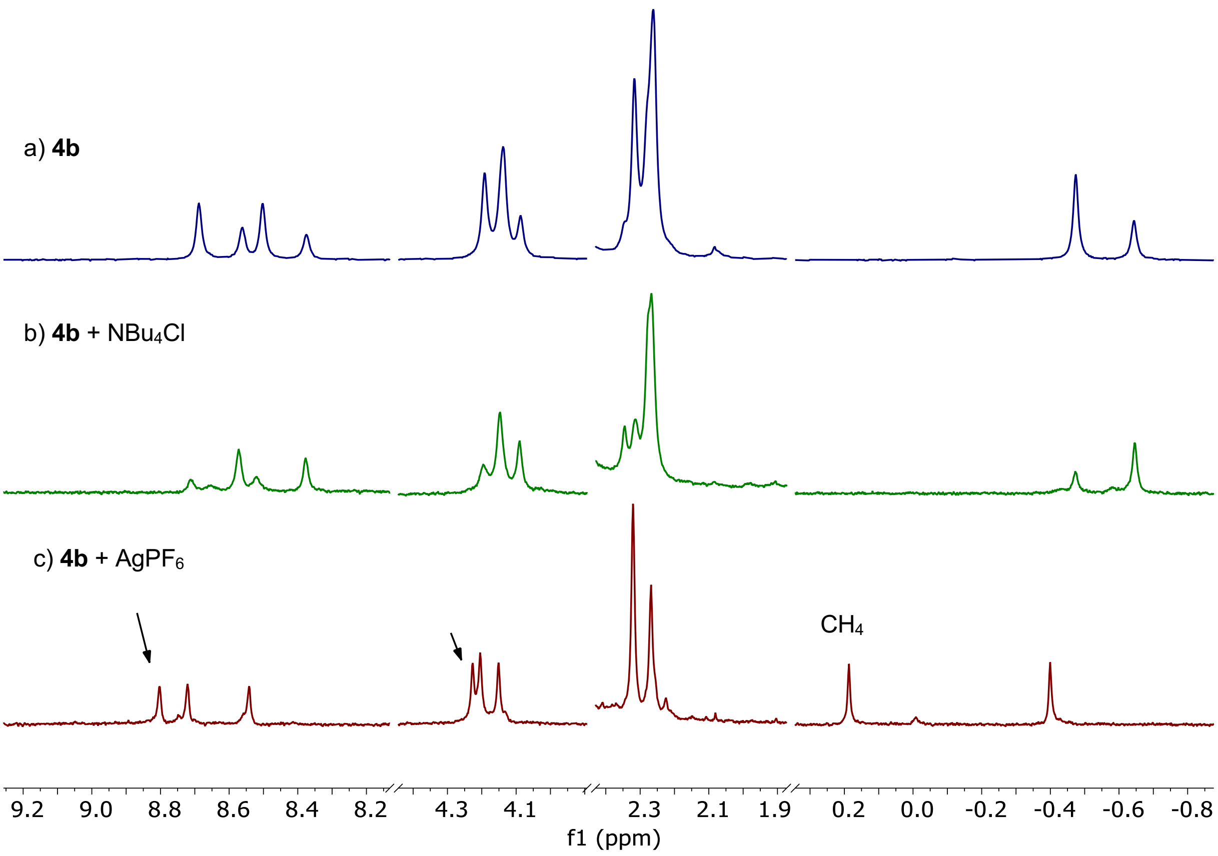

Figure S30: Selected ppm range of ^{1}H NMR spectra (DMSO-d_6, 298 K, 300 MHz) of: a) complex **4b**; b) **4b** in the presence of an excess of NBu_4Cl after 30 min at 23 °C; c) **4b** in the presence of an excess $AgPF_6$ after 30 min at 23 °C, arrows point to the signals of a newly formed species. Together with the detection of CH_4 this indicates that the solvento complex partially decomposes in the presence of $AgPF_6$.

S.1.3.4 Synthesis of complex 4c

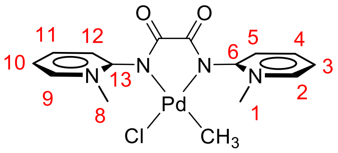

4c

3c (60 mg, 0.22 mmol) and [PdMeCl(cod)] were dissolved in MeCN (4 mL) and the quickly formed suspension was stirred at 23 °C for 2 h. The solvent was then evaporated, and the resulting solid was triturated in pentane (3 x 5 mL) and dried under vacuum to yield **4c** as a pale yellow solid (76 mg, 80% yield). Absolute conformation at the Pd center was demonstrated by 1D NOE correlation between the Pd-methyl and proton H^5. ^{1}H NMR (300 MHz, D_2O, 298K), δ = 8.69 (d, $^3J_{HH}$ = 6.2 Hz, 1H, H^2), 8.55 (d, $^3J_{HH}$ = 6.2 Hz, 1H, H^9), 8.48 (t, $^3J_{HH}$ = 7.9 Hz, 1H, H^4), 8.33 (t, $^3J_{HH}$ = 9.4 Hz, 1H, H^{11}), 7.72 (m, 2H, H^3&H^5), 7.66 (br s, 1H, H^{12}), 7.56 (t, $^3J_{HH}$ = 7.2 Hz, 1H, H^{10}), 4.28 (s, 6H, NCH_3), –0.10 (s, 3H, Pd–CH_3). $^{13}C\{^1H\}$ NMR (101 MHz, D_2O, 298K) δ = 167.72 (CO), 164.24 (CO), 158.20 (C^{13}), 157.22 (C^6), 146.29 (C^4H), 145.05 ($C^{11}H$), 144.50 (C^2H), 143.49 (C^9H), 126.79 (C^5H), 125.20 ($C^{12}H$), 123.26 (C^3H), 121.58 ($C^{10}H$), 43.32 & 43.24 (2 x NCH_3), –7.67 (Pd–CH_3) ppm. Chemical shifts were referenced with respect to CH_2Cl_2 (53.87 ppm) as an internal standard. HR-ESI-MS (m/z): calculated for $C_{17}H_{20}N_5O_2Pd$ $[M–Cl]^+$ = 432.0652; found: 432.0674. Elemental analysis calculated for $C_{15}H_{17}ClN_4O_2Pd \cdot 0.2\ H_2O$ (%): C 41.82; H 4.07; N 13.01, found: C 41.44; H 3.94; N 12.78.

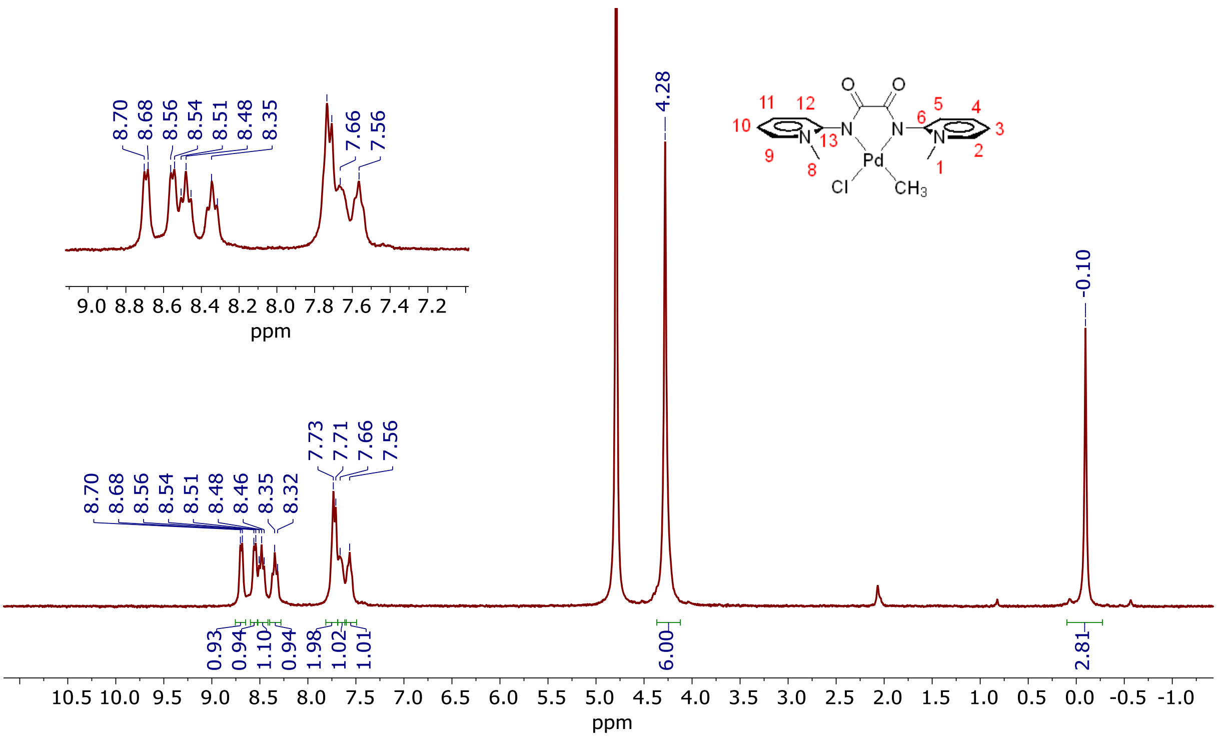

Figure S31: ^{1}H NMR spectrum (D_2O, 298 K, 300 MHz) of **4c**. Inset: zoomed view on the aromatic region.

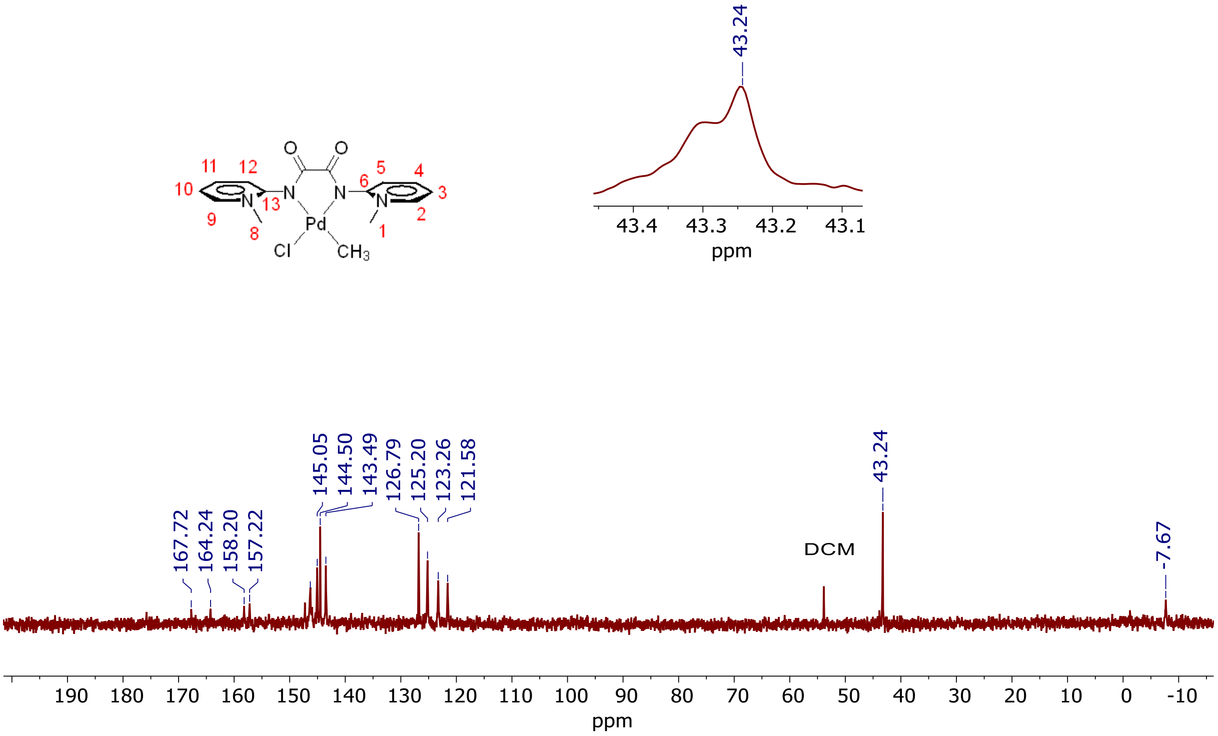

Figure S32: $^{13}C\{^{1}H\}$ NMR spectrum (D_2O, 298 K, 101 MHz) of **4c**. Inset: Zoomed view on the signals of 1&8-carbons.

S.1.3.5 Synthesis of complex 4d

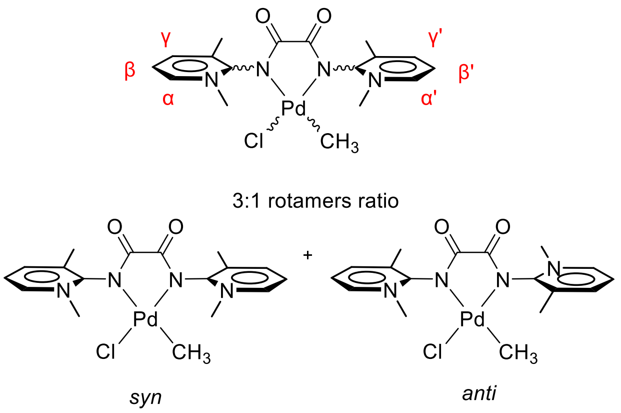

2d (200 mg, 0.34 mmol), proton sponge (158 mg, 0.74 mmol) and [PdMeCl(cod)] (93 mg, 0.35 mmol) were suspended under N_2 in dry MeCN (10 mL) and the resulting mixture was stirred for 2 h at 23°C. The solvent was evaporated, and the residue was suspended in acetone and filtered (10 mL). The residue was washed with copious amounts of acetone and dried under vacuum to yield **4d** as a yellow solid (100 mg, 70% yield). NMR analysis revealed the presence of 4 sets of signals. Upon exposure to DMSO, the chloride anion is exchange yielding the solvent complex. This species accounts for 20 % of the whole population and was fully suppressed upon addition of an excess of NBu_4Cl (Figure S36). Furthermore, the chloride complex **4d** exists as a mixture of rotamers in a ~3:1 ratio, presumably the *syn* and *anti*-configuration. The existance of rotamers at room temperature was demonstrated by variable temperature ^{1}H NMR, i.e the two sets of signals coalesced at elevated temperature (~ 348 K, Figure S35). The similarity of the two sets of signals prevented definitive assignment of the pair of rotamers. Similarly, absolute configuration determination at the Pd centre was precluded due to the similarity of the two PYA ring chemical shifts and the atoms belonging to the more upfield shifted ring were named α' - δ' Signals attributed to **4d**: ^{1}H NMR (400 MHz, DMSO-d_6, 298K), δ = 8.73 (d, $^3J_{HH}$ = 6.4 Hz, 1H, α'-C*H*), 8.57 (d, $^3J_{HH}$ = 7.4 Hz, 1H, α-C*H*), 8.36 (d, $^3J_{HH}$ = 7.7 Hz, 1H, γ'-C*H*), 8.16 (d, $^3J_{HH}$ = 7.4 Hz, 1H, γ-C*H*), 7.63 (dd, $^3J_{HH}$ = 7.7, 6.4 Hz, 1H, β'-C*H*), 7.42 (t, $^3J_{HH}$ = 7.4 Hz, 1H, β-C*H*), 4.22 – 4.13 (m, 6H, NCH_3), 2.45 – 2.35 (m, 6H, CCH_3), -0.58 (apparent doublet, 3H, $PdCH_3$). ^{13}C{^{1}H} NMR (101 MHz, CD_3CN, 298K) δ = 164.42 (*C*O), 160.94 (*C*O), 160.18 (C_{PYA}), 158.77 (C_{PYA}), 145.27 (γ'-*C*H), 143.57 (γ-*C*H), 141.69 (α'-*C*H), 140.54 (α-*C*H), 135.08 (CCH_3), 134.09 (CCH_3), 121.27 (β'-*C*H), 119.39 (β-*C*H), 43.27 – 42.76 (2C, NCH_3), 17.68 – 17.02 (2C, CCH_3) -10.63 ($PdCH_3$) ppm. Signals attributed to the DMSO solvent complex: ^{1}H NMR (400 MHz, DMSO-d_6, 298K), δ = 8.84 (br s, 1H, α'-C*H*), 8.62 (br s,1H, α-C*H*), 8.46 (br s, 1H, γ'-C*H*), 8.24 (br s, 1H, γ-C*H*), 7.77 (br s, $^3J_{HH}$ = 7.7, 6.4 Hz, 1H, β'-C*H*), 7.57 (t, $^3J_{HH}$ = 7.4 Hz, 1H, β-C*H*), 4.22 – 4.13 (m, 6H, NCH_3), 2.45 – 2.35 (m, 6H, CCH_3), -0.47 (s, 3H, $PdCH_3$). ^{13}C{^{1}H} NMR (101 MHz, CD_3CN, 298K) δ = 43.27 – 42.76 (2C, NCH_3), 17.68 – 17.02 (2C, CCH_3) -2.02 ($PdCH_3$) ppm. Aromatic carbons assignment precluded due to low concentration. ^{1}H NMR (300 MHz, D_2O, 298K), δ = 8.57 (d, $^3J_{HH}$ = 6.2 Hz, 1H, α'-C*H*), 8.45 (d, $^3J_{HH}$ = 6.3 Hz, 1H, α-C*H*), 8.39 (d, $^3J_{HH}$ = 7.8 Hz, 1H, γ'-C*H*), 8.27 (d, $^3J_{HH}$ = 7.7 Hz, 1H, γ-C*H*), 7.66 (dd, $^3J_{HH}$ = 7.8, 6.2 Hz, 1H, β'-C*H*), 7.53 (dd, $^3J_{HH}$ = 7.7, 6.3 Hz, 1H, β-C*H*), 4.30 – 4.25 (m, 6H, NCH_3), 2.55 – 2.48 (m, 6H, CCH_3), -0.24 (s, 3H, $PdCH_3$). HR-ESI-MS (m/z): calculated for $C_{19}H_{24}N_5O_2Pd$ $[M-Cl+MeCN]^+$ = 460.0965; found: 460.0953. Elemental analysis calculated for $C_{17}H_{21}ClN_4N_4O_2Pd \cdot 0.5\ H_2O$ (%): C 43.98; H 4.78; N 12.07, found: C 44.03; H 4.56; N 11.74.

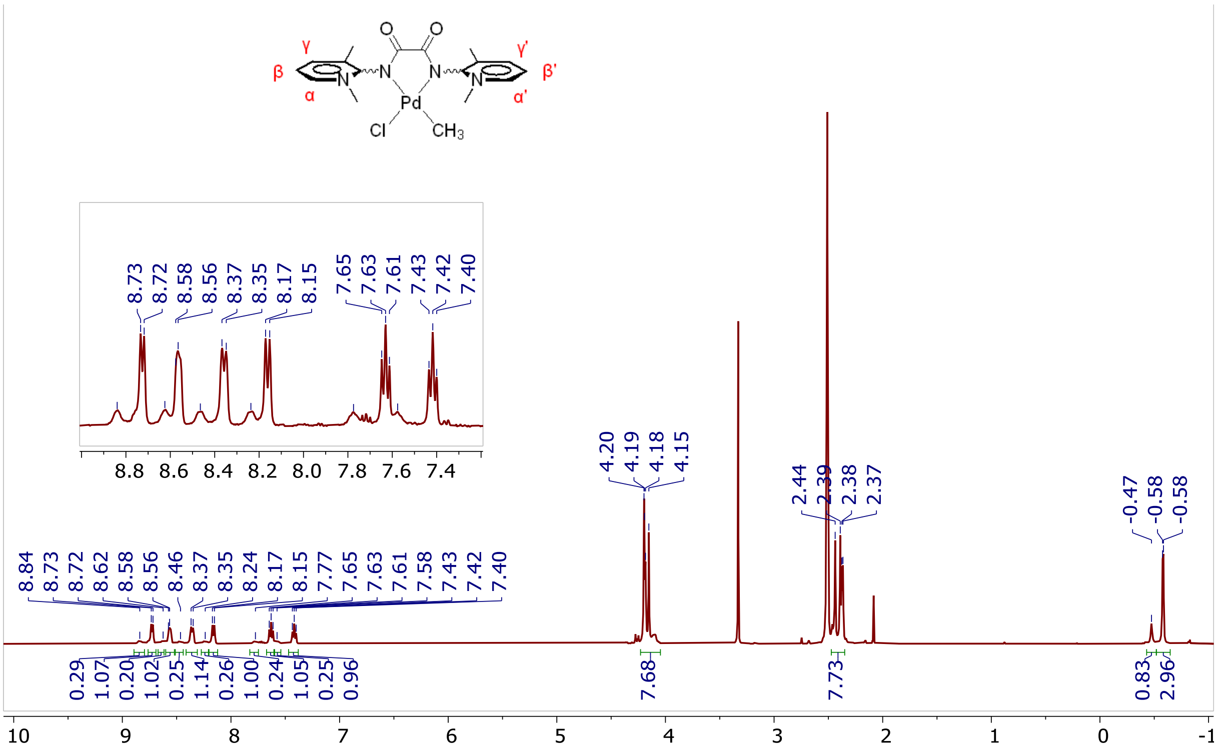

Figure S33. ^{1}H NMR spectrum (DMSO-d_6, 298 K, 400 MHz) of **4d**

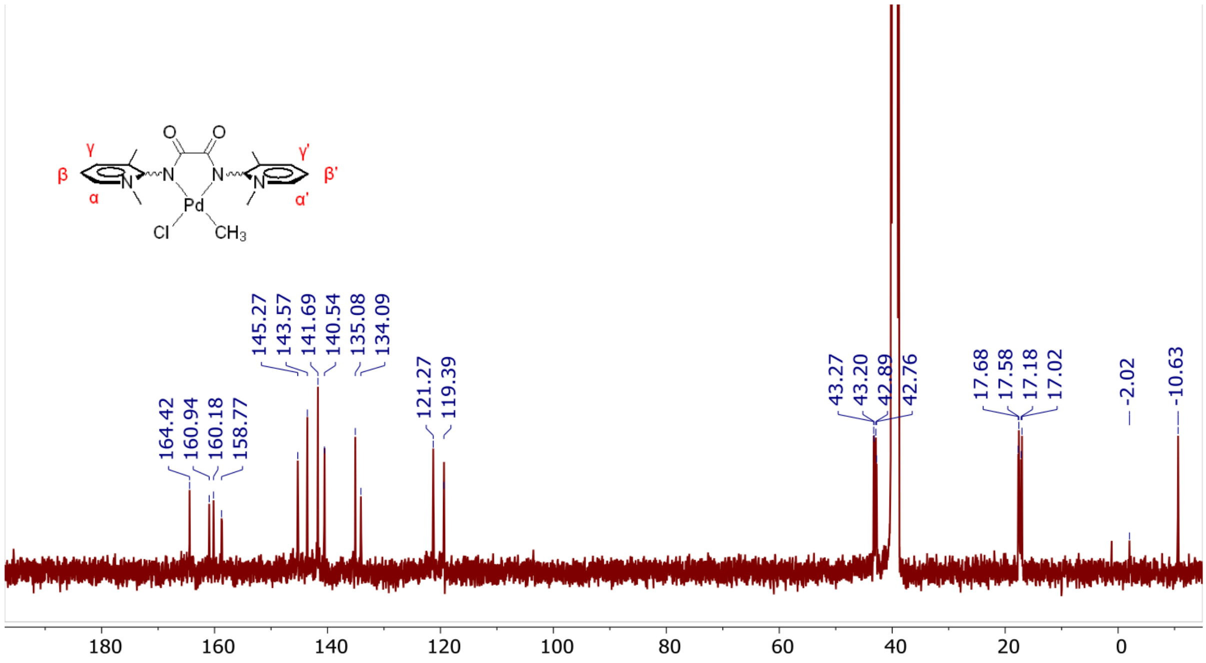

Figure S34. $^{13}C\{^{1}H\}$ NMR spectrum (DMSO-d_6, 298 K, 101 MHz) of **4d**

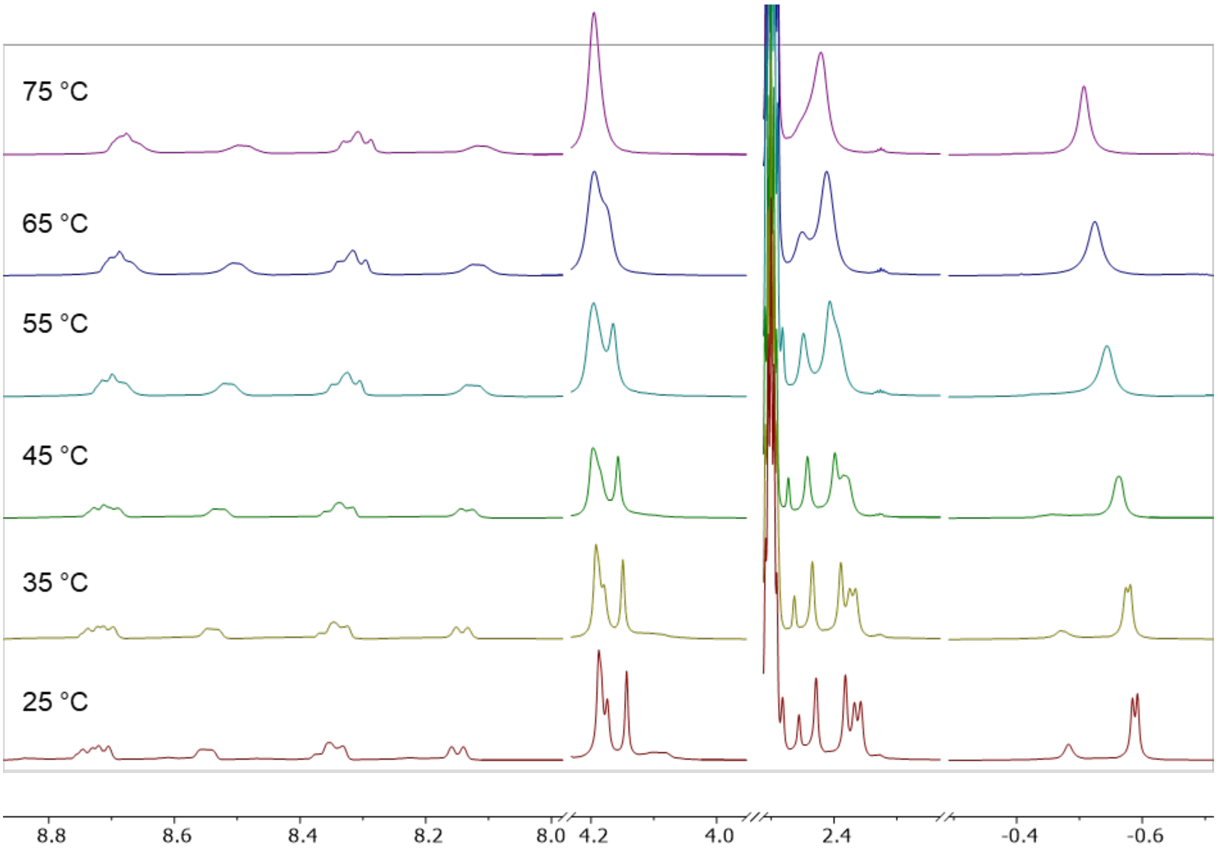

Figure S35. Selected ppm range for the variable temperature ^{1}H NMR spectrum (DMSO-d_6, 298–348 K, 400 MHz) of **4d** showing the coalescence of both the solvento and chlorido signals (Figure S36) at ca. 50 °C, and of the characteristic rotamers signals at 75 °C Free ligand signals are observed due to lack of stability over time in DMSO-d_6 (reaction with H_2O) the intensity of that set of signals did not increase during the VT measurement indicating that no thermal degradation was observed. Also, the resolution of that set of signal did not decrease at elevated temperature, indicating that the broadening of the signals belonging to **4d** is due to exchange dynamics and not shimming or natural broadening effects. The difference in chemical shifts between the N-CH_3 resonances ($\Delta\nu$) in Hz and the estimated coalescence temperature (T_c) were used to estimate the *syn/anti* rotation energy barrier ($\Delta G^‡$) according to the derived Eyring equation.[S6] It is generally assumed that the Eyring equation (1) provides a reasonable estimate of $\Delta G^‡$ when the exchange temperature between two equally populated sites has been reached.[S6] This condition is not reached here since there is clearly the presence of a major and a minor set of isomers in an approximate 3:1 ratio. For this reason, the calculated $\Delta G^‡$ = 17.9 kcal/mol (eq. 2) should be considered as an approximation only.

$$\Delta \boldsymbol{G}^{‡} = \boldsymbol{RT_c}\left[\mathbf{22.96} + \boldsymbol{ln}\left(\frac{T_c}{\Delta\nu}\right)\right] \quad (1)$$

$$\Delta \boldsymbol{G}^{‡} = 8.31 * 348.15\left[\mathbf{22.96} + \boldsymbol{ln}\left(\frac{348.15}{18}\right)\right] = 75.00\,\frac{kJ}{mol} \cong 17.9\,\frac{kcal}{mol} \quad (2)$$

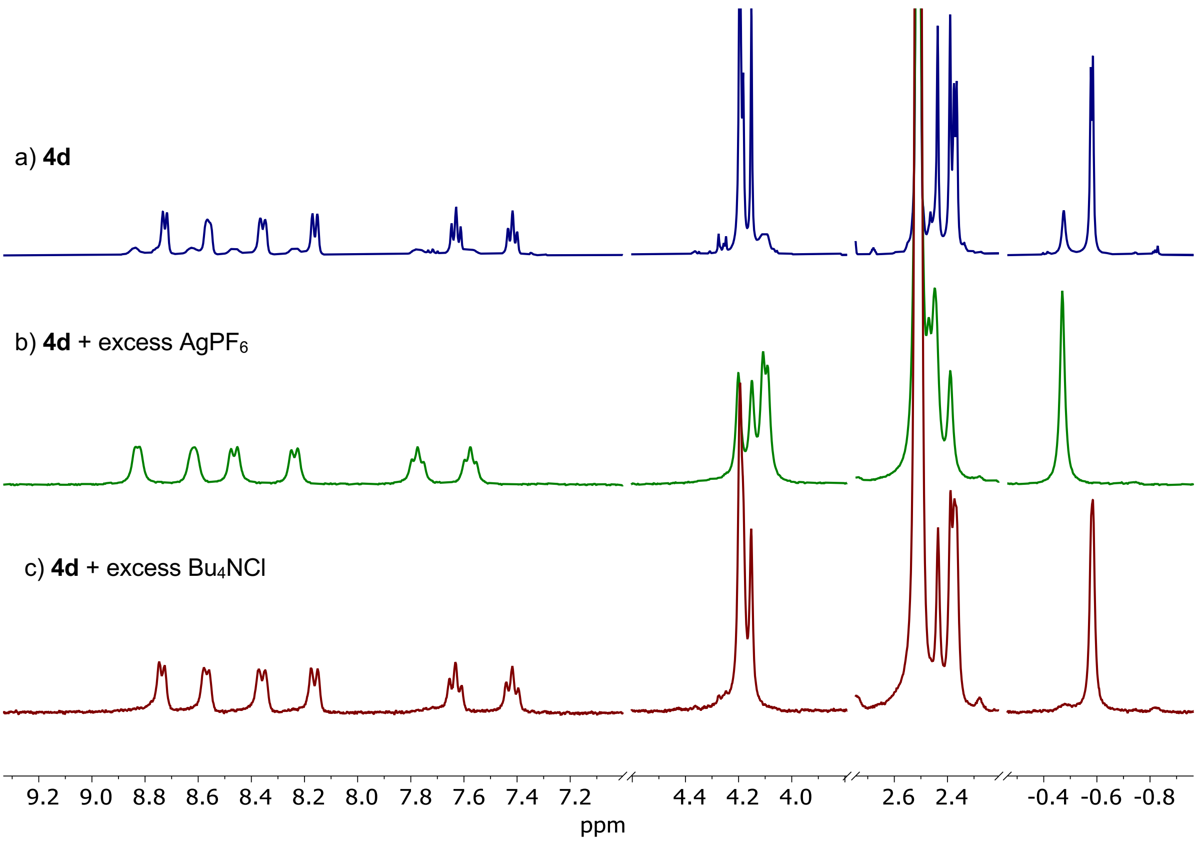

Figure S36: Selected ppm range of ^{1}H NMR spectrum (DMSO-*d*$_6$, 298 K, **3**00 MHz) of: a) complex **4d**; b) **4d** in the presence of an excess of $AgPF_6$ after 30 min at 23 °C; c) **4d** in the presence of Bu4NCl after 30 min at 23 °C.

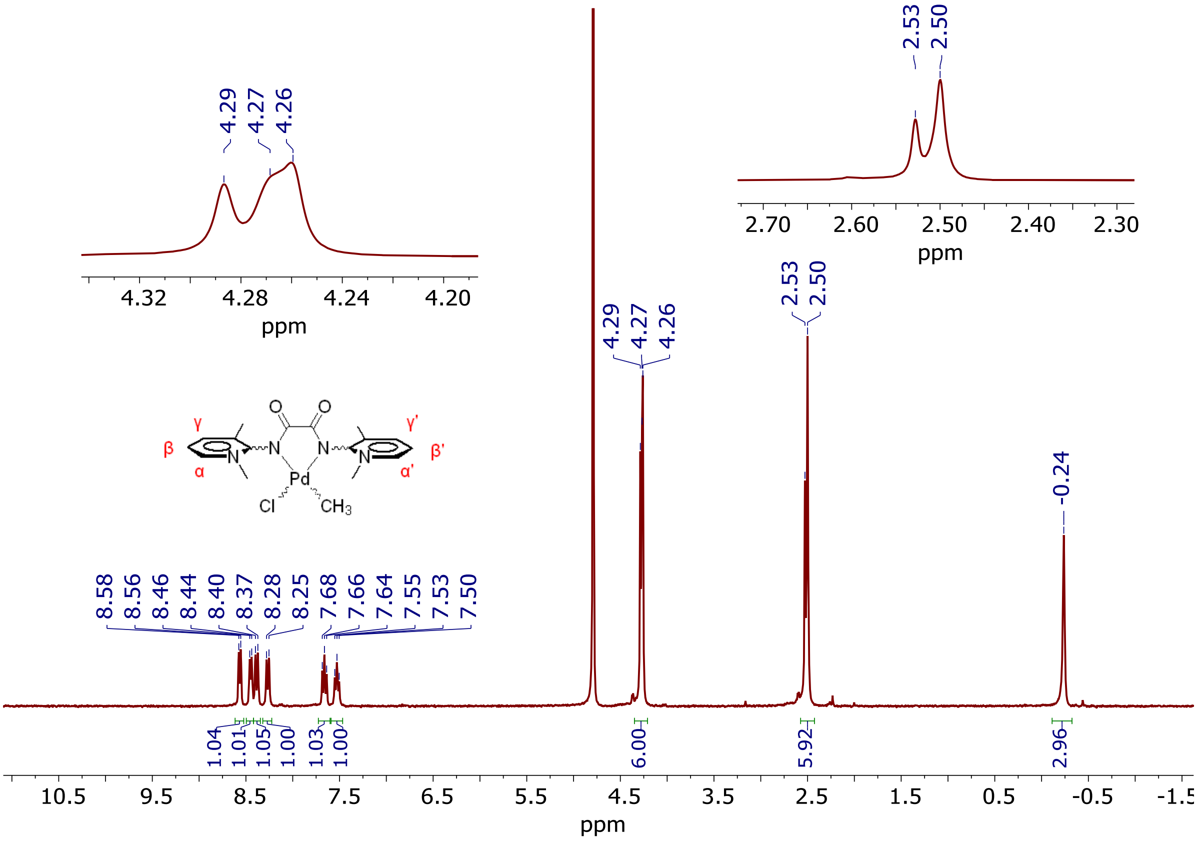

Figure S37: ^{1}H NMR spectrum (D_2O, 298 K, 300 MHz) of **4d.**

Table S1. Pd–CH_3 chemical shifts of complexes **4a–d** and typical [Pd(CH_3)Cl (N,N)] olefin polymerization precatalyst.

Pre-catalyst	δ_H(Pd-CH_3)	Solvent
**4a**	0.13	DMSO-d_6
**4a**	0.11	D_2O
**4a'**	0.15	DMSO-d_6
**4b**	-0.47[a]; -0.64	DMSO-d_6
**4c**	-0.39	DMSO-d_6
**4c**	-0.10	D_2O
**4d**	-0.47[a]; -0.58	DMSO-d_6
**4d**	-0.24	D_2O
α-diimine S7	0.51	CD_2Cl_2
py-NHC S8[b]	0.01	DMSO-d_6
py-PYA S9	-0.24	CD_2Cl_2
PhO⁻NHC S10[c]	-0.81	CD_3Cl

[a] chloride substituted by DMSO; [b] chloride ligand substituted by bromide; [c] chloride substituted by 2,6-lutidine.

S.1.4. Synthesis of cationic complexes

S.1.4.1 Synthesis of complex 5a

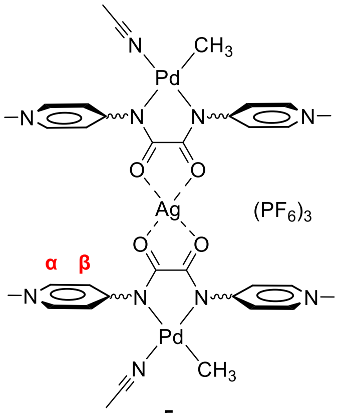

5a

Compound **2a** (200 mg, 356 µmol), Ag_2CO_3 (103 mg, 373 µmol) and [PdMeCl(cod)] (94 mg, 0.36 mmol) were charged in a 20 mL vial and the atmosphere was changed to N_2. Dry MeCN (8 mL) was added, and the resulting suspension was stirred at 23 °C for 90 min. The reaction mixture was filtered through a plug of celite. and the Celite washed with MeCN (5 mL). The combined filtrates were concentrated to 1 mL and triturated into pentane. The solid was collected and recrystallized from MeCN/Et_2O and dried under vacuum to yield **5a** as a yellow solid (169 mg, 67%). ^{1}H NMR (300 MHz, DMSO–d_6, 298K) δ = 8.15 (br, 4H, α-C*H*), 8.08 (br, 4H, α-C*H*), 7.93 (br, 4H, β-C*H*), 7.83 (br, 4H, β-C*H*), 4.02 (s, 6H, NCH_3), 3.99 (s, 6H, NCH_3), 1.96 (s, 6H, CH_3CN), 0.29 (s, 6H, Pd-CH_3) ppm. ^{13}C{^{1}H} NMR (101 MHz, DMSO-d_6, 298K) δ = 164.05 (C_{PYA}), 144.34 (α-*C*H), 124.72 (β-*C*H), 121.46 (β-*C*H), 46.71 N*C*H_3), 46.52 N*C*H_3), –2.49 (Pd-*C*H_3), carbonyl carbon assignment precluded due to poor solubility. ^{19}F{^{1}H} NMR (376 MHz, CD_3CN, 298 K): –72.92 (d, $^1J_{FP}$ = 706.4 Hz) ppm. ^{31}P{^{1}H} NMR (162 MHz, CD_3CN, 298 K): –144.62 (septet, $^1J_{PF}$ = 706.4) ppm. Quantitative ^{19}F{^{1}H} NMR (282 MHz, CD_3CN, 298 K): using 1,2,4,5-tetrafluorobenzene as fluorinated standard (*cf* Figures S1, S2) revealed a 1.45/1 ratio of PF_6 *vs* ligand unit by comparing ^{1}H NMR and ^{19}F NMR integrations. HR-ESI-MS (m/z): calculated for $C_{17}H_{20}AgF_6N_5O_2PPd$ [M-PF_6]$^+$ = 683.9345; found: 683.9339. Elemental analysis calculated for $C_{34}H_{40}A_gF_{18}N_{10}O_4P_3Pd_2$ (%): C 29.00; H 2.86; N 9.95, found: C 28.79; H 2.88; N 9.58.

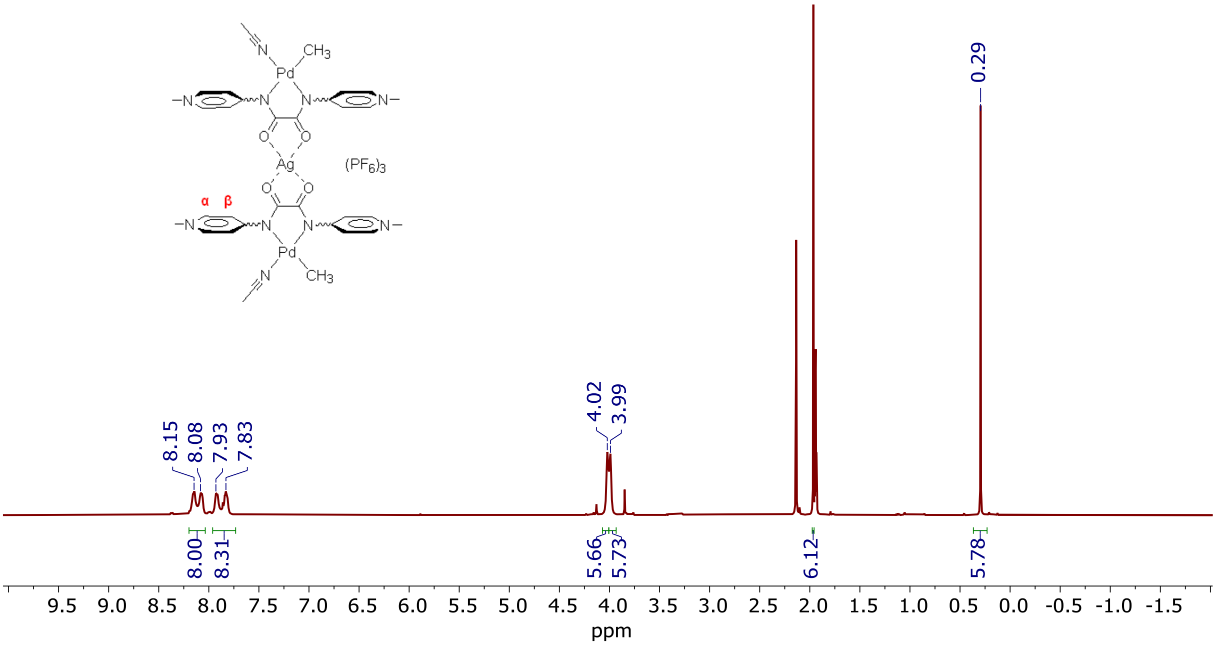

Figure S38: ^{1}H NMR spectrum (CD_3CN, 298 K, 400 MHz) of **5a**.

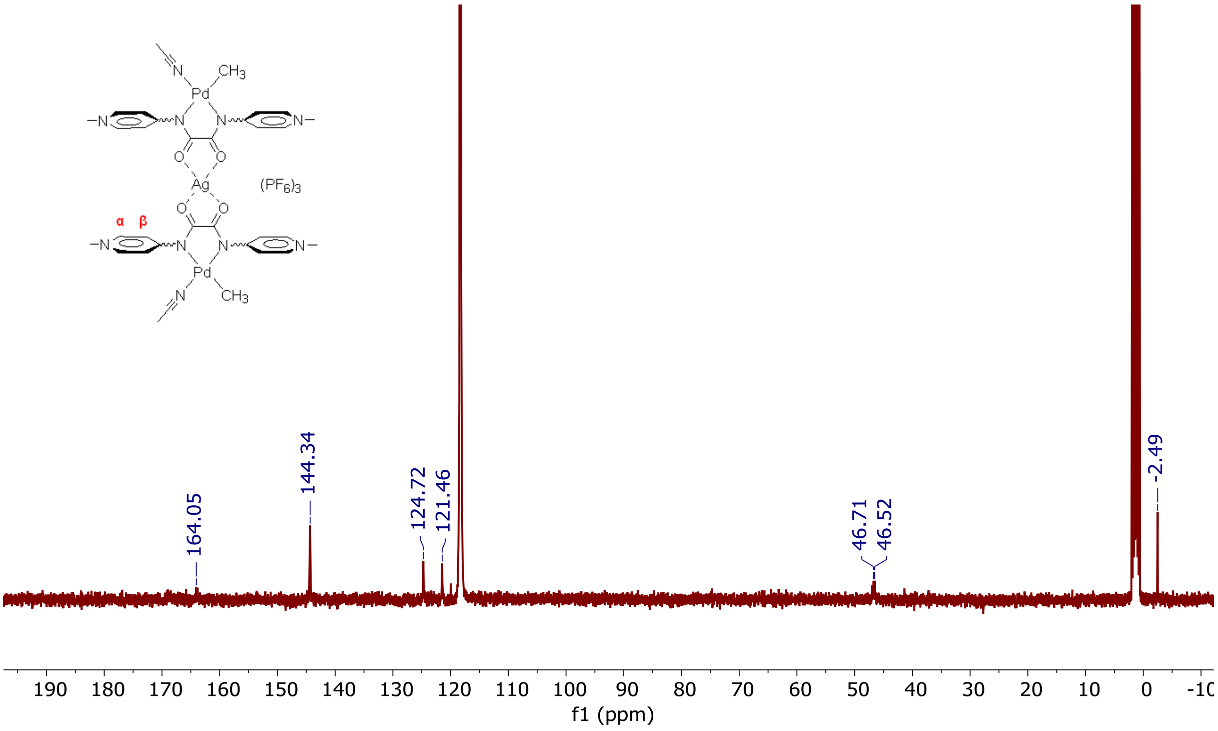

Figure S39: $^{13}C\{^{1}H\}$ NMR spectrum (CD_3CN 298 K, 101 MHz) of **5a**.

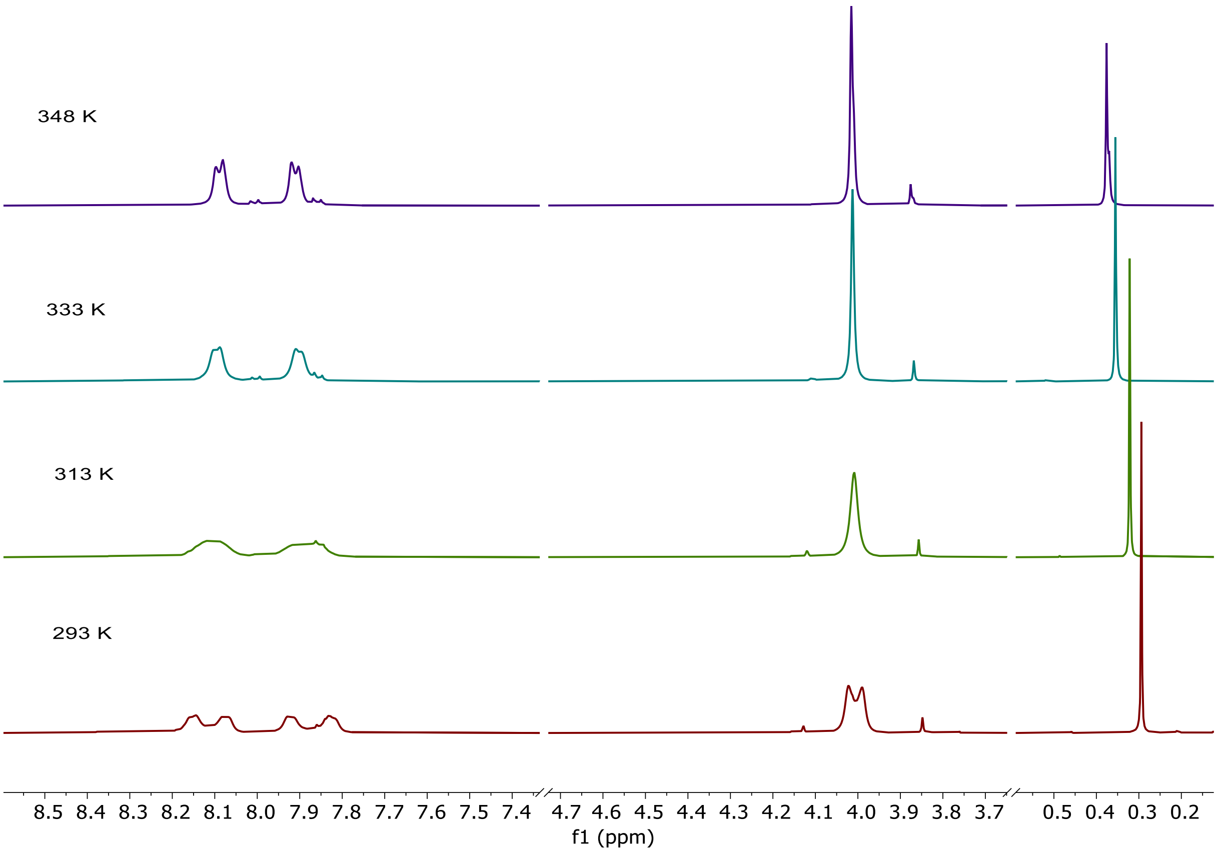

Figure S40: Selected ppm range of the variable temperature ^{1}H NMR spectrum (CD_3CN, 293–348 K, 400 MHz) of **5a**.

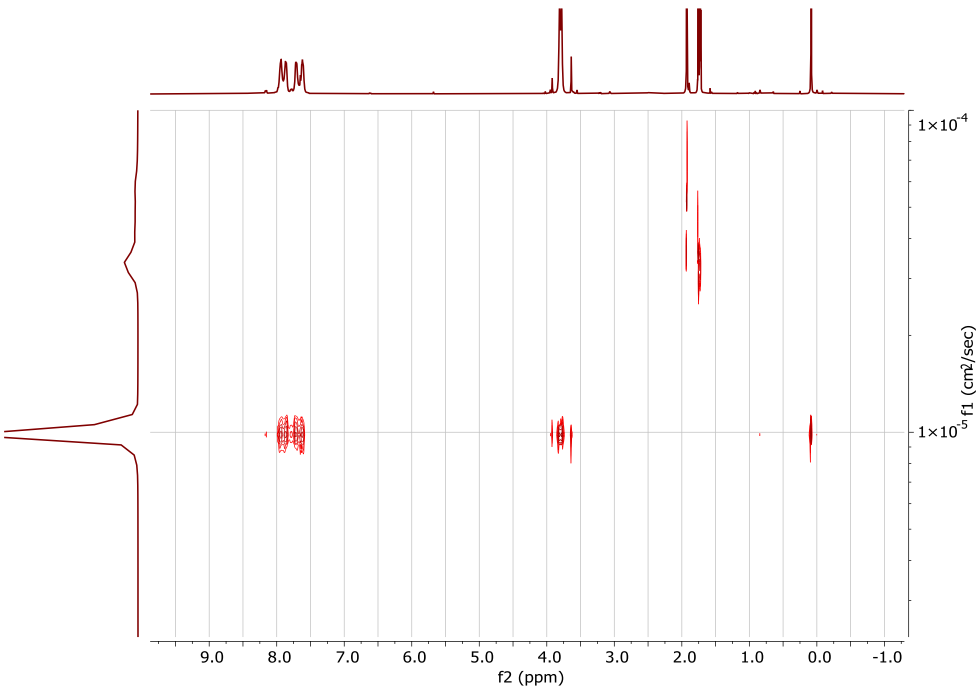

Figure S41: ^{1}H DOSY NMR spectrum (CD_3CN, 298 K, 400 MHz) of **5a**. All signals corresponding to NC*H*3 have similar diffusion coefficient ~ (9.3 x 10^{-6} cm^2/s) giving an approximate molecular weight of 930 g/mol which corresponds to the molecular weight of the Ag-bridged dimer in solution (973 g/mol) considering a loose ion pairing as expected in a polar solvent like MeCN.[S11–S13]

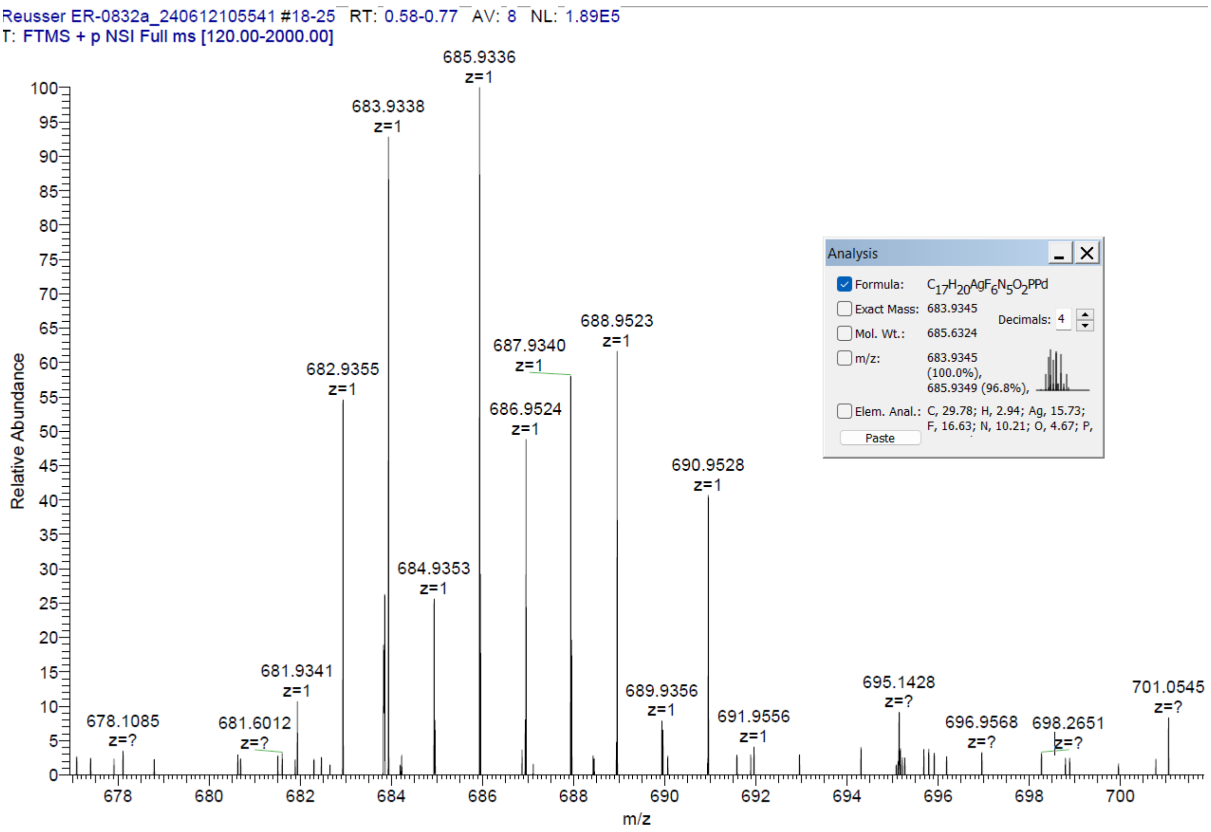

Figure S42: HR-MS in the M^+ region (677–702 amu) of **5a** and calculated isotope pattern (inset).

S.1.4.3 Synthesis of complex 5d

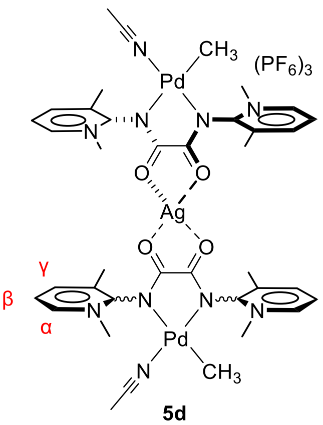

5d

1:1 rotamers ratio

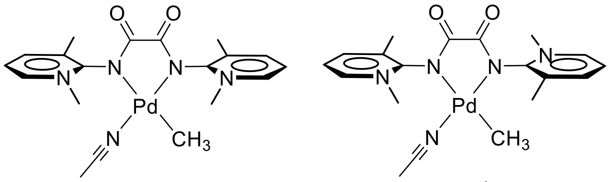

syn *anti*

Compound **2d** (50 mg, 85 µmol), silver carbonate (25 mg, 89 µmol) and [PdMeCl(cod)] (23 mg, 85 µmol) were charged in a 10 mL vial, and the atmosphere was changed to N_2. Dry MeCN (2 mL) was added, and the resulting suspension was stirred at 23 °C for 90 min. The reaction mixture was then filtered through a plug of celite. More MeCN (2 mL) was used to wash the celite. The combined MeCN filtrates were concentrated under vacuum and triturated in pentane. The residue was recrystallized upon Et_2O addition to a mixture of MeCN/CH_2Cl_2 and dried under vacuum to yield **5d** as a pale brown solid (38 mg, 61%). NMR analysis revealed the presence of two rotamers in a 1:1 ratio, presumably the *syn* and *anti*-configuration. Distinctive shift between the two rotamers are only visible for aliphatic protons and carbon resonances. Note: for the NMR characterization *cis*- refers to the PYA unit adjacent to the -CH_3 ligand. ^{1}H NMR (400 MHz, DMSO–*d*$_6$, 298K) δ = 8.39 (d, $^3J_{HH}$ = 6.2 Hz, 2H, *cisα*-C*H*), 8.31 (d, $^3J_{HH}$ = 6.2 Hz, 2H, *transα*-C*H*), 8.27 (d, $^3J_{HH}$ = 7.7 Hz, 2H, *cisγ*-C*H*), 8.18 (d, $^3J_{HH}$ = 7.6 Hz, 2H, *transγ*-C*H*), 7.56 (dd, $^3J_{HH}$ = 7.7, 6.2 Hz, 2H, *cisβ*-C*H*), 7.44 (dd, $^3J_{HH}$ = 7.6, 6.2 Hz, 2H, *transβ*-C*H*), 4.21, 4.20 (2 x s, 3H, *trans*-NC*H*$_3$), 4.16, 4.14 (2 x s, 3H, *cis*-NC*H*$_3$), 2.53, 2.51 (2 x s, 3H, *trans*-CC*H*$_3$), 2.46 & 2.43 (s, 3H + 3H, *cis*-CC*H*$_3$), 1.96 ppm (s, 6H, CH_3CN), –0.26, –0.27 (2 x s, 3H, Pd-C*H*$_3$) ppm. Free is detected due to the exchange with CD_3CN. ^{13}C{^{1}H} NMR (101 MHz, CD_3CN, 298K) δ = 165.89 (*trans*-*C*O or *cis*-CO), 162.05 (*trans*-*C*O or *cis*-CO), 160.50 (*trans*-*C*$_{PYA}$), 159.08 (*cis*-*C*$_{PYA}$), 147.25 & 147.20 (*cis*γ-*C*H), 146.37 (*trans*γ-*C*H), 143.07 (*cis*α-*C*H), 142.50 (*trans*α-*C*H), 137.48 (*cis*-*C*CH$_3$), 135.94 & 135.91 (*trans*-*C*CH$_3$), 123.32 (*cisβ*-*C*H), 121.66 (*transβ*-*C*H), 44.77 & 44.72 & 44.68 (*cis*-N*C*H$_3$ or *trans*-N*C*H$_3$), 18.48 & 18.42 (*trans*-C*C*H$_3$), 18.14 & 18.04 (*cis*-C*C*H$_3$), -7.23 (Pd-*C*H$_3$). ^{19}F{^{1}H} NMR (376 MHz, CD_3CN, 298 K): -72.91 (d, $^1J_{FP}$ = 706.4 Hz) ppm. ^{31}P{^{1}H} NMR (162 MHz, CD_3CN) δ -144.61 (septet, $^1J_{PF}$ = 706.4 Hz) ppm. Quantitative ^{19}F{^{1}H} NMR (282 MHz, CD_3CN, 298 K): using 1,2,4,5-tetrafluorobenzene as fluorinated standard (*cf.* Figures S1, S2) revealed a 1.42/1 ratio of PF_6 *vs* ligand unit by comparing ^{1}H NMR and ^{19}F NMR integrations. HR-ESI-MS (m/z): calculated for $C_{19}H_{24}AgF_6N_5O_2PPd$ [M-PF_6]$^+$ = 713.9657; found: 713.9669. Elemental analysis calculated for $C_{38}H_{48}A_gF_{18}N_{10}O_4P_3Pd_2$ (%): C 31.17; H 3.30; N 9.56, found: C 30.89; H 3.24; N 9.05.

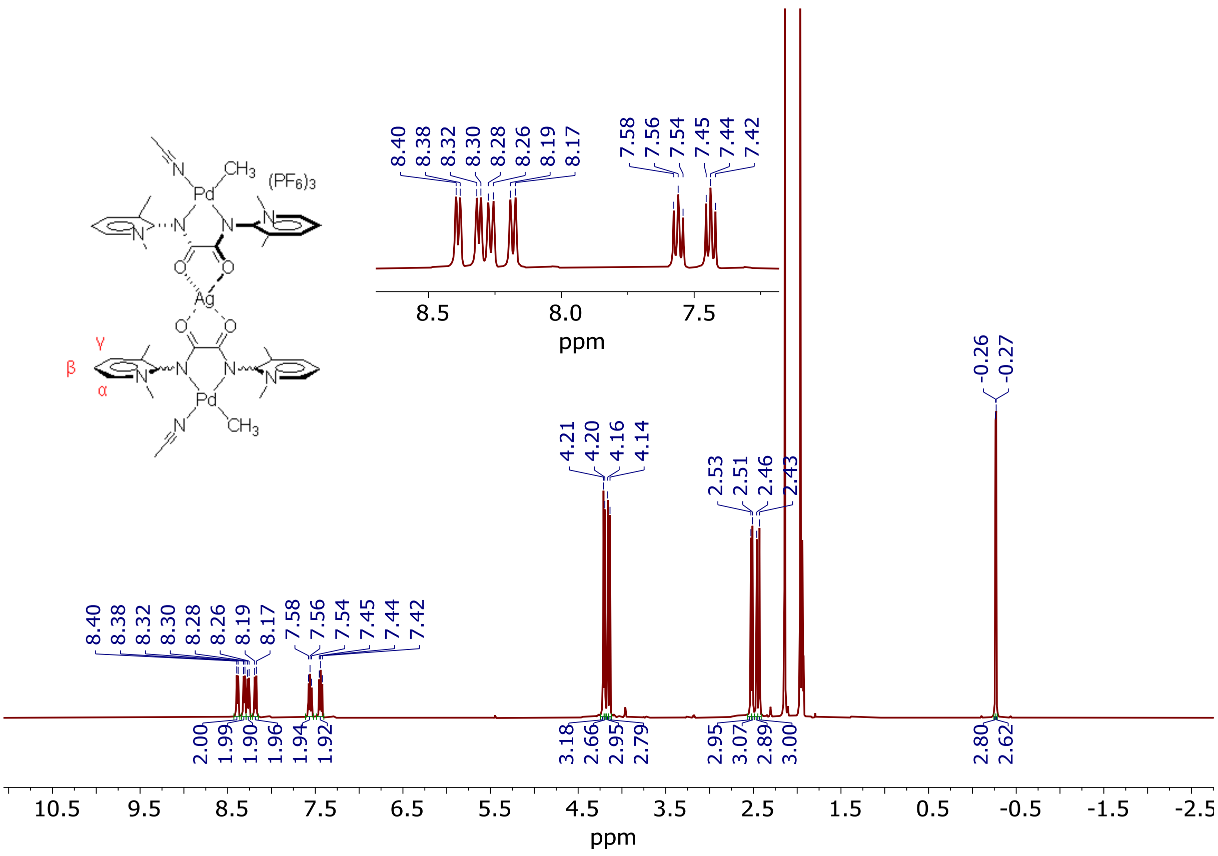

Figure S43: ^{1}H NMR spectrum (CD_3CN, 298 K, 400 MHz) of **5d.** Inset: zoom on the aromatic region.

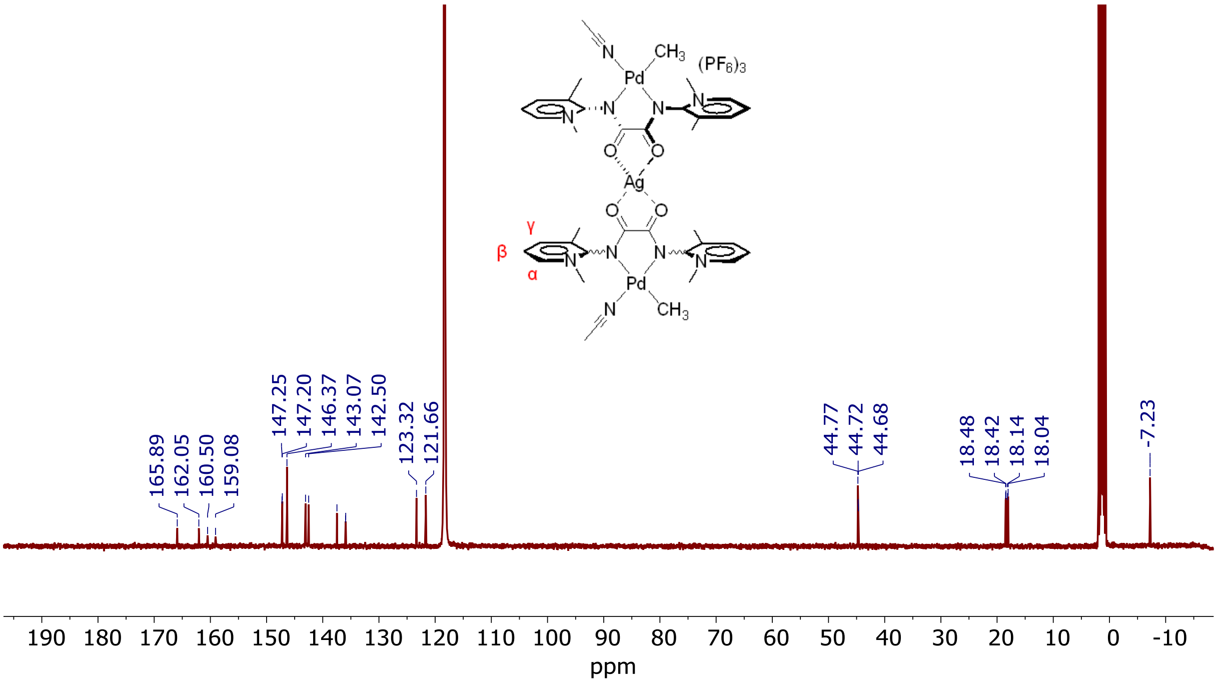

Figure S44: $^{13}C\{^{1}H\}$ NMR spectrum (CD_3CN 298 K, 101 MHz) of **5d**

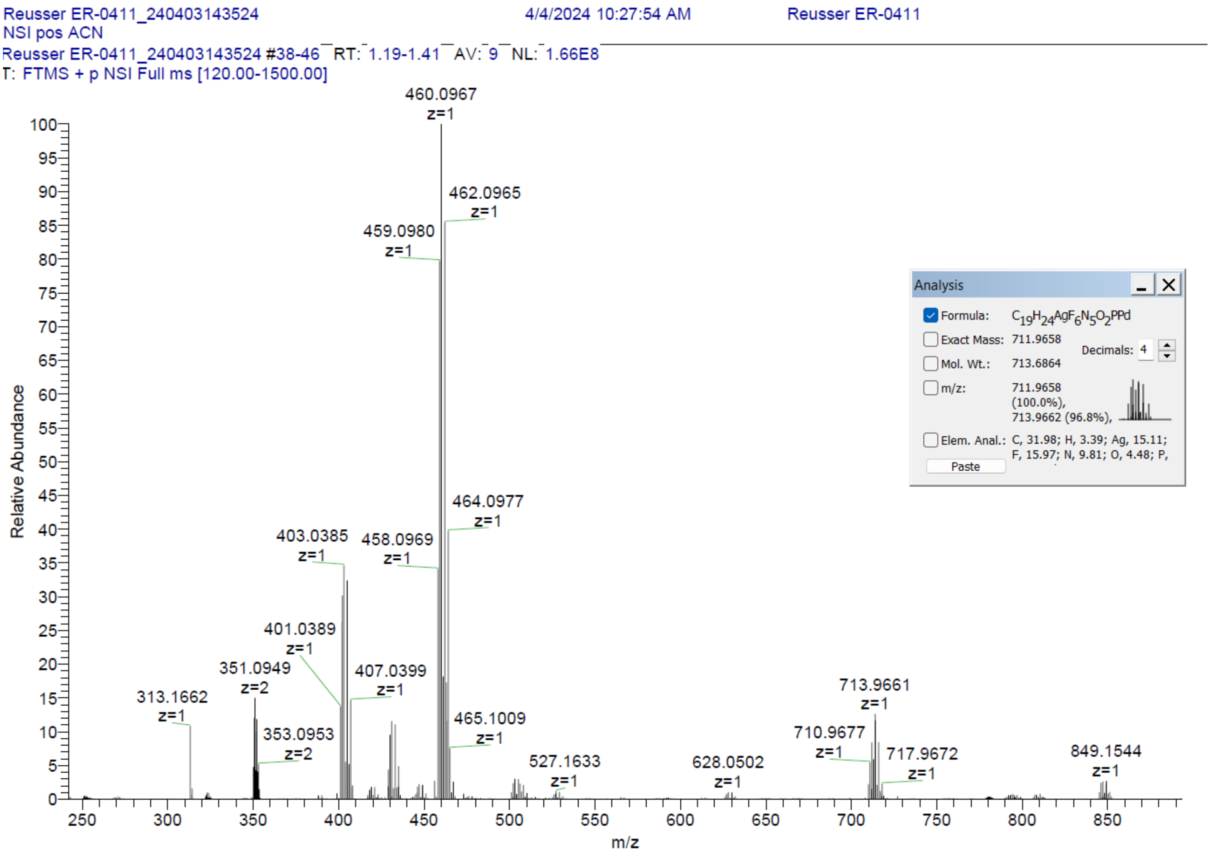

Figure S45: HR-MS of **5d** and calculated isotope pattern for M^+ (inset).

S.1.4.2 Synthesis of complex 6a

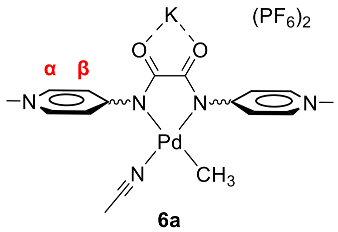

6a

Compound **2a** (112 mg, 200 µmol), potassium carbonate (29.0 mg, 210 µmol) and [PdMeCl(cod)] (53 mg, 200 µmol) were charged in a 5 mL vial, and the atmosphere was changed to N_2. Dry MeCN (2 mL) was added, and the resulting suspension was stirred at 23 °C for 1 h. The reaction mixture was directly filtered through a plug of celite and extracted with MeCN (5 mL). The combined MeCN fractions were concentrated under vacuum, triturated in CH_2Cl_2 (2 x 5 mL), pentane (5 mL), and dried under vacuum to yield **6a** as a yellow solid (101 mg, 75%). ^{1}H NMR (400 MHz, DMSO–*d*$_6$, 298K) δ = 8.20–8.04 (m, 4H, α-C*H*), 7.96–7.77 (m, 4H, β-C*H*), 4.02, 4.00 (2 x s, 3H, NC*H*$_3$), 1.96 (s, 6H, C*H*$_3$CN), 0.29 (s, 3H, Pd-C*H*$_3$) ppm. ^{13}C{^{1}H} NMR (101 MHz, DMSO-*d*$_6$, 298K) δ = 169.31 (*C*O), 168.75 (*C*O), 164.11 (*C*$_{PYA}$), 163.88 (*C*$_{PYA}$), 144.53 (α-*C*H), 144.38 (α-*C*H), 125.14 (β-*C*H), 121.83 (β-*C*H), 47.141 N*C*H$_3$), 46.66 N*C*H$_3$), -2.19 (Pd-*C*H$_3$). ^{19}F{^{1}H} NMR (376 MHz, CD_3CN, 298 K): -72.88 (d, $^1J_{FP}$ = 706.7 Hz) ppm. ^{31}P{^{1}H} NMR (162 MHz, CD_3CN, 298 K): -144.61 (septet, $^1J_{PF}$ = 706.7) ppm. Quantitative ^{19}F{^{1}H} NMR (282 MHz, CD_3CN, 298 K): using 1,2,4,5-tetrafluorobenzene as fluorinated standard (*cf* Figures S1, S2) revealed a 2.16/1 ratio of PF_6 *vs* ligand unit by comparing ^{1}H NMR and ^{19}F NMR integrations. This ratio did not vary upon further purification. HR-ESI-MS (m/z): calculated for $C_{17}H_{20}F_6KN_5O_2PPd$ [M-PF$_6$]$^+$ = 615.9931; found: 615.9922. Despite several purification attempts, no satisfying elemental analysis was obtained. The closest match includes

residual KPF_6: elemental analysis calculated for $C_{17}H_{20}F_{12}KN_5O_2P_2Pd$ x 0.1 KPF_6 (%): C 26.17; H 2.58; N 8.98, found: C 26.14; H 2.46; N 8.24.

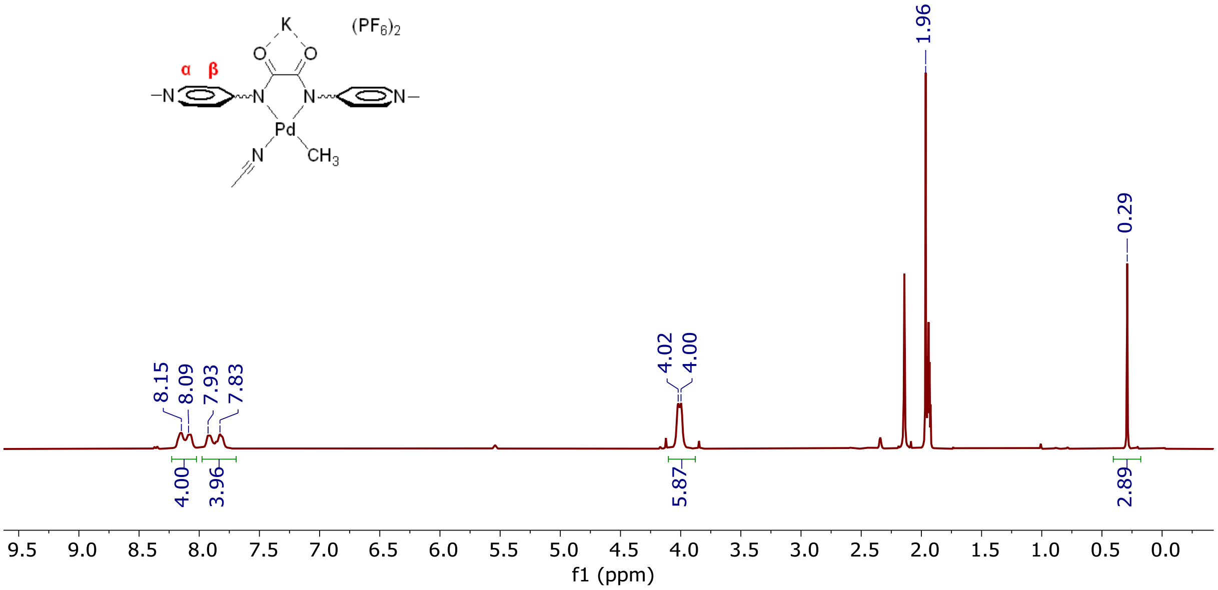

Figure S46: ^{1}H NMR spectrum (CD_3CN, 298 K, 400 MHz) of **6a**.

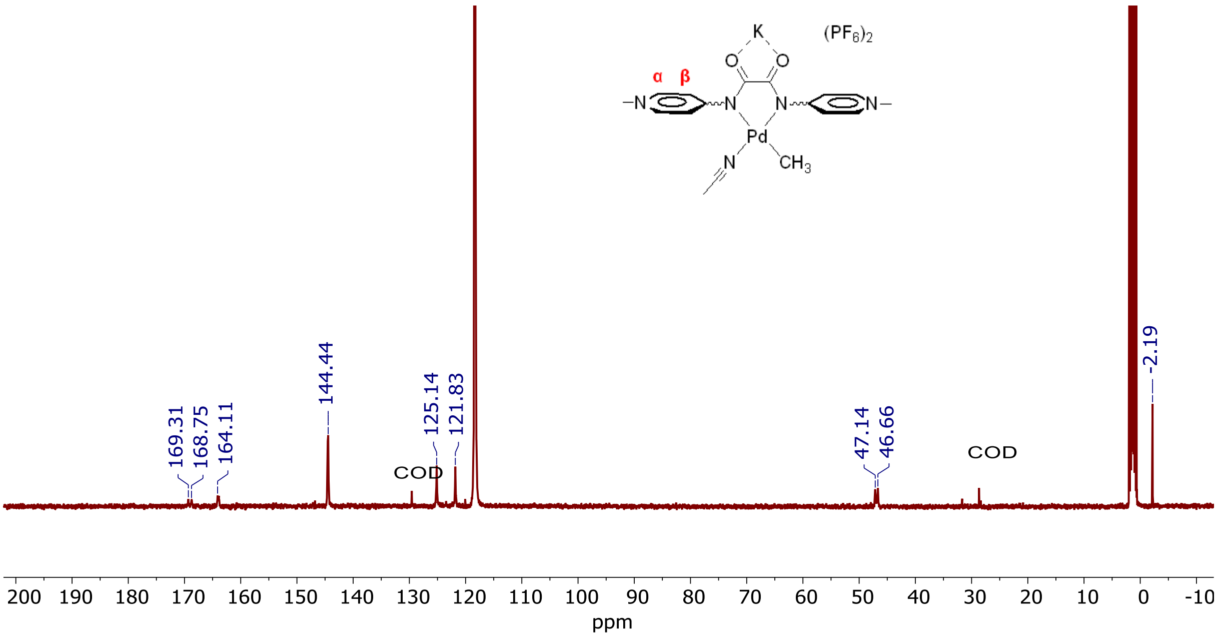

Figure S47: $^{13}C\{^1H\}$ NMR spectrum (CD_3CN 298 K, 101 MHz) of **6a**

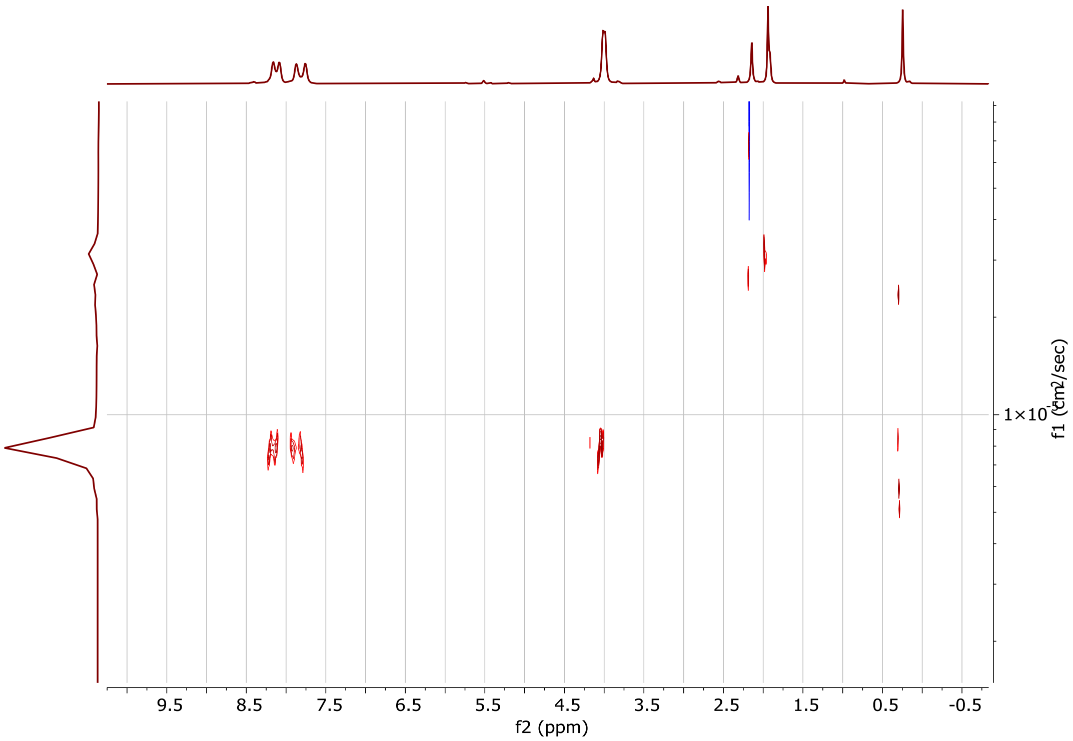

Figure S48: ¹H DOSY NMR spectrum (CD_3CN, 298 K, 400 MHz) of **6a**. All the signals corresponding to the NCH_3 have similar diffusion coefficient ~8.0 x 10^{-6} cm^2/s, corresponding to an approximate molecular weight of ~1250 g/mol.[S11–S13] This molecular weight exceeds the expected weight for 1:1 K:Pd complex adduct (762 g/mol with ion pairing) and might indicate more complex interactions in solution.

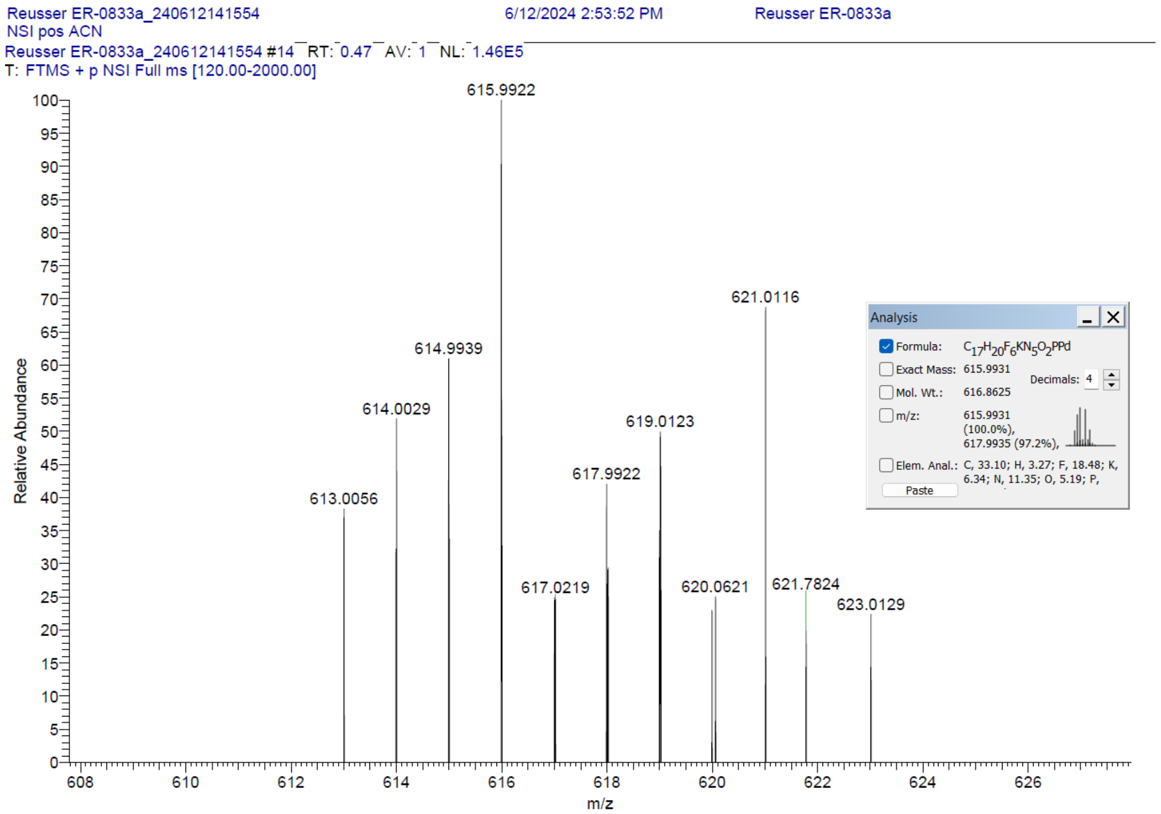

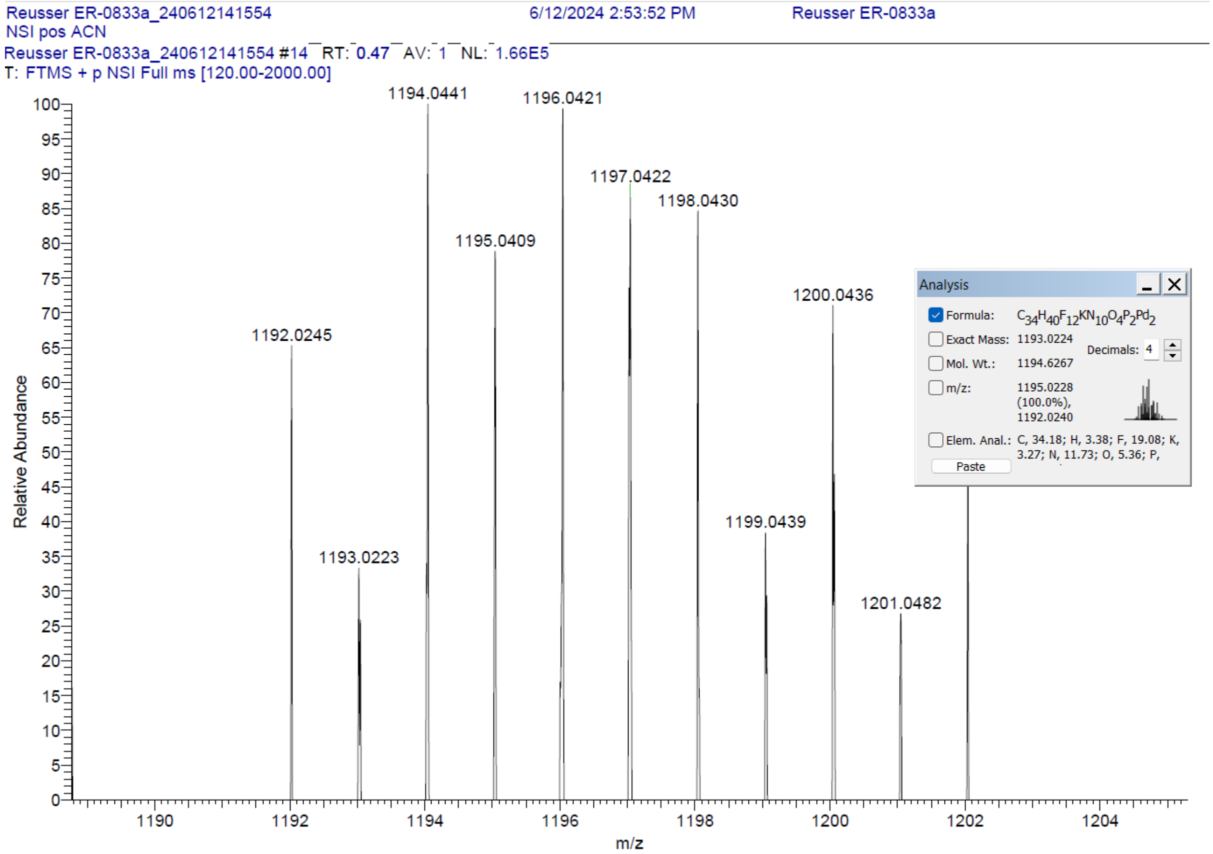

Figure S49: HR-MS isotope pattern of M^+ of **6a** and calculated isotope pattern for M^+ (inset) in bridged from (bottom) and monoligated (top).

S.1.4.4 Synthesis of complex 6d

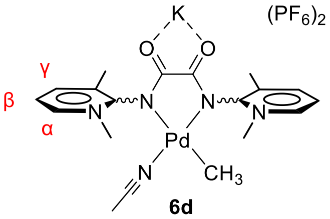

6d

1:1 rotamers ratio

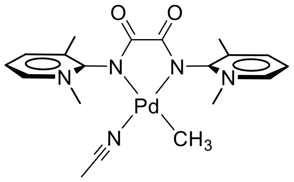

syn

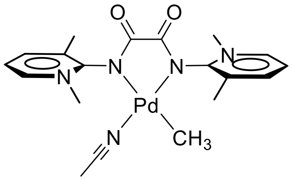

anti

Compound **2d** (118 mg, 200 μmol), potassium carbonate (29 mg, 210 μmol) and [PdMeCl(cod)] (53 mg, 200 μmol) were charged in a 5 mL vial, and the atmosphere was changed to N_2. Dry MeCN (2 mL) was added, and the resulting suspension was stirred at 23 °C for 1 h. The reaction mixture was directly filtered through a plug of celite and the residue extracted with copious amounts of MeCN (5 mL). After concentration of the MeCN fractions under vacuum, the residue was triturated in CH_2Cl_2 (2 x 5 mL), pentane (5 mL), and dried under vacuum to yield **6d** as a yellow solid (150 mg, 97%). Crystals suitable for single crystal XRD analysis were grown by slow diffusion of Et_2O in a MeCN solution of **6d**. NMR analysis revealed the presence of two rotamers in a 1:1 ratio, presumably the *syn* and *anti*-configuration. Distinctive chemical shifts between the two rotamers are only visible for aliphatic protons and carbon resonances. Note: for the NMR characterization *cis*- refers to the PYA unit adjacent to the -CH_3 ligand. ^{1}H NMR (400 MHz, DMSO–d_6, 298K) δ = 8.40 (d, $^3J_{HH}$ = 5.9 Hz, 2H, *cisα*-C*H*), 8.32 (d, $^3J_{HH}$ = 6.4 Hz, 2H, *transα*-C*H*), 8.27 (d, $^3J_{HH}$ = 7.7 Hz, 2H, *cisγ*-C*H*), 8.19 (d, $^3J_{HH}$ = 7.5 Hz, 2H, *transγ*-C*H*), 7.57 (dd, $^3J_{HH}$ = 7.7, 5.7 Hz, 2H, *cisβ*-C*H*), 7.45 (dd, $^3J_{HH}$ = 7.5, 6.4 Hz, 2H, *transβ*-C*H*), 4.21, 4.19 (2 x s, 3H, *trans*-NC*H*3), 4.16, 4.13 (2 x s, 3H, *cis*-NC*H*3), 2.52, 2.51 (2 x s, 3H, *trans*-CC*H*3), 2.46, 2.43 (2 x s, 3H, *cis*-CC*H*3), 1.96 (s, 6H, MeCN), –0.25, –0.26 (2 x s, 3H, Pd-C*H*3) ppm. ^{13}C{^{1}H} NMR (101 MHz, DMSO–d_6, 298K) δ = 166.29 (*trans*-*C*O or *cis*-CO), 162.38 (*cis*-*C*O or *trans*-CO), 160.22 (*trans*-C_{PYA}), 158.80 (*cis*-C_{PYA}), 147.35, 147.29 (2 x *cis*γ-*C*H), 146.48 (*trans*γ-*C*H), 143.13 (*cis*α-*C*H), 142.58 (*trans*α-*C*H), 137.41 (*cis*-*C*CH3), 135.88, 135.84 (2 x *trans*-*C*CH3), 123.46 (*cisβ*-*C*H), 121.82, 121.80 (2 x d, *transβ*-*C*H), 44.78, 44.73, 44.69 (3 x *cis*-N*C*H3 or *trans*-N*C*H3), 18.43, 18.37 (2 x *trans*-C*C*H3), 18.10, 18.00 (2 x *cis*-C*C*H3), –7.06 (Pd–*C*H3). ^{19}F{^{1}H} NMR (376 MHz, CD_3CN, 298 K): -72.90 (d, $^1J_{FP}$ = 706.6 Hz) ppm. ^{31}P{^{1}H} NMR (162 MHz, CD_3CN) δ -144.61 (septet, $^1J_{PF}$ = 706.3 Hz) ppm. Quantitative ^{19}F{^{1}H} NMR (282 MHz, CD_3CN, 298 K): using 1,2,4,5-tetrafluorobenzene as fluorinated standard revealed a 2.14/1 ratio of PF_6 vs ligand unit by comparing ^{1}H NMR and ^{19}F NMR integrations (*cf* Figures S1, S2). HR-ESI-MS (m/z): calculated for $C_{19}H_{24}F_6KN_5O_2PPd$ [M-PF_6]$^+$ = 644.0244; found: 644.0241. Despite several purification attempts, no satisfying elemental analysis wase obtained with the closest match including some residual KPF_6. Elemental analysis calculated for $C_{19}H_{24}F_{12}KN_5O_2P_2Pd$ x 0.2 (KPF_6) (%): C 27.89; H 2.85; N 8.11, found: C 27.61; H 2.93; N 8.47.

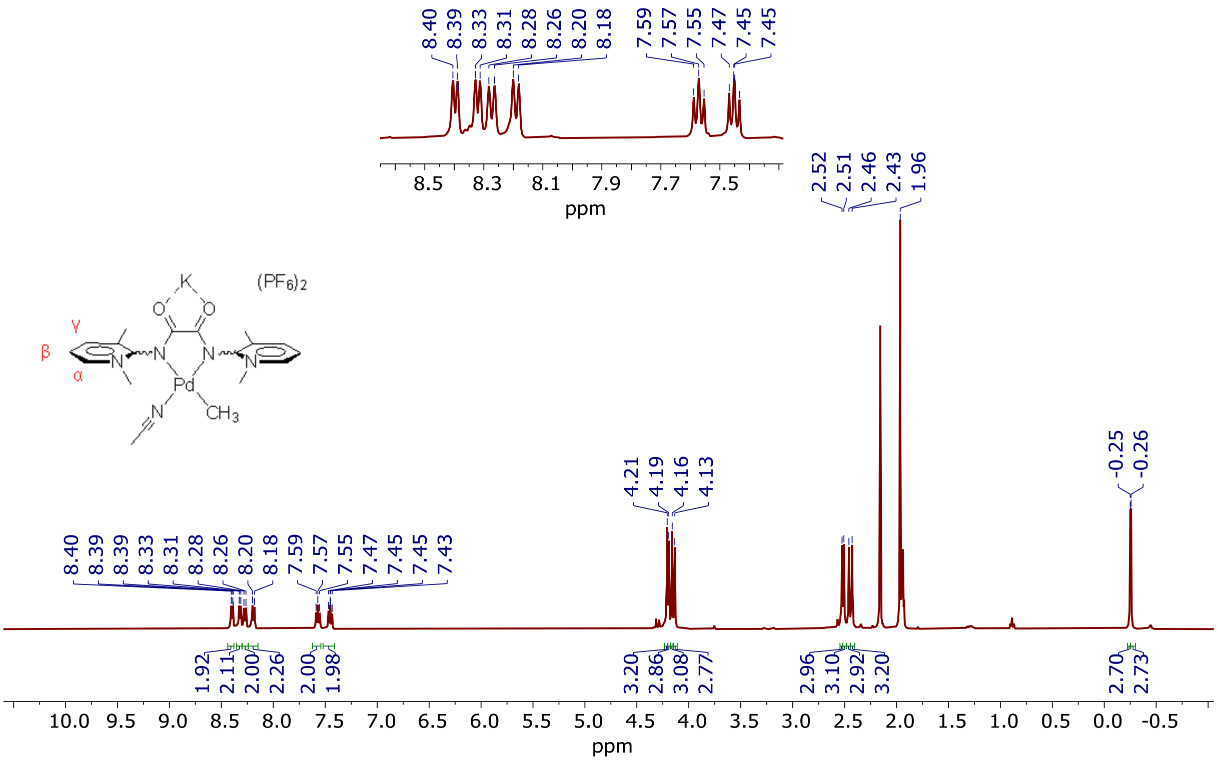

Figure S50: ^{1}H NMR spectrum (CD_3CN, 298 K, 400 MHz) of **6d**. Inset: zoom on the aromatic region.

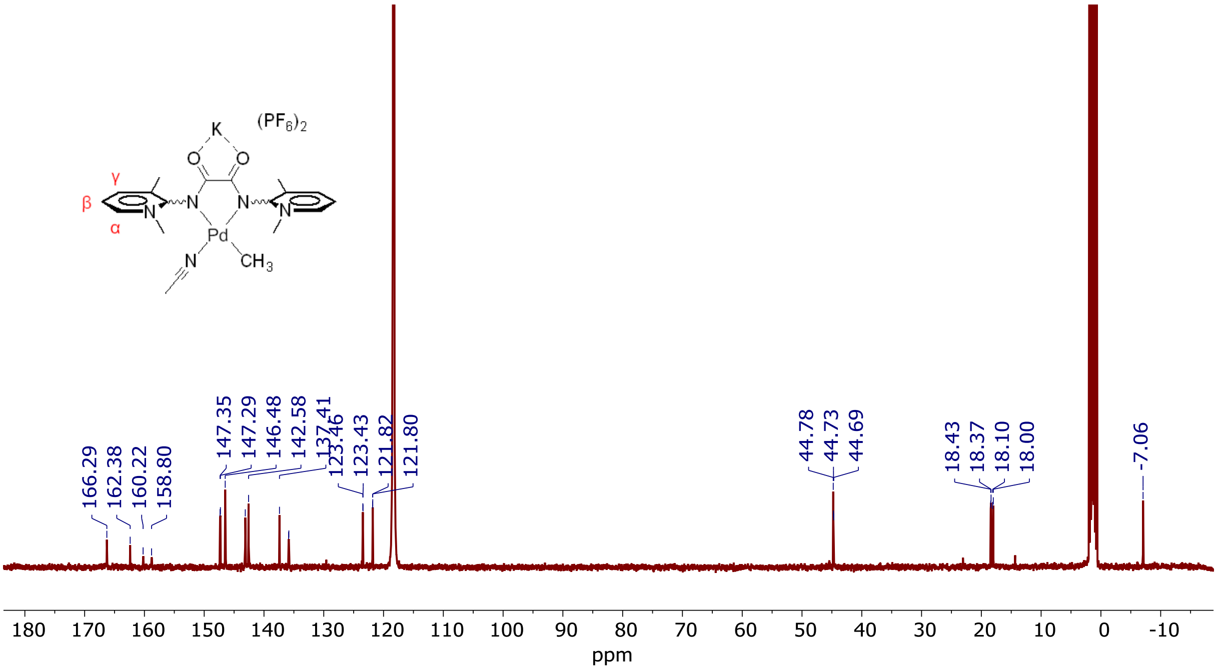

Figure S51: $^{13}C\{^{1}H\}$ NMR spectrum (CD_3CN 298 K, 101 MHz) of **6d**.

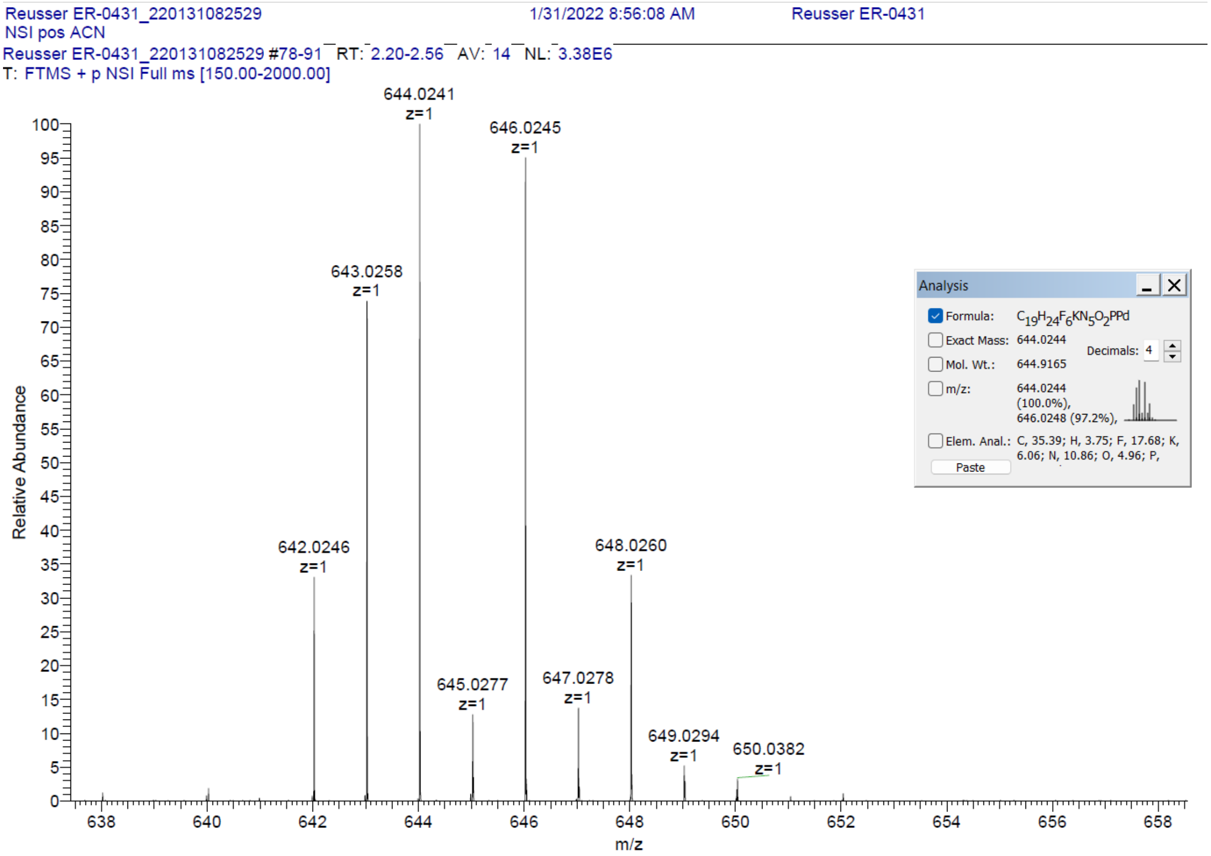

Figure S52: HR-MS isotope pattern of M^+ of **6d** and calculated isotope pattern for M^+ (inset).

S.2. Reactivity with olefins

Typical procedure for ethylene conversion: The Pd catalyst (10 µmol), the halide abstractor (11 or 25 µmol) and durene (6.9 mg, 50 µmol) were placed in a screw-capped NMR tube. Dry CD_2Cl_2 (0.5 mL) was added, and the resulting suspension was saturated with ethylene at atmospheric pressure for 60 seconds. Then the tube was quickly capped and spun on a mechanical spinner for the appropriate time at 23 °C. Conversion was followed by ^{1}H NMR spectroscopy. At the end of the reaction, the reaction mixture was filtered through celite and analysed by GC-EI-MS.

Typical procedure for polymerization: A 25 mL round-bottom flask was loaded with the Pd catalyst (10 µmol), NaBArF (25 µmol, 22.2 mg), and a stir bar and weighed. The flask was subsequently evacuated and backfilled twice with nitrogen, followed by a final backfill with ethylene from a balloon to establish an ethylene atmosphere. Anhydrous 1,2-dichloroethane (10 mL) was added, and the reaction mixture was stirred vigorously at 23 °C for 24 hours. To ensure continuous ethylene supply, the balloon's outlet needle remained immersed in the solution. Additional ethylene was introduced by

attaching supplementary balloons when the initial balloon visibly deflated, indicating significant gas consumption.

After 24 hours, a 0.1 mL aliquot of the solution was removed, dissolved in CD_2Cl_2, and analyzed by ^1H NMR spectroscopy to assess product distribution. The volatiles were evaporated under vacuum, and the residual mass was measured to evaluate productivity. The resulting polymeric wax was dissolved in CD_2Cl_2 and further characterized by ^1H and ^{13}C NMR spectroscopy.

S.3. Characterization of the products

The product distribution was evaluated by one- and two-dimensional NMR spectroscopy as well as by GC-EI-MS when oligomers were formed. Alkyl/vinyl ratios were determined by integrating all aliphatic signals divided by the sum of integrals of all vinylic proton signals. This ratio was used to assess the length of oligomers. A larger alkyl/vinyl ratio indicates the incorporation of more ethylene units in the product. For instance, 2-butene has a 3:1 ratio, while 2-hexene has a 5:1 ratio (Fig. S53). It is also an indirect indicator of the branching degree of an oligomer since trisubstituted alkenes feature less vinylic protons then disubstituted alkenes (Fig. S53). However, the branching degree was determined based on the fact that trisubstituted alkenes display a ^1H NMR resonance around 5.1 ppm, which is slightly upfield compared to the typical shift around 5.3 ppm for disubstituted alkenes.

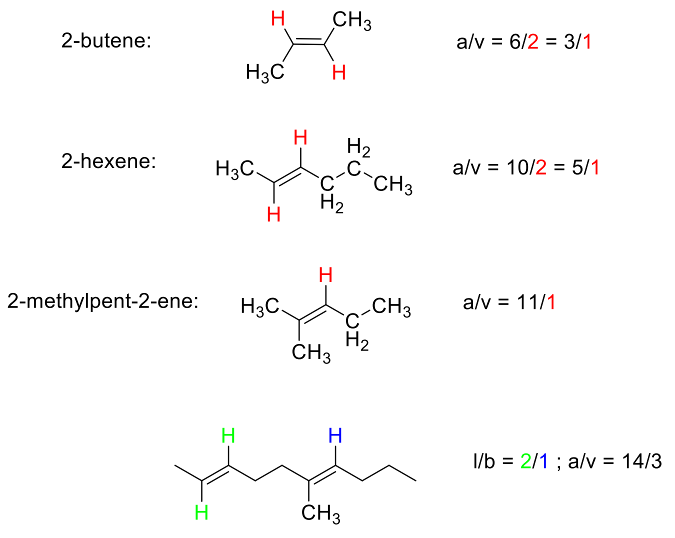

Figure S53: Selected examples for illustrating alkyl/vinyl (a/v) and linear/branched (l/b) ratios. Determination of these ratios is demonstrated for a selective dimerization to butenes (Fig. S54; 5:1 a/v); and a mixture of oligomers (Fig. S55; 31:1 a/v, 4/3 l/b).

Representative evaluation of ethylene conversion by NMR analysis

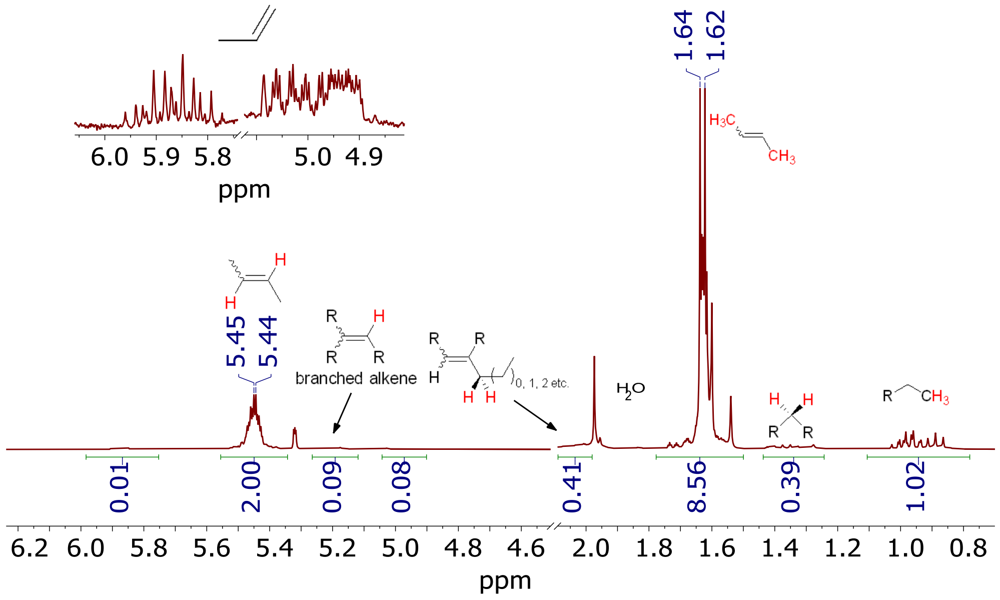

Figure S54: ^{1}H NMR spectrum (CD_2Cl_2, 298 K, 300 MHz) of the crude catalytic mixture obtained by saturating a solution of **4c** and durene as internal standard with ethylene (alkyl/vinyl ratio from integrals as follows alkyl: 0.41 + 8.56 + 0.39 + 1.02 = 10.38; vinyl: 0.01 + 2.00 + 0.09 + 0.10 = 2.20, alkyl/vinyl ratio is 10.38/2.20 = 4.72/1 ≅ 5:1 after 16 h. The NMR spectrum reveals characteristic butene signals as well as traces of residual propene (see inset) and minor traces (0.41) of oligomeric products in the aliphatic region.

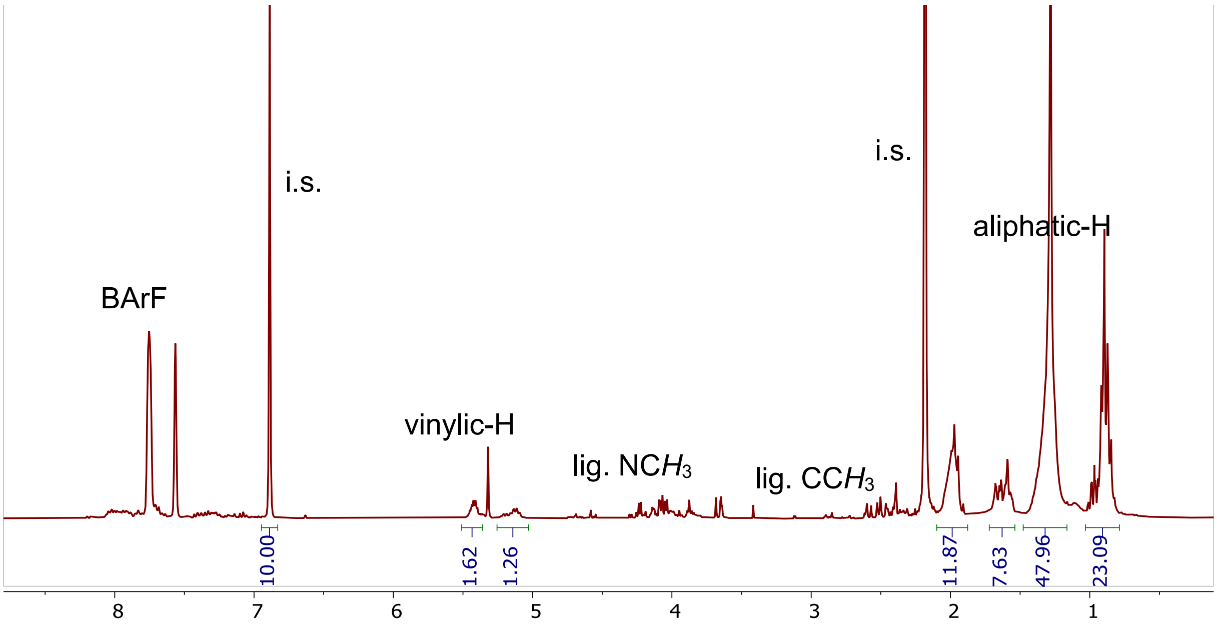

Figure S55: ^{1}H NMR spectrum (CD_2Cl_2, 298 K, 300 MHz) of the crude catalytic mixture obtained by saturating a solution of **4d** and NaBArF (1.1 eq. *vs* Pd.) and 1,3,5-trimethoxybenzene as internal standard (i.s.) with ethylene after 16 h. The alkyl/vinyl ratio here is (11.86 + 7.63 + 47.96 + 23.09) / (1.62 + 1.26) = 90.54/2.88 = 31.44/1 ≅ 31:1. The linear/branched (l/b) ratio is calculated from the vinyl vs internal olefin integrals, here 1.62/1.26 ≅ 4/3. For detailed assignments, see Figure S56.

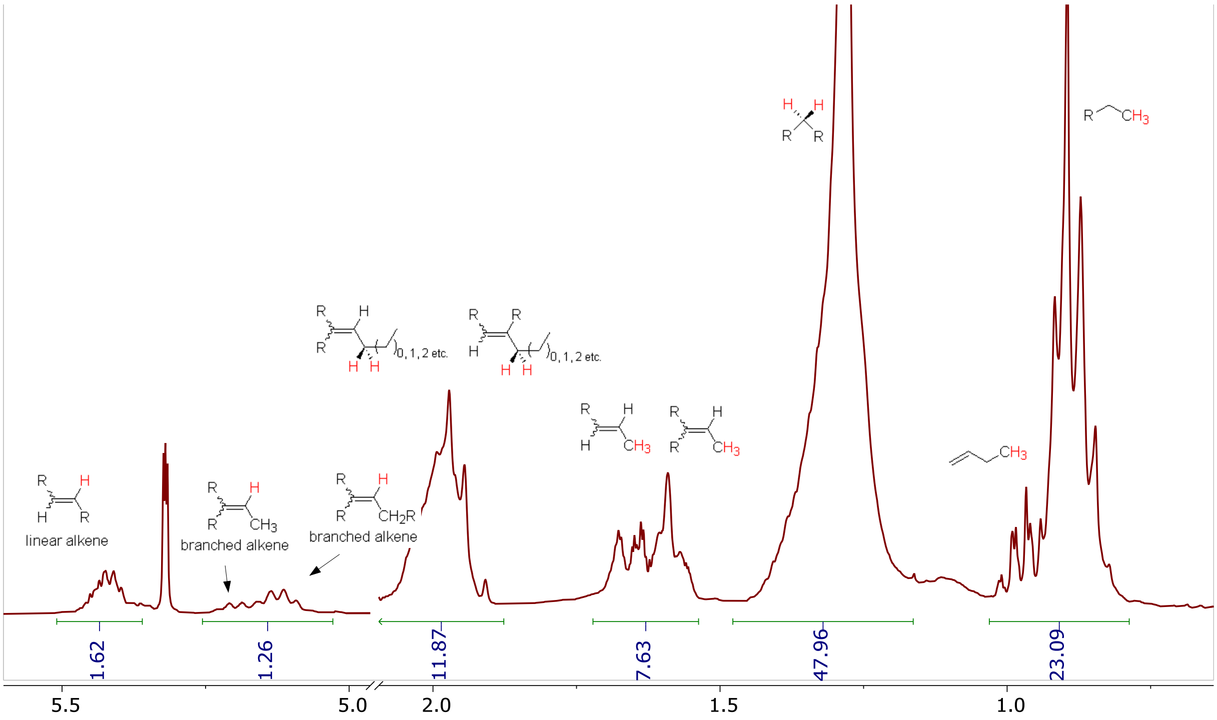

Figure S56: Detailed assignment of the signals to different fragments of olefin products, see Figure S57 for a detailed COSY analysis of a representative reaction mixture (see also refs S13–S15).

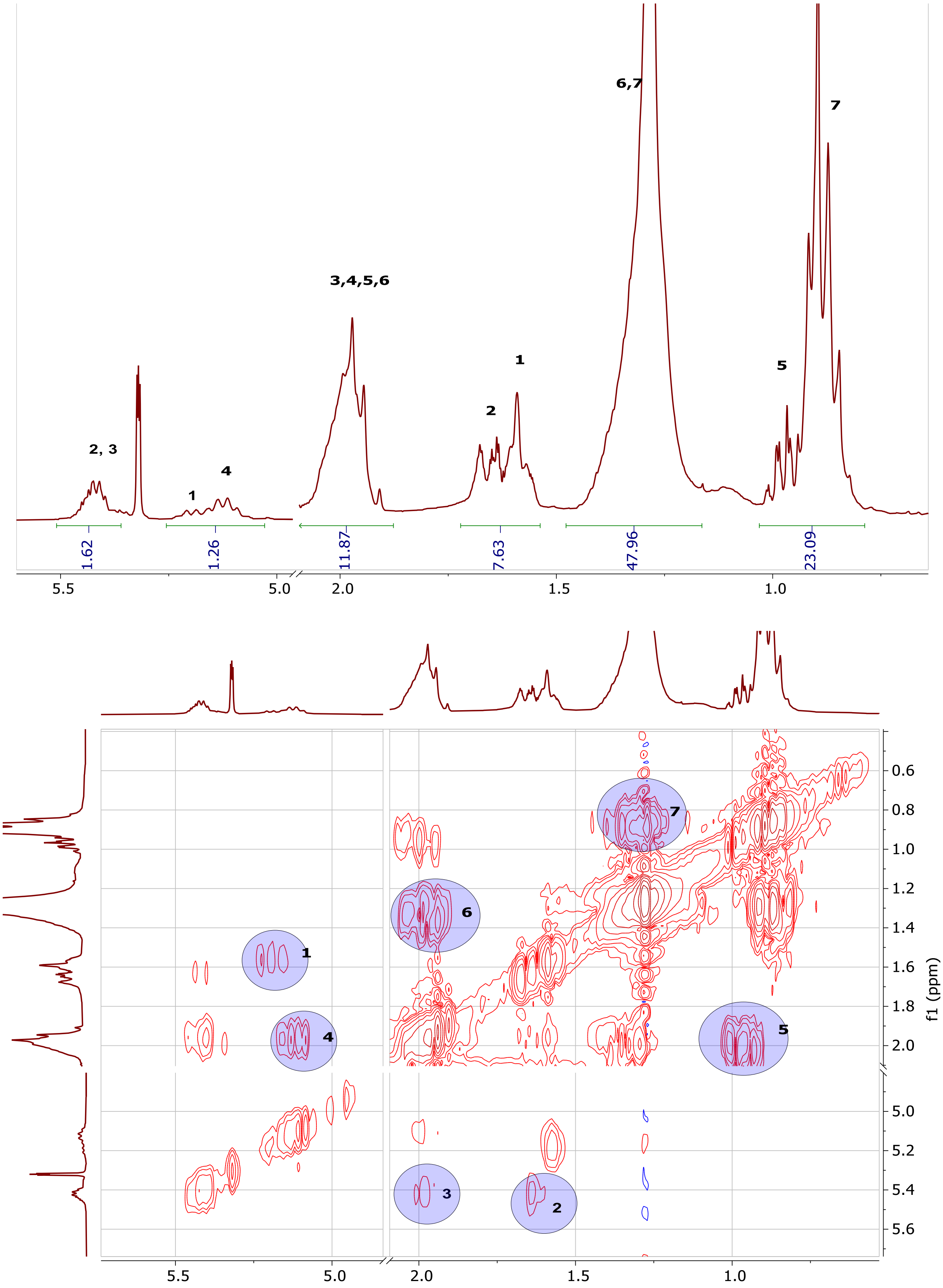

Figure S57: Top shows zoom on vinylic and aliphatic region of the ^{1}H NMR spectrum (CD_2Cl_2, 298 K, 300 MHz) of the crude catalytic mixture obtained by saturating a solution of **4d** and NaBArF (1.1 eq. *vs* Pd) with ethylene (see also Fig. S55, S56); each number corresponds to a COSY interaction between two signals. Bottom shows ^{1}H COSY NMR spectrum (CD_2Cl_2, 298 K, 300 MHz) with the highlighted resonances shown in the NMR spectrum on the top.

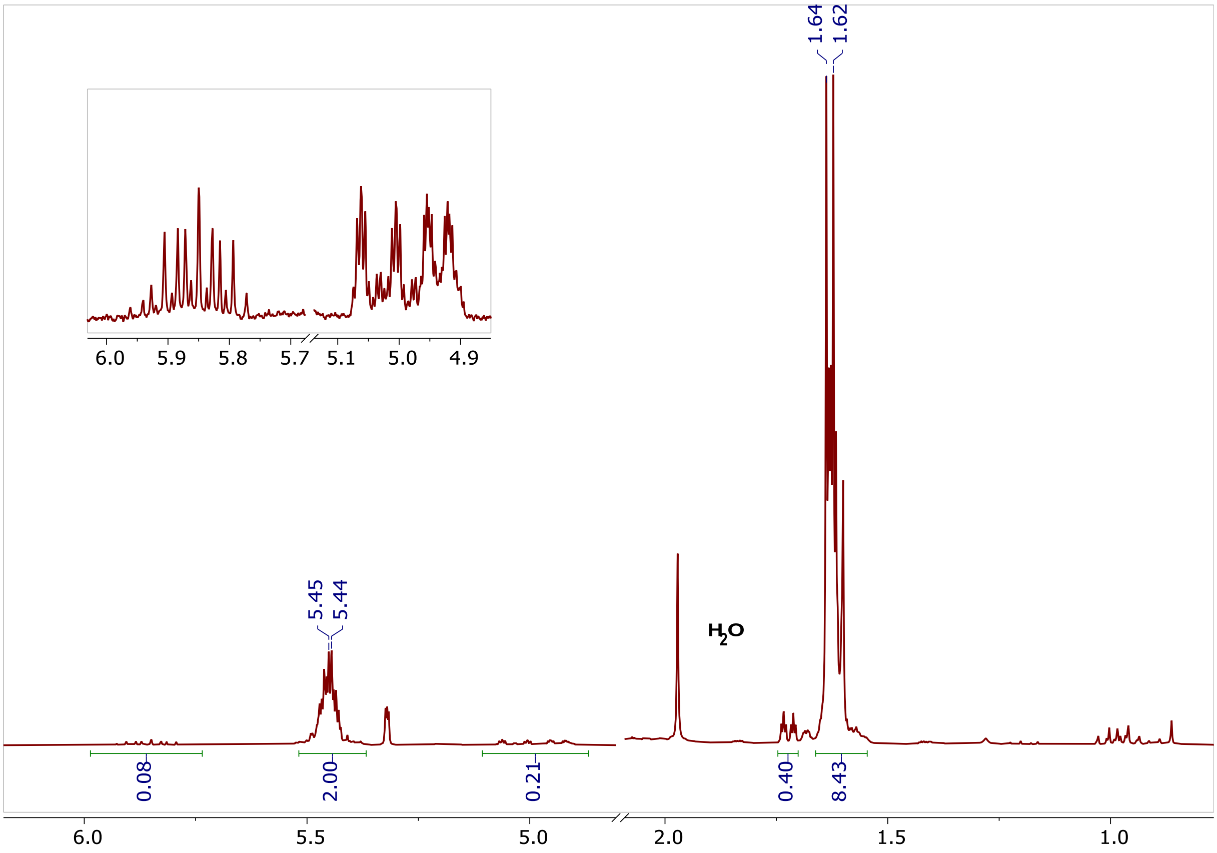

Figure S58: ^{1}H NMR spectrum (CD_2Cl_2, 298 K, 300 MHz) of the crude catalytic mixture obtained by saturating a solution of **4a** and NaBArF (1.1 eq *vs* Pd) with ethylene (alkyl/vinyl ratio = 4/1). The NMR spectrum reveals characteristic butene signals as well as traces of residual propene (see inset) and minor traces of oligomeric products in the aliphatic region.

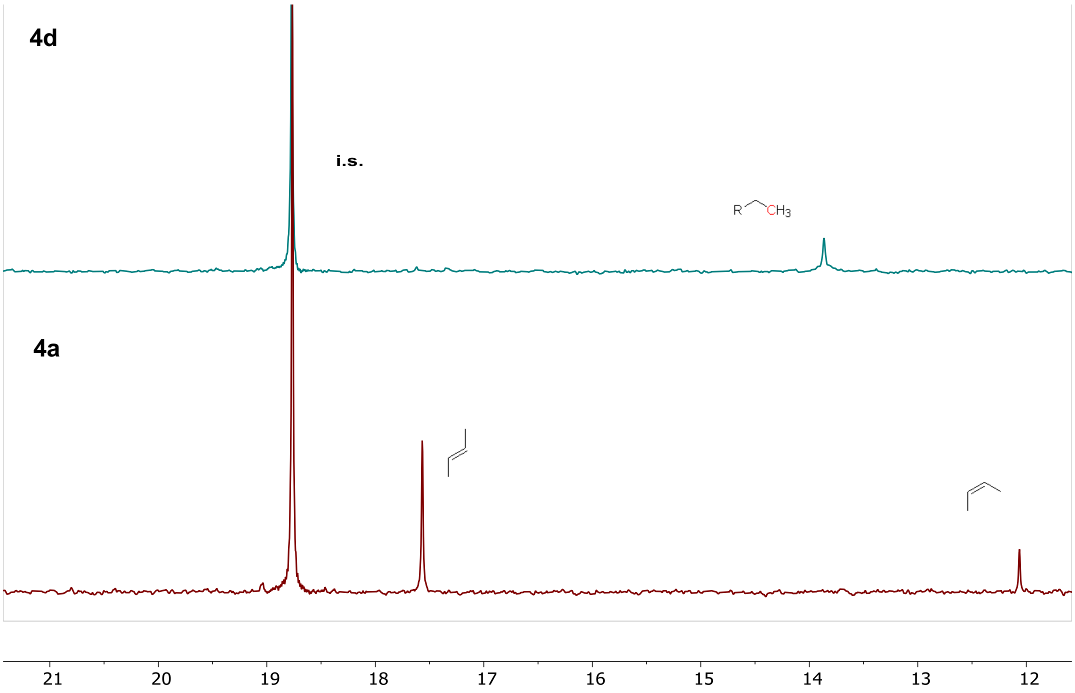

Figure S59: Top: $^{13}C\{^{1}H\}$ NMR spectrum (CD_2Cl_2, 298 K, 101 MHz) of the crude catalytic mixture obtained by saturating a solution of **4d** and 1.1 eq. NaBArF *vs* Pd with ethylene showing full conversion of ethylene and butene; bottom: same spectrum upon using **4a** instead of **4d** showing characteristic butene signals, ^{13}C resonance integration indicates a 4:1 *E/Z* isomeric ratio.

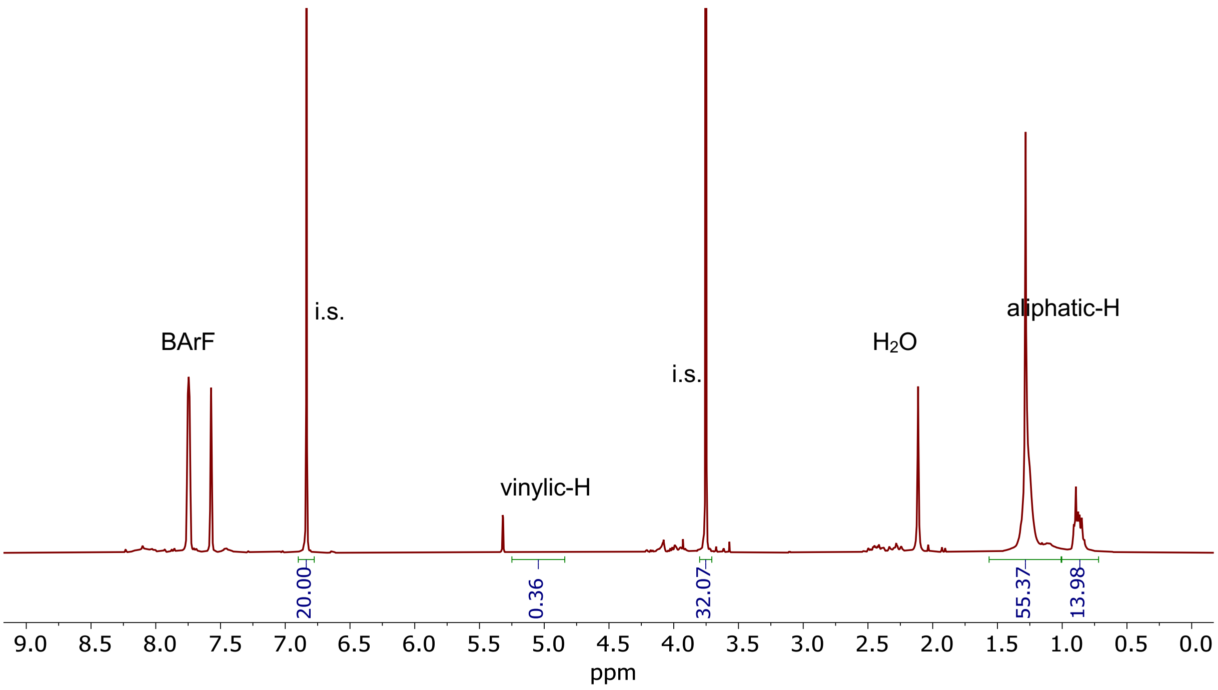

Figure S60: ^{1}H NMR spectrum (CD_2Cl_2, 298 K, 400 MHz) of the crude catalytic mixture obtained by saturating a solution of **4d** and NaBArF (2.5 eq. *vs* Pd) with ethylene: (alkyl/vinyl ratio = 190/1). The alkyl/vinyl ratio decreases after 16 h of spinning, probably due to partial precipitation of the polymeric products in the NMR tube.

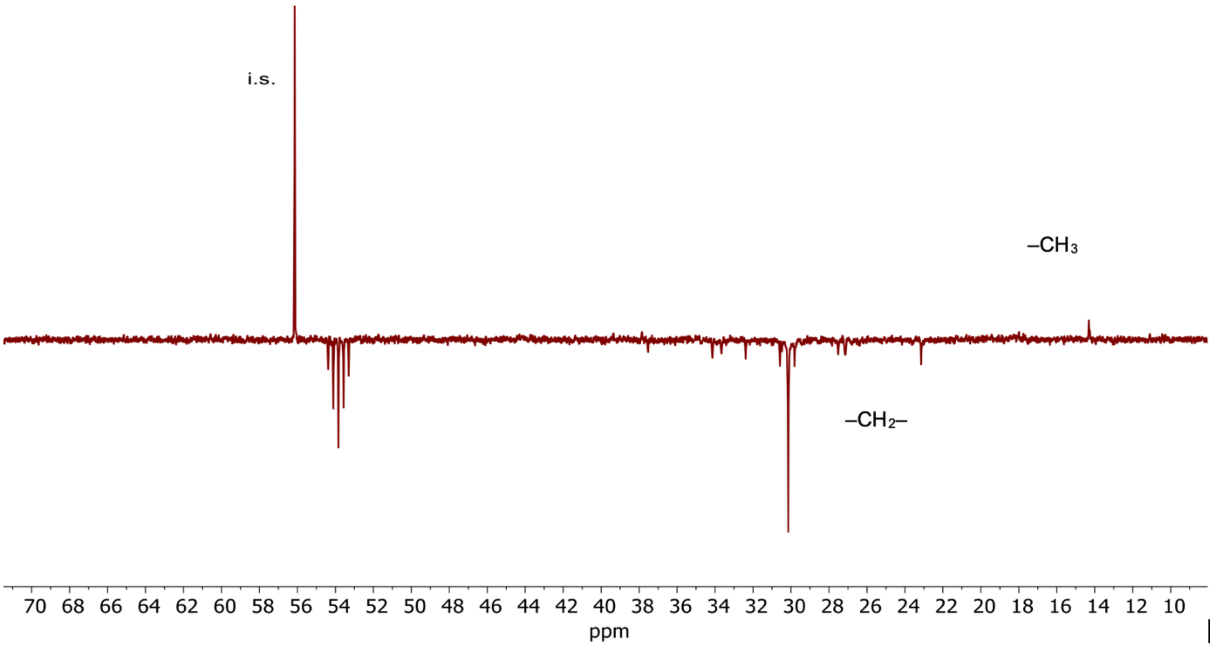

Figure S61: $^{13}C\{^{1}H\}$-APT NMR spectrum (CD_2Cl_2, 298 K, 400 MHz) of the aliphatic region showing the CH_2 signals of the crude catalytic mixture obtained by saturating a solution of **4d** and NaBArF (2.5 eq. *vs* Pd).

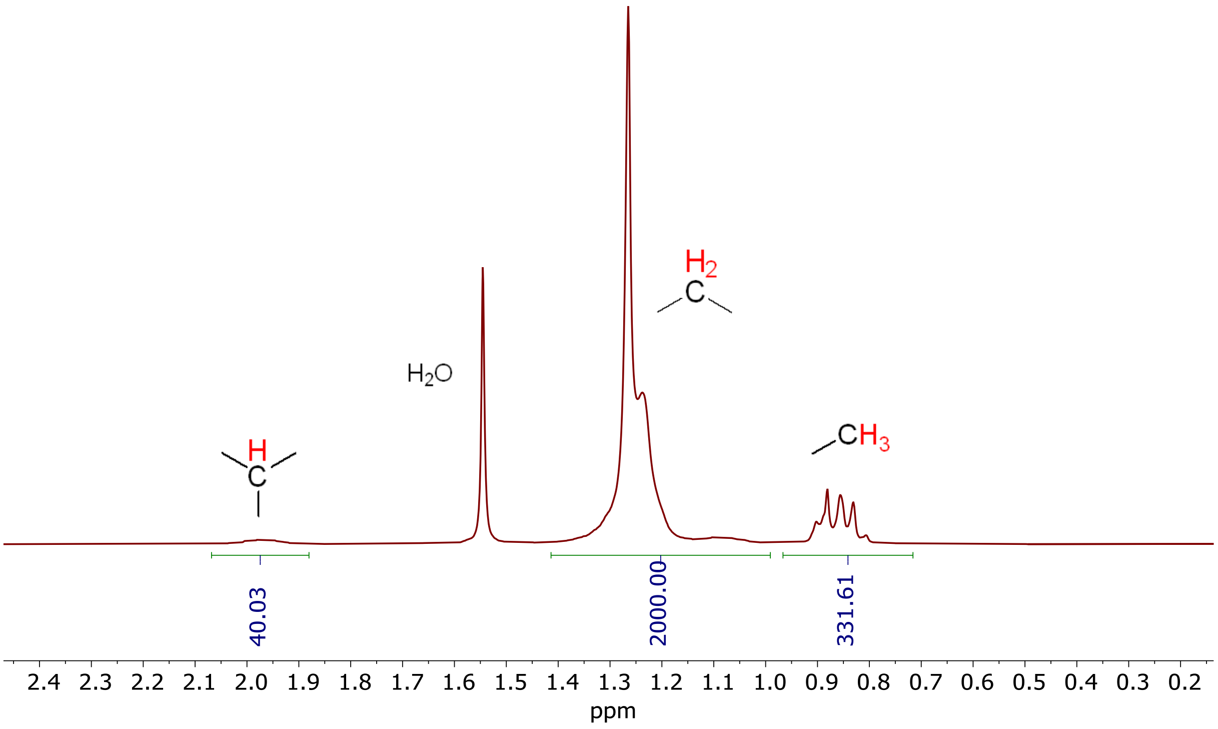

Figure S62: ^1H NMR spectrum (CD_2Cl_2, 298 K, 300 MHz) of the dried polymeric wax obtained by reaction of **4d** and NaBArF (2.5 eq.) and ethylene (1 atm) after 16 h. The respective integration of the -CH_2- and -CH_3 proton resonances were used to estimate the degree of branching (111 branches per 1000 methylene carbons).

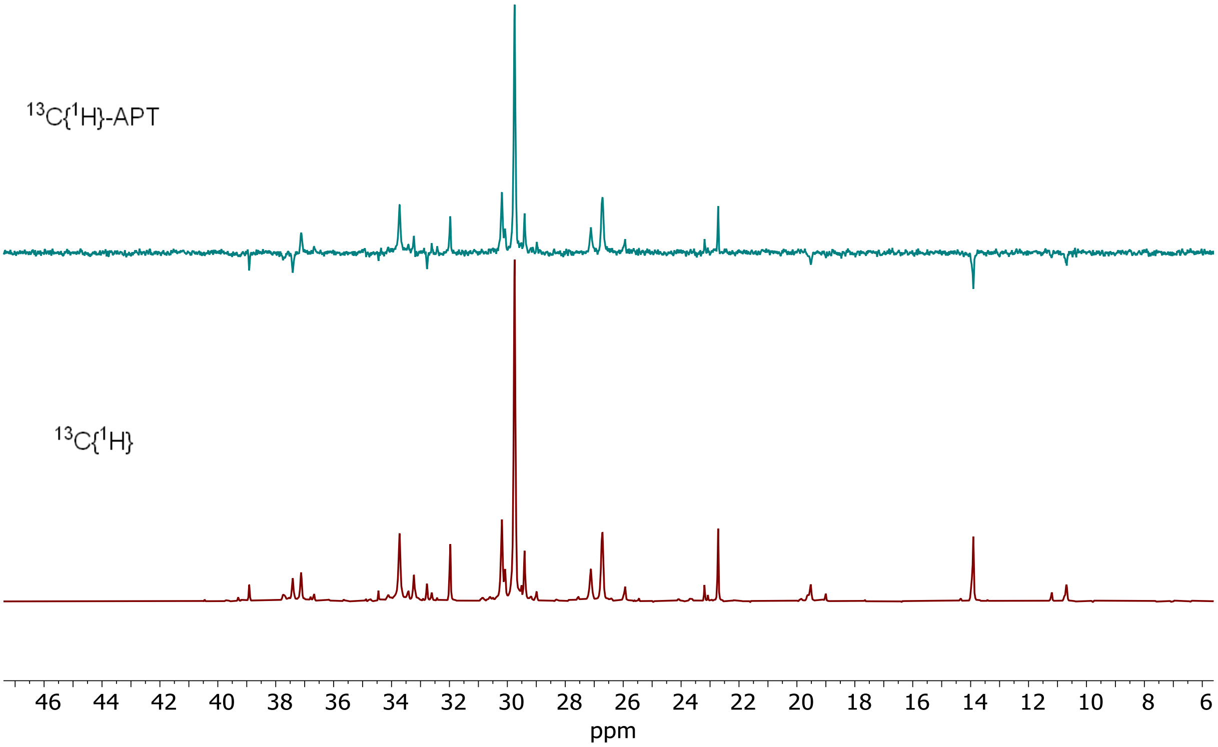

Figure S63: Top $^{13}C\{^1H\}$-APT NMR spectrum (CD_2Cl_2, 298 K, 300 MHz) of the polymeric wax obtained by reacting **4d** and NaBArF 2.5 eq. and ethylene (1 atm). Bottom: $^{13}C\{^1H\}$ NMR spectrum (CD_2Cl_2, 298 K, 300 MHz) of the same polymeric wax.

Table S2. Productivity of complexes **4a-d** in ethylene polymerization.[a]

[Pd]	Major product	g PE / g Pd	kg PE/ mol Pd
**4a**	butenes	<1.0	<0.1
**4b**	polymer	1.4	0.15
**4c**	short oligomers	<1.0	<0.1
**4d**	polymer	101.5	10.8

[a] Reaction conditions: [**Pd**] (10 µmol), NaBArF (22.2 mg, 25 µmol) in anhydrous 1,2-dichloroethane (10 mL), continuous supply of ethylene (1 atm). PE = polyethylene.

Representative evaluation of ethylene conversion by GC–MS analysis

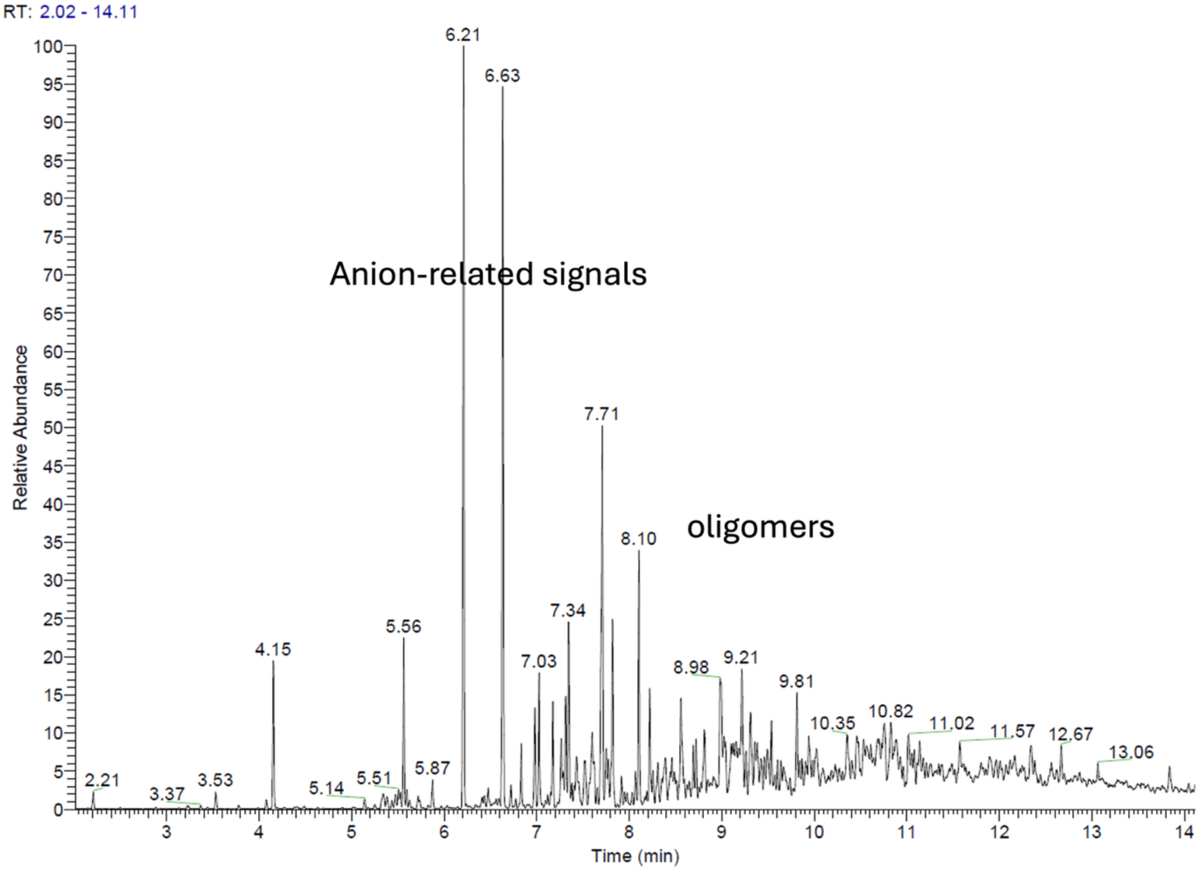

Figure S64: GC-TIC trace of the reaction mixture obtained by reaction of ethylene and **4c** in the presence of NaBArF (2.5 eq *vs* Pd) after 16 h (alkyl/vinyl = 27/1; linear/branched = 1/2, *cf* Table 1, entry 8 of main text). The MS traces of the different oligomeric product bands are shown in Fig. S65–S76.

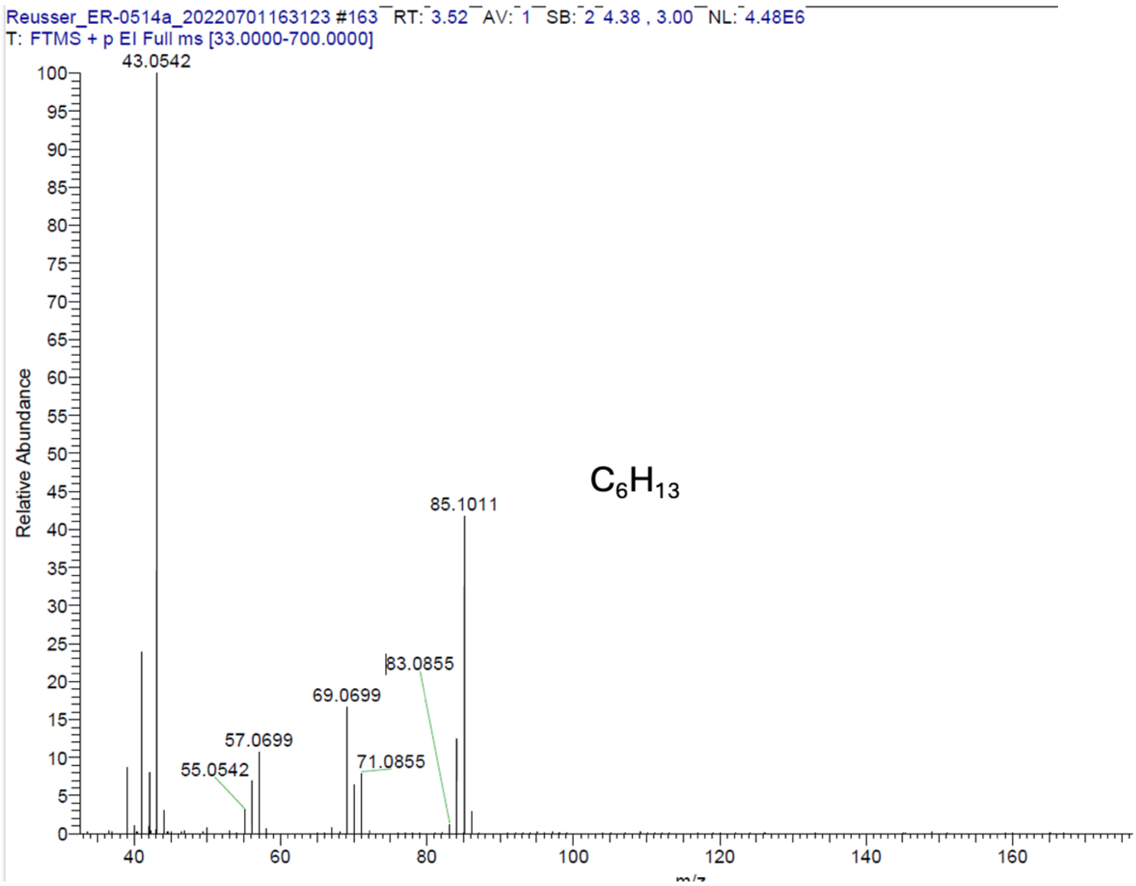

Figure S65: EI-MS trace of the species eluting at t = 3.52 min (*cf*. Fig. S64).

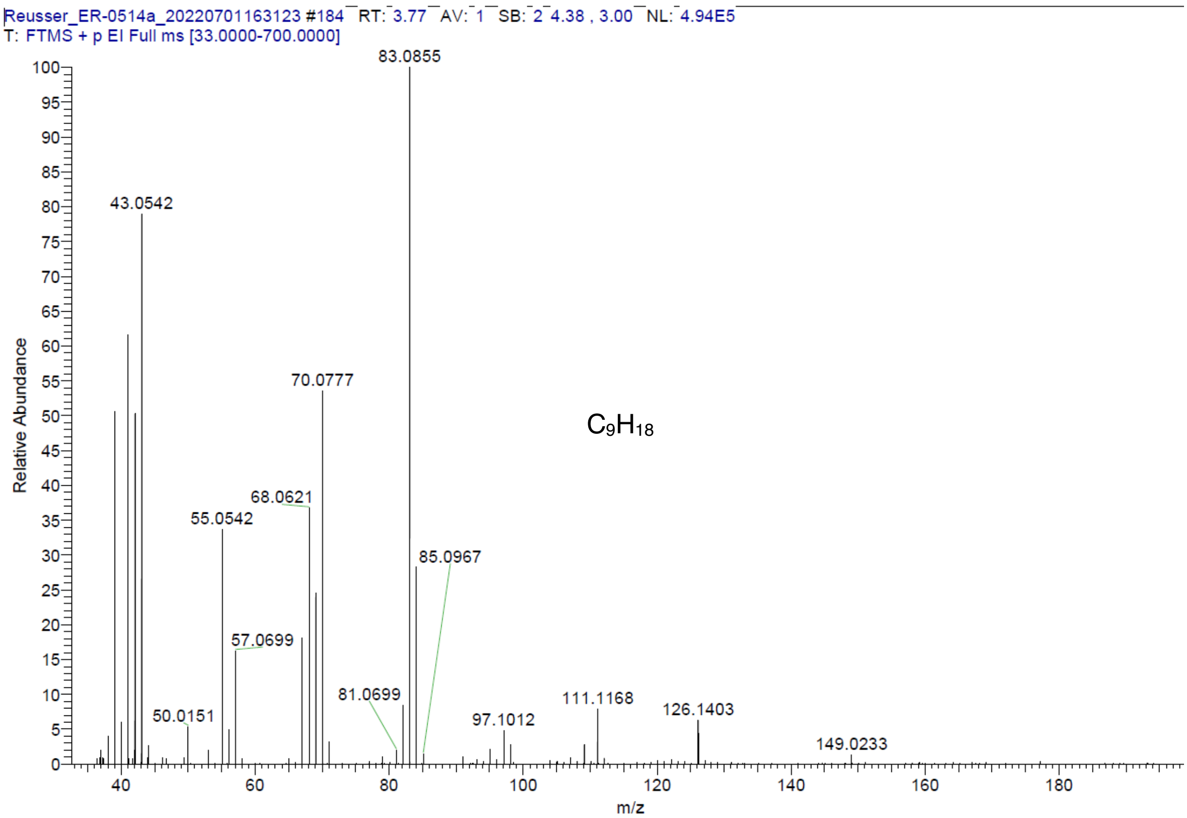

Figure S66: EI-MS trace of the species eluting at t = 3.77 min (*cf*. Fig. S64).

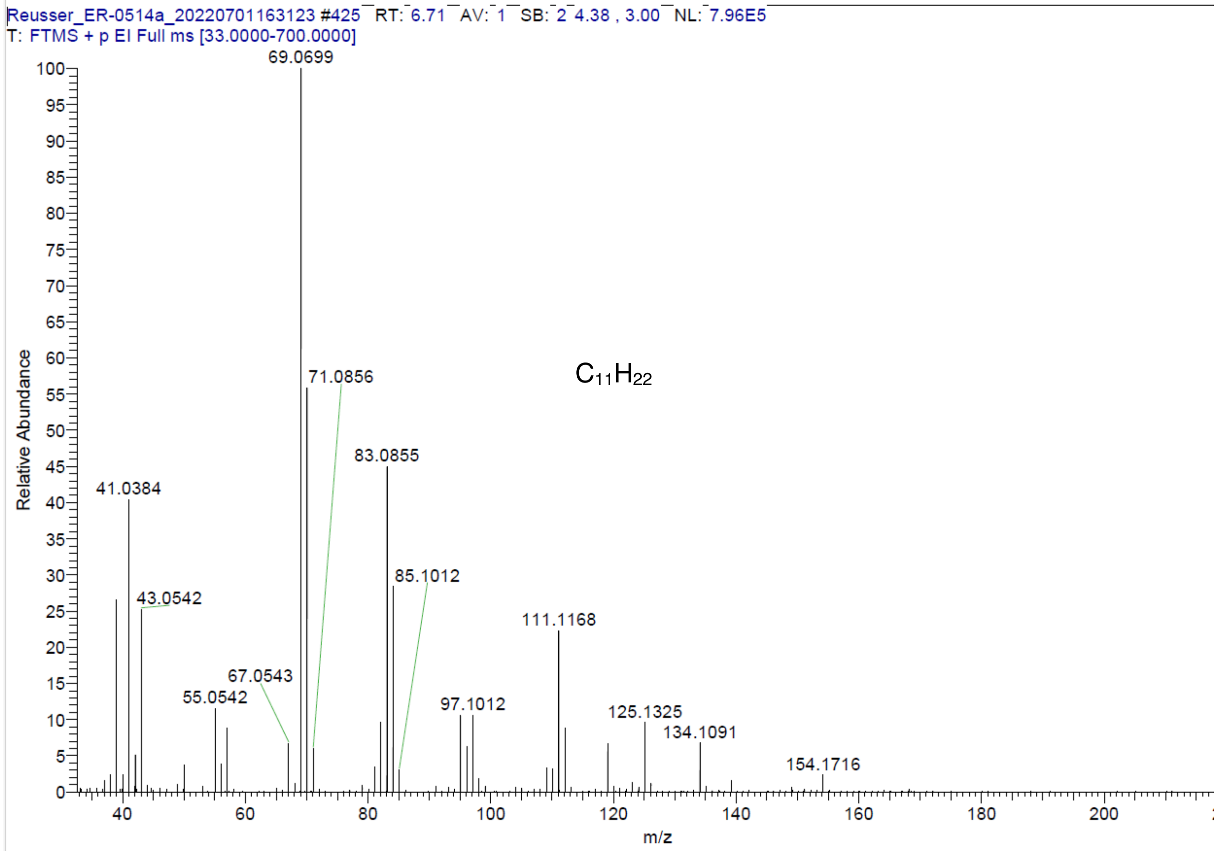

Figure S67: EI-MS trace of the species eluting at t = 6.71 min (*cf*. Fig. S64).

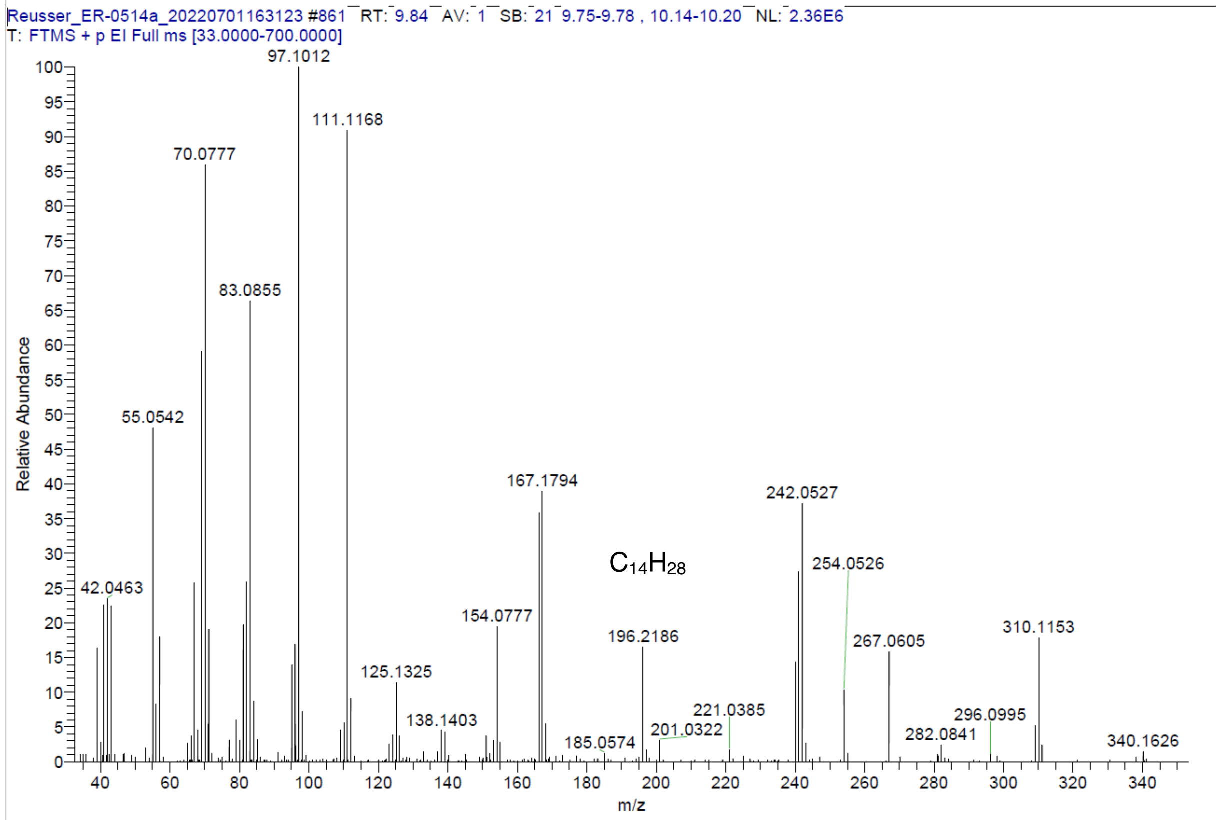

Figure S68: EI-MS trace of the species eluting at t = 9.84 min (*cf*. Fig. S64).

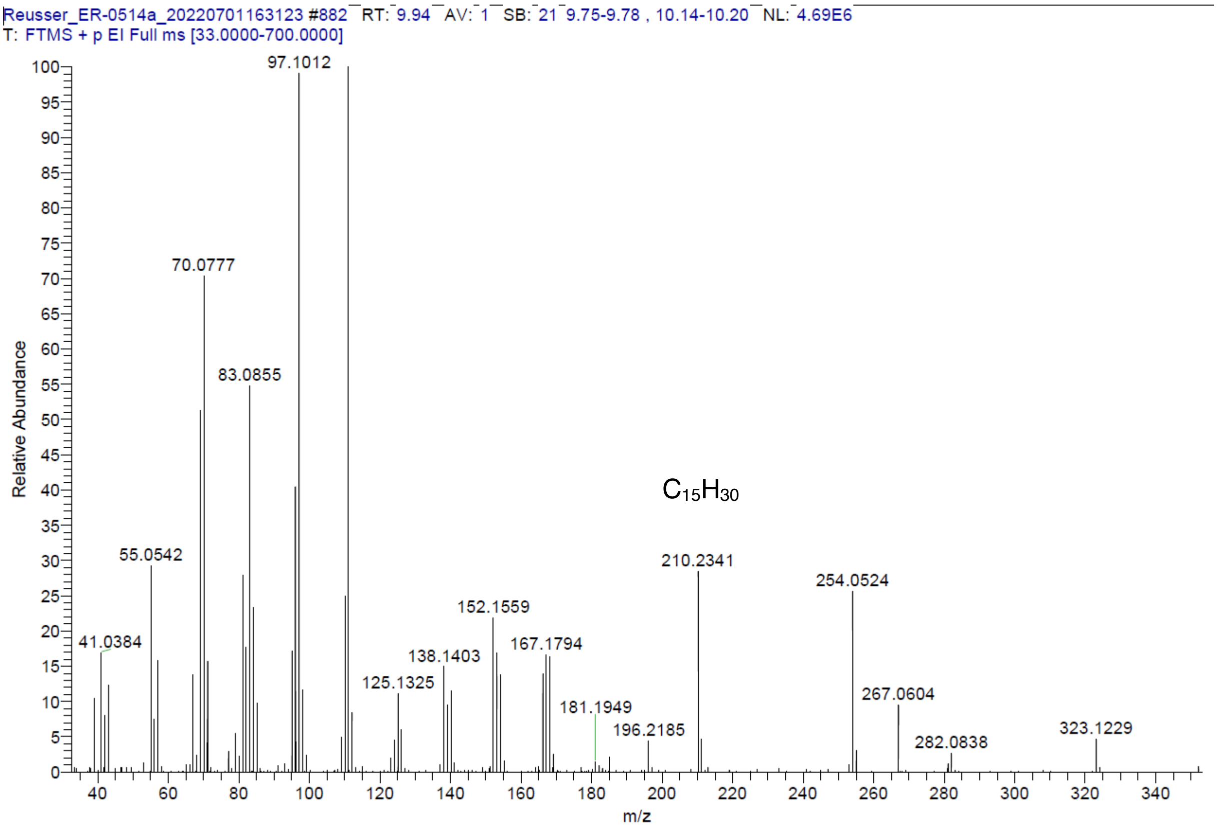

Figure S69: EI-MS trace of the species eluting at t = 9.94 min (*cf.* Fig. S64).

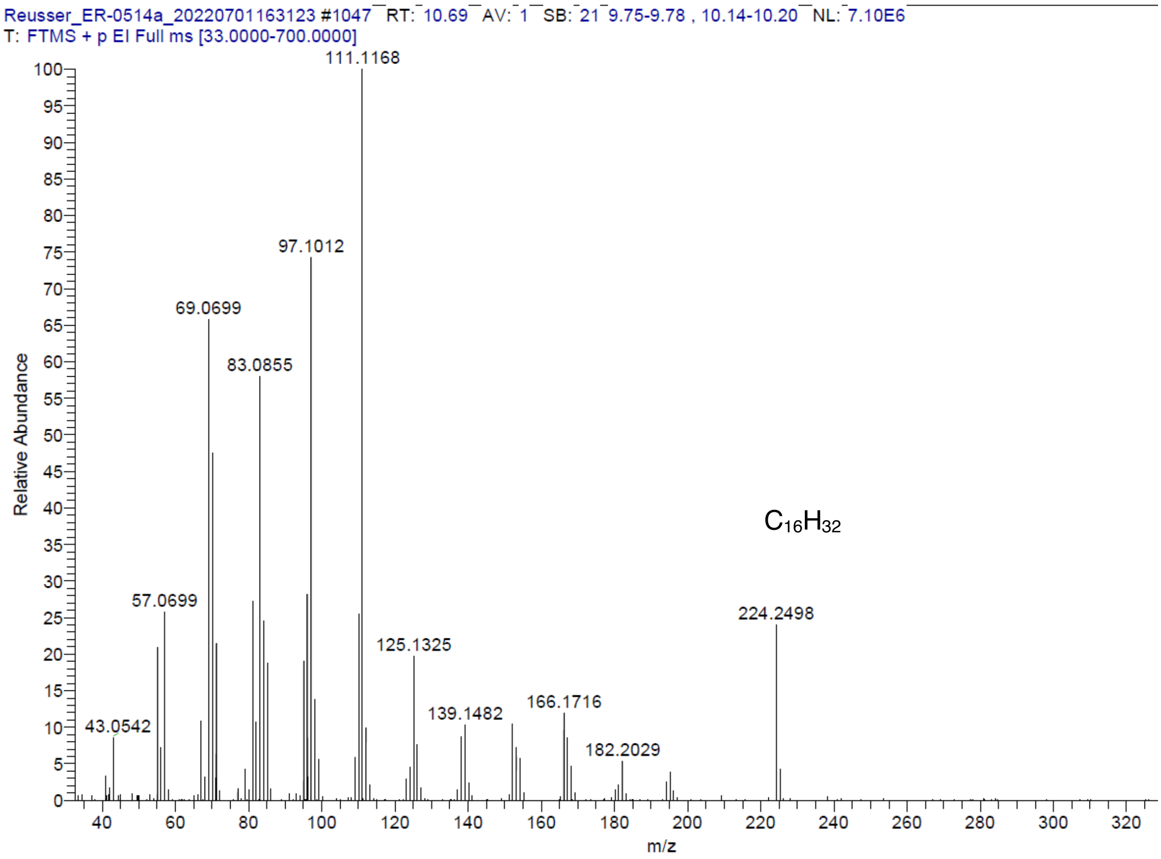

Figure S70: EI-MS trace of the species eluting at t = 10.69 min (*cf.* Fig. S64).

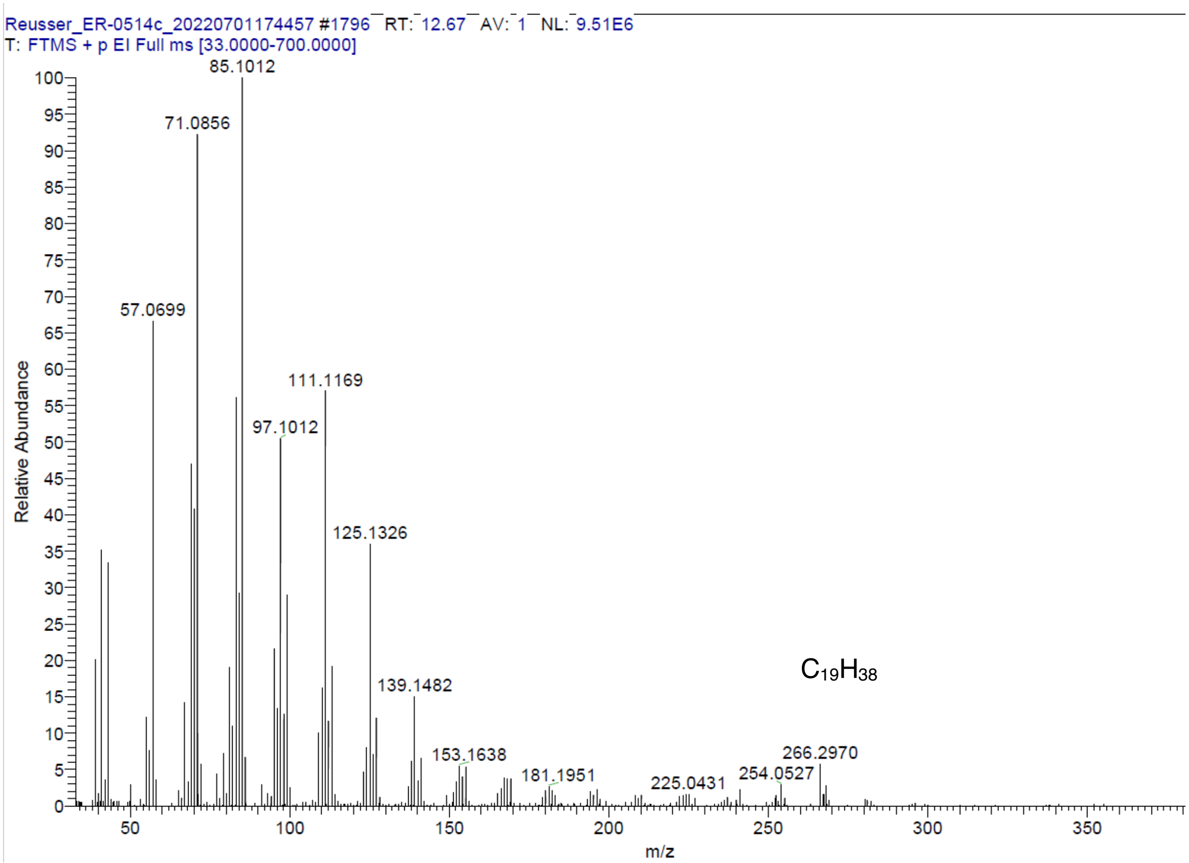

Figure S71: EI-MS trace of the species eluting at t = 12.67 min (*cf*. Fig. S64).

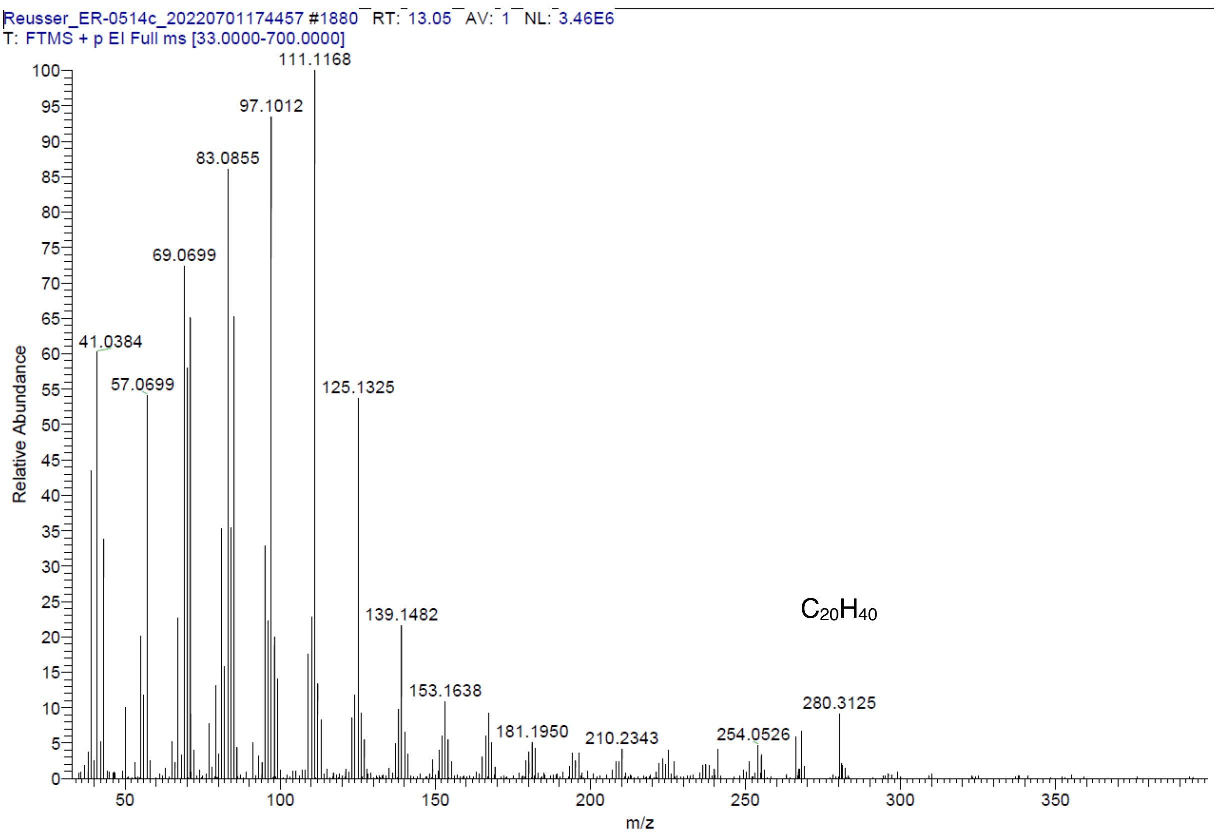

Figure S72: EI-MS trace of the species eluting at t = 13.05 min (*cf*. Fig. S64).

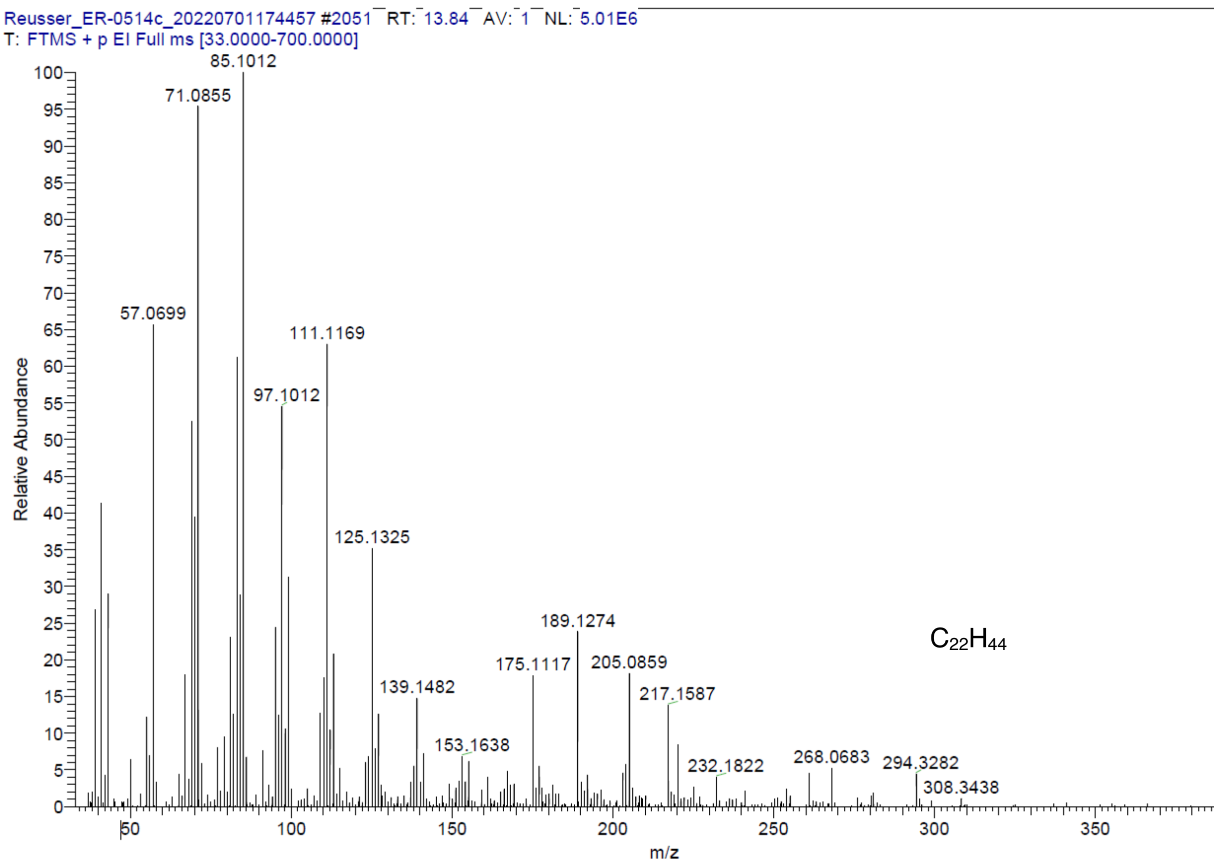

Figure S73: EI-MS trace of the species eluting at t = 13.84 min (*cf.* Fig. S64).

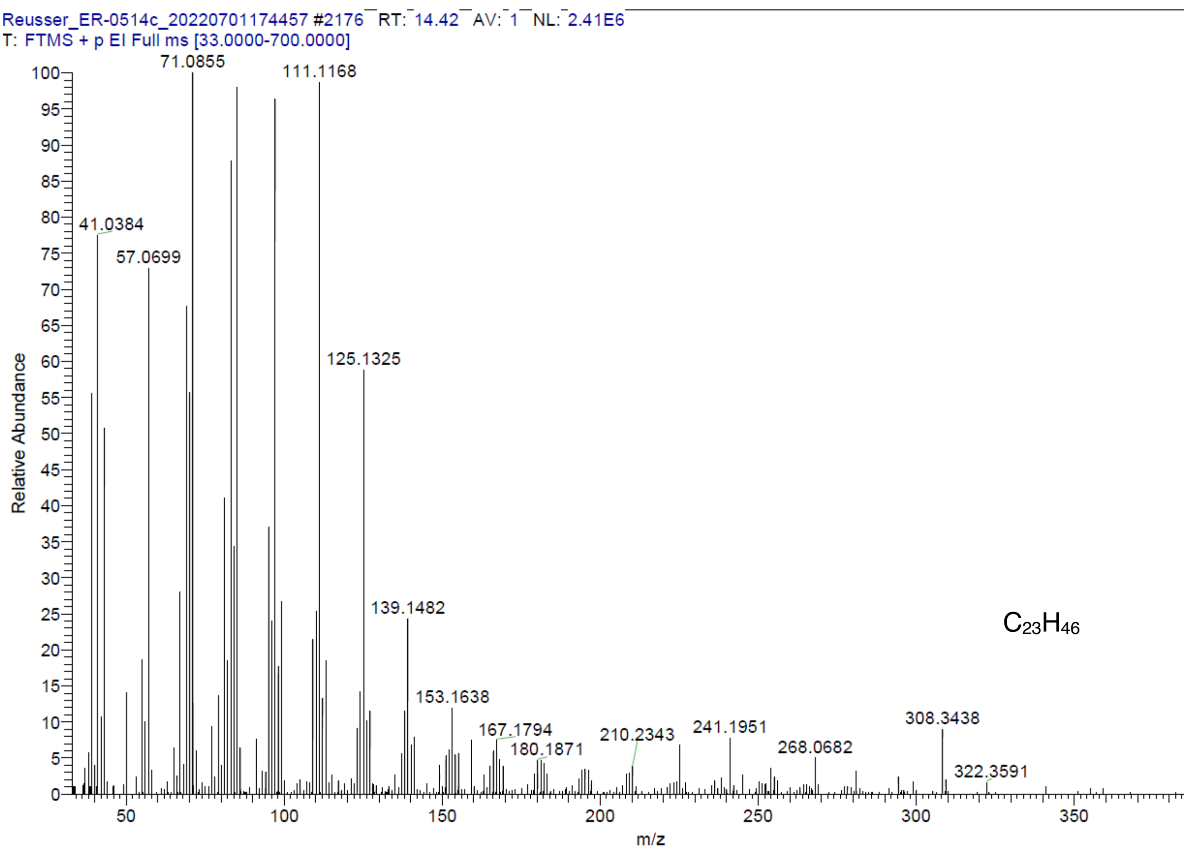

Figure S74: EI-MS trace of the species eluting at t = 14.42 min (*cf.* Fig. S64).

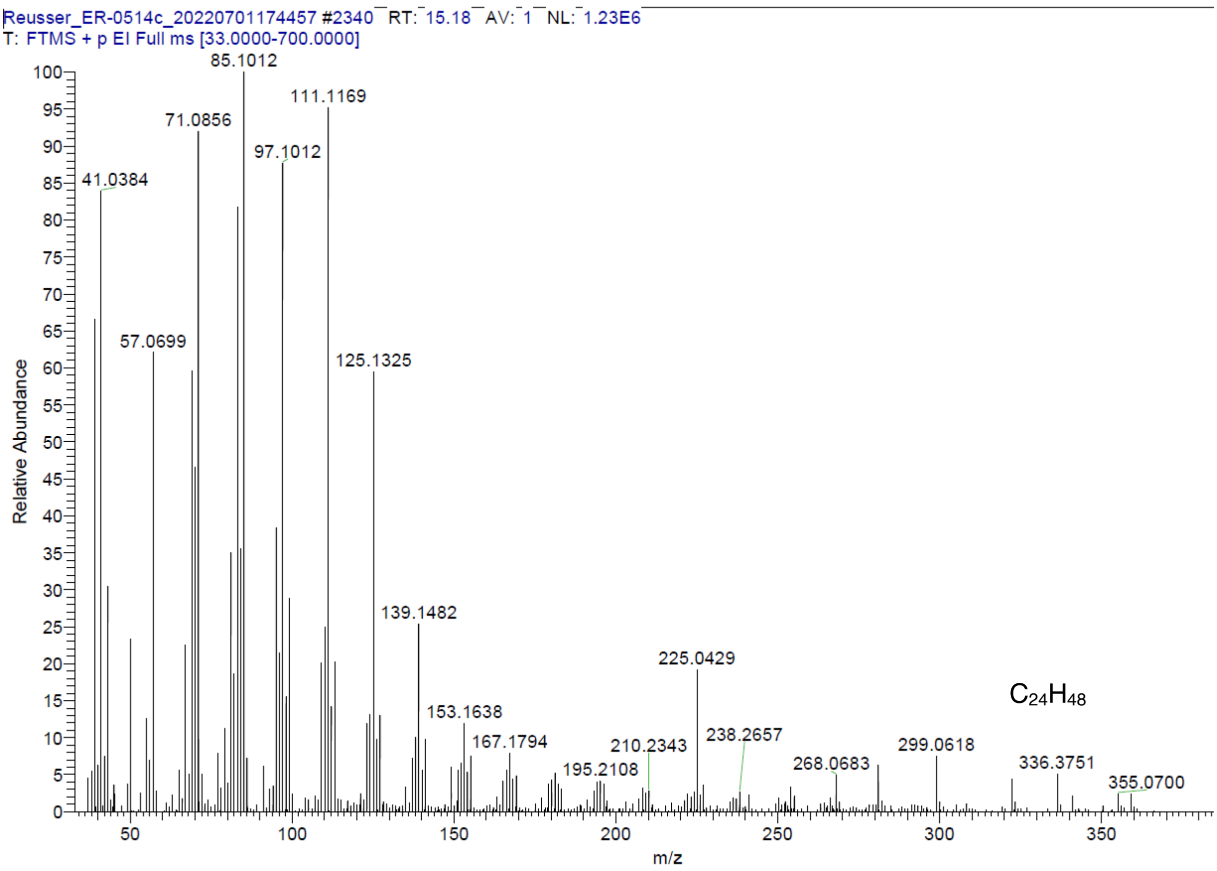

Figure S75: EI-MS trace of the species eluting at t = 15.18 min (*cf.* Fig. S64).

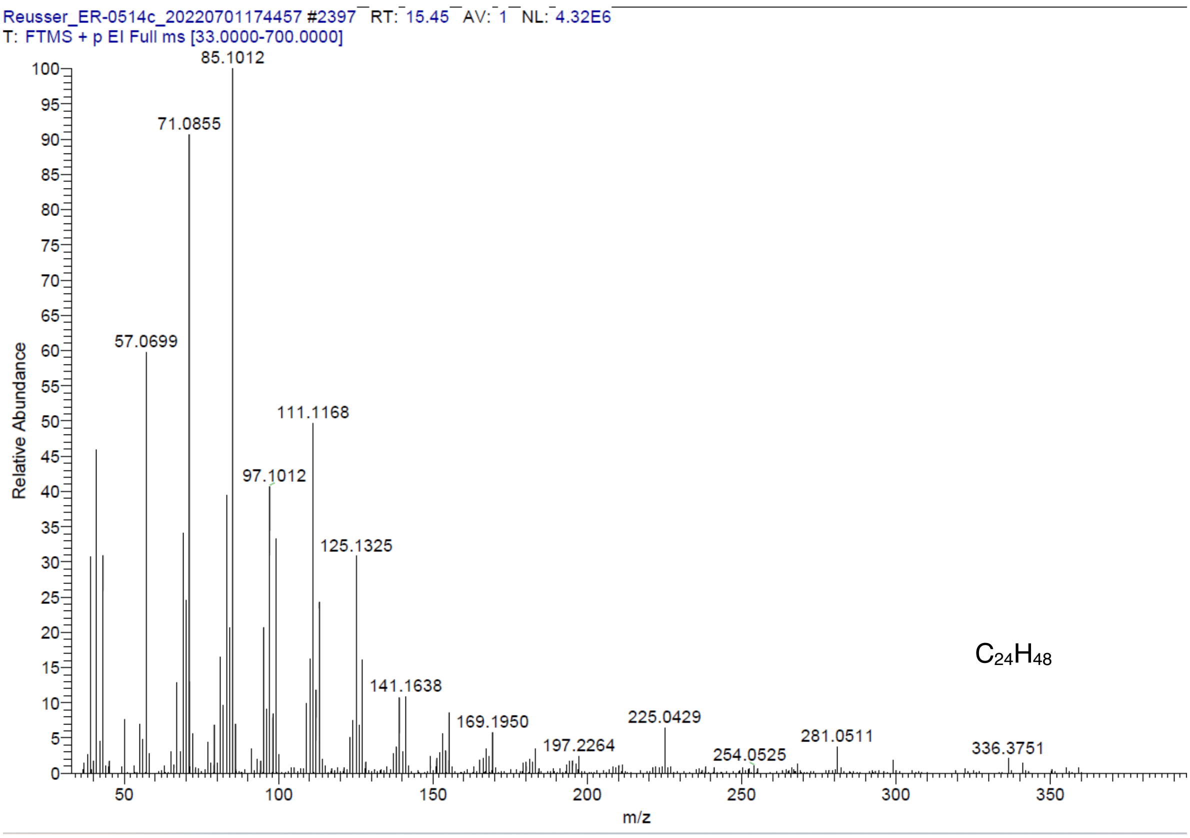

Figure S76: EI-MS trace of the species eluting at t = 15.45 min (*cf.* Fig. S64).

S.3. Crystal structure determinations

Crystals of **2b**, **2d**, **3c** and **6d** immersed in parabar oil were mounted at ambient conditions and transferred into a stream of nitrogen (173 K). Measurements were made on a RIGAKU Synergy S area-detector diffractometer using mirror optics monochromated Cu Kα radiation (λ = 1.54184 Å).
Data reduction was performed using the *CrysAlisPro*[S17] program. The intensities were corrected for Lorentz and polarization effects, and an absorption correction based on the multi-scan method using SCALE3 ABSPACK in *CrysAlisPro*[S17] was applied.
All the structures were solved by intrinsic phasing using *SHELXT*,[S18] which revealed the positions of all non-hydrogen atoms of the title compound. All non-hydrogen atoms were refined anisotropically. For **2b**, **2d** and **6d**, H-atoms were assigned in geometrically calculated positions and refined using a riding model where each H-atom was assigned a fixed isotropic displacement parameter with a value equal to 1.2Ueq of its parent atom (1.5Ueq for methyl groups). For **3c**, H-atoms were located from the difference density map and had their positions and isotropic displacement parameters refined freely.
Refinements were carried out on F^2 using full-matrix least-squares procedures, which minimized the function $\Sigma w(F_o^2 - F_c^2)^2$. The weighting scheme was based on counting statistics and included a factor to downweight the intense reflections. All calculations were performed using the *SHELXL-2014/7*[S19] program in OLEX2.[S20] Data collection and refinement parameters for **2b, 2b, 3c** and **6d** are given in Tables S3 and S4. Crystallographic data for all structures reported in this paper have been deposited with the Cambridge Crystallographic Data Centre (CCDC) as supplementary publication numbers 2440549–2440552.
For **2b** the disorder in a PF_6 anion was treated by splitting fluorine atoms into two part and using free variable for refinement of occupancy. Final occupancies are listed in Table S5. For the structure of **2d**, a disorder model was used for part of the structure where the occupancies of each disorder component was refined through the use of a free variable. The sum of equivalent components was constrained to 1 (100%). Final occupancies are shown in Table S6. For **6d**, large areas containing disorder solvents and possibly half of the PF_6 counter ion were found where a satisfactory model could not be achieved, leading to an overall charge of +0.5 for the asymmetric unit which is an artefact of the unmodeled half anion. A solvent mask was used to include the contribution of electron density found in void areas into the calculated structure factor. The total number of electrons found in the void areas is shown in Table S8. Disorder models were used together with restraints as required for parts of the molecule and PF_6 units where the occupancies of each disorder component was refined through the use of a free variable. The sum of equivalent components was constrained to 1, i.e. 100%. Final occupancies can be seen on Table S7.

Table S3. Crystal data and structure refinement for **2b** and **2d**.

CCDC No.	2440549	2440550
Empirical formula	$C_{18}H_{24}F_{12}N_4O_2P_2$	$C_{16}H_{20}F_{12}N_4O_2P_2$
Formula weight	618.35	590.30
Temperature/K	173.01(10)	173.1(5)
Crystal system	monoclinic	monoclinic
Space group	$P2_1/n$	$P2_1/c$
a/Å	5.83359(4)	7.13858(7)
b/Å	11.41940(9)	21.77525(16)
c/Å	18.48940(14)	7.76094(8)
α/°	90	90
β/°	93.5921(7)	108.0825(11)
γ/°	90	90
Volume/Å^3	1229.272(16)	1146.811(19)
Z	2	2
ρ_{calc}g/cm^3	1.671	1.709
μ/mm^{-1}	2.712	2.875
F(000)	628.0	596.0
Crystal size/mm^3	0.254 × 0.211 × 0.131	0.282 × 0.121 × 0.081
Radiation	Cu Kα (λ = 1.54184)	Cu Kα (λ = 1.54184)
2Θ range for data collection/°	9.108 to 148.456	8.12 to 148.972
Index ranges	$-5 \leq h \leq 7$, $-13 \leq k \leq 14$, $-23 \leq l \leq 23$	$-8 \leq h \leq 8$, $-27 \leq k \leq 27$, $-7 \leq l \leq 9$
Reflections collected	12824	27914
Independent reflections	2503 [R_{int} = 0.0247, R_{sigma} = 0.0154]	2336 [R_{int} = 0.0525, R_{sigma} = 0.0150]
Data/restraints/parameters	2503/12/213	2336/76/265
Goodness-of-fit on F^2	1.068	1.078
Final R indexes [I>=2σ (I)]	R_1 = 0.0563, wR_2 = 0.1492	R_1 = 0.0364, wR_2 = 0.0958
Final R indexes [all data]	R_1 = 0.0578, wR_2 = 0.1501	R_1 = 0.0369, wR_2 = 0.0962
Largest diff. peak/hole / e Å^{-3}	0.91/-0.78	0.36/-0.24

Table S4. Crystal data and structure refinement for ligand **3c** and complex **6d**.

Identification code	2440551	2440552
Empirical formula	$C_{14}H_{14}N_4O_2$	$C_{38}H_{48}F_{15}KN_{10}O_4P_{2.5}Pd_2$
Formula weight	270.29	1323.19
Temperature/K	173.01(10)	173.00(10)
Crystal system	monoclinic	monoclinic
Space group	$P2_1/c$	$P2_1/c$
a/Å	7.29001(8)	21.4383(2)
b/Å	24.73633(20)	22.0849(3)
c/Å	7.53443(8)	13.37677(12)
α/°	90	90
β/°	113.3237(13)	102.7682(10)
γ/°	90	90
Volume/Å^3	1247.64(2)	6176.78(11)
Z	4	4
ρ_{calc}g/cm^3	1.439	1.423
μ/mm^{-1}	0.823	6.694
F(000)	568.0	2646.0
Crystal size/mm^3	0.193 × 0.148 × 0.147	0.228 × 0.122 × 0.093
Radiation	Cu Kα (λ = 1.54184)	Cu Kα (λ = 1.54184)
2Θ range for data collection/°	7.148 to 141.71	5.82 to 151.148
Index ranges	$-8 \leq h \leq 8$, $-30 \leq k \leq 30$, $-9 \leq l \leq 9$	$-26 \leq h \leq 26$, $-27 \leq k \leq 27$, $-15 \leq l \leq 16$
Reflections collected	24882	125948
Independent reflections	2399 [R_{int} = 0.0272, R_{sigma} = 0.0106]	12833 [R_{int} = 0.0499, R_{sigma} = 0.0255]
Data/restraints/parameters	2399/0/238	12833/480/864
Goodness-of-fit on F^2	1.052	1.068
Final R indexes [I>=2σ (I)]	R_1 = 0.0320, wR_2 = 0.0845	R_1 = 0.0604, wR_2 = 0.1887
Final R indexes [all data]	R_1 = 0.0329, wR_2 = 0.0851	R_1 = 0.0662, wR_2 = 0.1972
Largest diff. peak/hole / e Å^{-3}	0.25/-0.17	0.99/-0.94

Table S5. Atomic Occupancies for **2b**

Atom	Occupancy	Atom	Occupancy
F1A	0.393(8)	F1B	0.607(8)
F2A	0.393(8)	F2B	0.607(8)
F3A	0.393(8)	F3B	0.607(8)
F4A	0.393(8)	F4B	0.607(8)

Table S6. Atomic Occupancies for **2d**

Atom	Occupancy	Atom	Occupancy
C3	0.35(2)	C3A	0.65(2)
N2	0.35(2)	N2A	0.65(2)
O1	0.551(11)	O1A	0.449(11)
F3	0.551(11)	F3A	0.449(11)
F4	0.551(11)	F4A	0.449(11)
F5	0.551(11)	F5A	0.449(11)
F6	0.551(11)	F6A	0.449(11)

Table S7. Atomic Occupancies for **6d**.

Atom	Occupancy	Atom	Occupancy	Atom	Occupancy
Pd2	0.430(8)	F10	0.396(19)	F8	0.396(19)
F7	0.396(19)	F9	0.396(19)	C36	0.430(8)
H36A	0.430(8)	H36B	0.430(8)	H36C	0.430(8)
F11	0.396(19)	N10	0.430(8)	F12	0.396(19)
C37	0.430(8)	C38	0.430(8)	H38A	0.430(8)
H38B	0.430(8)	H38C	0.430(8)	N10A	0.570(8)
C37A	0.570(8)	C38A	0.570(8)	H38D	0.570(8)
H38E	0.570(8)	H38F	0.570(8)	P3	0.25
F16	0.25	F17	0.25	F18	0.25
F15	0.25	F14	0.25	F13	0.25
F9A	0.604(19)	F12A	0.604(19)	F7A	0.604(19)
F11A	0.604(19)	F10A	0.604(19)	F8A	0.604(19)
Pd2A	0.570(8)	C36A	0.570(8)	H36D	0.570(8)
H36E	0.570(8)	H36F	0.570(8)	P4	0.25
F24	0.25	F19	0.25	F22	0.25
F20	0.25	F21	0.25	F23	0.25

Table S8. Solvent mask information for **6d**.

Number	X	Y	Z	Volume	Electron count
1	0.288	0.750	-0.189	548.2	153.5
2	0.712	0.250	-0.512	548.2	152.5

S.4. References

S1 K. Salzmann, C. Segarra and M. Albrecht, *Angew. Chem. Int. Ed.*, 2020, **59**, 8932–8936.

S2 W. He, F. Du, Y. Wu, Y. Wang, X. Liu, H. Liu and X. Zhao, *J. Fluor. Chem.*, 2006, **127**, 809–815.

S3 N. M. Do, M. A. Olivier, J. J. Salisbury and C. B. Wager, *Anal. Chem.*, 2011, **83**, 8766–8771.

S4 A. O. Mattes, D. Russell, E. Tishchenko, Y. Liu, R. H. Cichewicz and S. J. Robinson, *Concepts Magn. Reson. Part A*, 2016, **45A**, e21422.

S5 K. Salzmann, C. Segarra, A. Carrel and M. Albrecht, *ChemistryEurope*, 2024, **2**, e202300070.

S6 M. T. Huggins, T. Kesharwani, J. Buttrick and C. Nicholson, *J. Chem. Educ.*, 2020, **97**, 1425–1429.

S7 V. Rosar, A. Meduri, T. Montini, P. Fornasiero, E. Zangrando and B. Milani, *Dalton Trans.*, 2018, **47**, 2778–2790.

S8 V. Khlebnikov, A. Meduri, H. Mueller-Bunz, T. Montini, P. Fornasiero, E. Zangrando, B. Milani and M. Albrecht, *Organometallics*, 2012, **31**, 976–986.

S9 E. Reusser, B. Milani and M. Albrecht, *Organometallics*, 2025, **44**, 1176–1185.

S10 R. Nakano and K. Nozaki, *J. Am. Chem. Soc.*, 2015, **137**, 10934–10937.

S11 P. S. Pregosin, *Pure Appl. Chem.*, 2009, **81**, 615–633.

S12 P. S. Pregosin, *Magn. Reson. Chem.*, 2017, **55**, 405–413.

S13 R. Nanda and K. Damodaran, *Magn. Reson. Chem.*, 2018, **56**, 62–72.

S14 G. M. Ó Máille, A. Dall'Anese, P. Grossenbacher, T. Montini, B. Milani and M. Albrecht, *Dalton Trans.*, 2021, **50**, 6133–6145.

S15 B. Ding, G. Chang, Z. Yan and S. Dai, *Front. Chem.*, DOI:10.3389/fchem.2022.961426.

S16 J. Chen, Z. Yan, Z. Li and S. Dai, *Polymers*, 2022, **14**, 3049.

S17 Oxford Diffraction (2018). CrysAlisPro (Version 1.171.40.37a). Oxford Diffraction Ltd., Yarnton, Oxfordshire, UK.

S18 G. M. Sheldrick, *Acta Crystallogr.*, 2015, **A71**, 3–8.

S19 G. M. Sheldrick, *Acta Crystallogr*, 2015, **C71**, 3–8.

S20 O. V. Dolomanov, L. J. Bourhis, R. J. Gildea, J. A. K. Howard and H. Puschmann, *J. Appl. Crystallogr.*, 2009, **42**, 339–341.
